# A population genetic interpretation of GWAS findings for human quantitative traits

**DOI:** 10.1371/journal.pbio.2002985

**Published:** 2018-03-16

**Authors:** Yuval B. Simons, Kevin Bullaughey, Richard R. Hudson, Guy Sella

**Affiliations:** 1 Department of Biological Sciences, Columbia University, New York, New York, United States of America; 2 Department of Ecology & Evolution, University of Chicago, Chicago, Illinois, United States of America; Georgia Tech, United States of America

## Abstract

Human genome-wide association studies (GWASs) are revealing the genetic architecture of anthropomorphic and biomedical traits, i.e., the frequencies and effect sizes of variants that contribute to heritable variation in a trait. To interpret these findings, we need to understand how genetic architecture is shaped by basic population genetics processes—notably, by mutation, natural selection, and genetic drift. Because many quantitative traits are subject to stabilizing selection and because genetic variation that affects one trait often affects many others, we model the genetic architecture of a focal trait that arises under stabilizing selection in a multidimensional trait space. We solve the model for the phenotypic distribution and allelic dynamics at steady state and derive robust, closed-form solutions for summary statistics of the genetic architecture. Our results provide a simple interpretation for missing heritability and why it varies among traits. They predict that the distribution of variances contributed by loci identified in GWASs is well approximated by a simple functional form that depends on a single parameter: the expected contribution to genetic variance of a strongly selected site affecting the trait. We test this prediction against the results of GWASs for height and body mass index (BMI) and find that it fits the data well, allowing us to make inferences about the degree of pleiotropy and mutational target size for these traits. Our findings help to explain why the GWAS for height explains more of the heritable variance than the similarly sized GWAS for BMI and to predict the increase in explained heritability with study sample size. Considering the demographic history of European populations, in which these GWASs were performed, we further find that most of the associations they identified likely involve mutations that arose shortly before or during the Out-of-Africa bottleneck at sites with selection coefficients around *s* = 10^−3^.

## Author summary

One of the central goals of evolutionary genetics is to understand the processes that give rise to phenotypic variation in humans and other taxa. Genome-wide association studies (GWASs) in humans provide an unprecedented opportunity in that regard, revealing the genetic basis of variation in numerous traits. However, exploiting this opportunity requires models that relate genetic and population genetic processes with the discoveries emerging from GWASs. We present such a model and show that it can help explain the results of GWASs for height and body mass index. More generally, our results offer a simple interpretation of the findings emerging from GWASs and suggest how they relate to the evolutionary and genetic forces that give rise to phenotypic variation.

## Introduction

Much of the phenotypic variation in human populations, including variation in morphological, life history, and biomedical traits, is “complex” or “quantitative”, in the sense that heritable variation in the trait is largely due to small contributions from many genetic variants segregating in the population [1,2]. Quantitative traits have been studied since the birth of biometrics over a century ago [1–3], but only in the past decade have technological advances made it possible to systematically dissect their genetic basis [4–6]. Notably, since 2007, genome-wide association studies (GWASs) in humans have led to the identification of many thousands of variants reproducibly associated with hundreds of quantitative traits, including susceptibility to a wide variety of diseases [4]. While still ongoing, these studies already provide important insights into the genetic architecture of quantitative traits, i.e., the number of variants that contribute to heritable variation, as well as their frequencies and effect sizes.

Perhaps the most striking observation to emerge from these studies is that, despite the large sample size of many GWASs, all variants significantly associated with a given trait typically account for less (often much less) than 25% of the narrow sense heritability ([4,7,8], but see [9]). (Henceforth, we use “heritability” to refer to narrow sense heritability.) While many factors have been hypothesized to contribute to the “missing heritability” [7,8,10–14], the most straightforward explanation and the emerging consensus is that much of the heritable variation derives from variants with frequencies that are too low or effect sizes that are too small for current studies to detect. Comparisons among traits also suggest that there are substantial differences in architectures. For example, recent meta-analysis GWASs uncovered 7 times as many variants for height (697) as for body mass index (97), and together, the variants for height account for more than 4 times the heritable variance than the variants for body mass index do (approximately 20% versus approximately 3%–5%, respectively), despite comparable sample sizes [15,16].

These first glimpses underscore the need for theory that relates the findings emerging from GWASs with the evolutionary processes that shape genetic architectures. Such theory would help to interpret the “missing heritability” [17–20] and to explain why architecture differs among traits. It may also allow us to use GWAS findings to make inferences about underlying evolutionary parameters, helping to answer enduring questions about the processes that maintain phenotypic variation in quantitative traits [5,21].

Development of such theory can be guided by empirical observations and first-principles considerations. New mutations affecting a trait arise at a rate that depends on its “mutational target size” (i.e., the number of sites at which a mutation would affect the trait). Once they arise, the trajectories of variants through the population are determined by the interplay between genetic drift, demographic processes, and natural selection acting on them. These processes determine the number and frequencies of segregating variants underlying variation in the trait. The genetic architecture further depends on the relationship between the selection on variants and their effects on the trait. Notably, selection on variants depends not only on their effect on the focal trait but also on their pleiotropic effects on other traits. We therefore expect both direct and pleiotropic selection to shape the joint distribution of allele frequencies and effect sizes.

Multiple lines of evidence suggest that many quantitative traits are subject to stabilizing selection, i.e., selection favoring an intermediate trait value [5,22–27]. For instance, a decline in fitness components (e.g., viability and fecundity) is observed with displacement from mean values for a variety of traits in human populations [28–30], in other species in the wild [31,32], and in experimental manipulations [31,33]. While less is known about complex diseases, they may often reflect large deviations of an underlying continuous trait from an optimal value [1], with these continuous traits subject to directional (purifying) selection in some cases and to stabilizing selection in others. What remains unclear is the extent to which stabilizing selection is acting directly on variation in a given trait or is “apparent”, i.e., results from pleiotropic effects of this variation on other traits.

Other lines of evidence suggest that pleiotropy is pervasive. For one, theoretical considerations about the variance in fitness in natural populations and its accompanying genetic load suggest that only a moderate number of independent traits can be effectively selected on at once [34]. Thus, the aforementioned relationships between the value of a focal trait and fitness are likely heavily affected by the pleiotropic effects of genetic variation on other traits [25,34–36]. Second, many of the variants detected in human GWASs have been found to be associated with more than one trait [37–41]. For example, a recent analysis of GWASs revealed that variants that delay the age of menarche in women tend to delay the age of voice drop in men, decrease body mass index, increase adult height, and decrease risk of male pattern baldness [37]. More generally, the extent of pleiotropy revealed by GWASs appears to be increasing rapidly with improvements in power and methodology [37,42–45]. These considerations and others [45,46] point to the general importance of pleiotropic selection on quantitative genetic variation.

The discoveries emerging from human GWASs further suggest that genetic variance is dominated by additive contributions from numerous variants with small effect sizes. Dominance and epistasis may be common among newly arising mutations of large effect (e.g., [47–51]), but both theory and data suggest that they play a minor role in shaping quantitative genetic variation within populations (e.g., [9,52–56]). Indeed, for many traits, most or all of the heritability explained in GWASs arises from the additive contribution of variants with squared effect sizes that are substantially smaller than the total genetic variance (e.g., [15,16,57,58]). Moreover, statistical quantifications of the total genetic variance tagged by genotyping (i.e., not only due to the genome-wide significant associations) suggest that such contributions may account for most of the heritable variance in many traits (e.g., [9,59–61]). Finally, considerable efforts to detect epistatic interactions in human GWASs have, by and large, come up empty-handed [9,56,62], with few counterexamples, mostly involving variants in the major histocompatibility complex region ([53,56,63,64], but see [65]). Thus, while the discovery of epistatic interactions may be somewhat limited by statistical power [56], theory and current evidence suggest that nonadditive interactions play a minor role in shaping human quantitative genetic variation. Motivated by these considerations, we model how direct and pleiotropic stabilizing selection shape the genetic architecture of continuous, quantitative traits by considering additive variants with small effects and assuming that together they account for most of the heritable variance.

To date, there has been relatively little theoretical work relating population genetics processes with the results emerging from GWASs. Moreover, the few existing models have reached divergent predictions about genetic architecture, largely because they make different assumptions about the effects of pleiotropy. Focusing on disease susceptibility, Pritchard [19] considered the “purely pleiotropic” extreme, in which selection on variants is independent of their effect on the trait being considered. In this case, we expect the largest contribution to genetic variance in a trait to come from mutations that have large effect sizes but are also weakly selected or neutral, allowing them to ascend to relatively high frequencies. Other studies considered the opposite extreme, in which selection on variants stems entirely from their effect on the trait under consideration [26,66–70], and have shown that the greatest contribution to genetic variance would arise from strongly selected mutations [67,68] (we return to this case below).

In practice, we expect most traits to fall somewhere in between these extremes. While there are compelling reasons to believe that quantitative genetic variation is highly pleiotropic, the effects of variants on different traits are likely to be correlated. Thus, even if a given trait is not subject to selection, variants that have a large effect on it will also tend to have larger effects on traits that are under selection (e.g., by causing large perturbation to pathways that affect multiple traits [36,45]). Motivated by such considerations, Eyre-Walker (2010) [20], Keightley and Hill (1990) [18], and Caballero et al. (2015) [71] considered models in which the correlation between the strength of selection on an allele and its effect size can vary between the purely pleiotropic and direct selection extremes. These models diverge in their predictions about architecture, however. Assuming, as seems plausible, an intermediate correlation between the strength of selection and effect size, Eyre-Walker finds that genetic variance should be dominated by strongly selected mutations [20], whereas Keightley and Hill and Caballero et al. conclude that the greatest contribution should arise from weakly selected ones [18,71]. Their conclusions differ because of how they chose to model the relationship between selection and effect size, a choice based largely on mathematical convenience. We approach this problem by explicitly modeling stabilizing selection on multiple traits, thereby learning, rather than assuming, the relationship between selection and effect sizes.

## The model

We model stabilizing selection in a multidimensional phenotype space, akin to Fisher’s geometric model [72]. An individual’s phenotype is a vector in an *n*-dimensional Euclidian space, in which each dimension corresponds to a continuous quantitative trait. We focus on the architecture of one of these traits (say, the first dimension), where the total number of traits parameterizes pleiotropy. Fitness is assumed to decline with distance from the optimal phenotype positioned at the origin, thereby introducing stabilizing selection. Specifically, we assume that absolute fitness takes the form

$$\text{W}(\vec{r})=exp(-\frac{r^{2}}{{2w}^{2}}),$$ (1)

where $\vec{r}$ is the (*n*-dimensional) phenotype, $r=\|\vec{r}\|$ is the distance from the origin, and *w* parameterizes the strength of stabilizing selection. However, we later show that the specific form of the fitness function does not matter. Moreover, the additive environmental contribution to the phenotype can be absorbed into *w* ([73]; Section 1.1 in S1 Text); we therefore consider only the genetic contribution.

The genetic contribution to the phenotype follows from the multidimensional additive model [74]. Specifically, we assume that the number of genomic sites affecting the phenotype (the target size) is very large, *L* ≫ 1, and that allelic effects on the phenotype at these sites are vectors in the *n*-dimensional trait space. An individual’s phenotype then follows from adding up the effects of her or his alleles, i.e.,

$$\vec{r}=\sum_{l=1}^{L}\left({\vec{a}}_{l}+{\vec{a}}_{l}^{\prime }\right),$$ (2)

where *${\vec{a}}_{l}$* and *${\vec{a}}_{l}^{\prime }$* are the phenotypic effects of the parents’ alleles at site *l*.

The population dynamics follows from the standard model of a diploid, panmictic population of constant size *N*, with nonoverlapping generations. In each generation, parents are randomly chosen to reproduce with probabilities proportional to their fitness (i.e., Wright-Fisher sampling with viability selection), followed by mutation, free recombination (i.e., no linkage), and Mendelian segregation. We further assume that the mutation rate per site, *u*, and the population size are sufficiently small such that no more than 2 alleles segregate at any time at each site (i.e., that *θ* = 4*Nu* ≪ 1) and therefore an infinite sites approximation applies. The number of mutations per gamete per generation therefore follows a Poisson distribution with mean *U* = *Lu*; based on biological considerations (see Sections 4.1 and 4.2 in S1 Text), we also assume that 1 ≫ *U* ≫ 1/2*N*. The size of mutations in the *n*-dimensional trait space, $a\;\left(=\|\vec{a}\|\right)$, is drawn from some distribution, assuming only that *a*^2^ ≪ *w*^2^. We later show that this requirement is equivalent to the standard assumption about selection coefficients satisfying *s* ≪ 1 (also see Section 4.3 in S1 Text). The directions of mutations are assumed to be isotropic, i.e., uniformly distributed on the hypersphere in *n*-dimensions defined by their size, although we later show that our results are robust to relaxing this assumption as well.

## Results

### The phenotypic distribution

In the first 3 sections, we develop the tools that we later use to study genetic architecture. We start by considering the equilibrium distribution of phenotypes in the population and generalizing previous results for the case with a single trait [26,66,67,70]. Under biologically sensible conditions, this distribution is well approximated by a tight multivariate normal centered at the optimum. Namely, the distribution of *n*-dimensional phenotypes, $\vec{r}$, in the population, is well approximated by the probability density function:

$$\text{f}\left(\vec{r}\right)=\frac{1}{{\left(2\pi \sigma^{2}\right)}^{n/2}}\text{exp}\left(-\frac{r^{2}}{2\sigma^{2}}\right),$$ (3)

where *σ*^2^ is the genetic variance of the phenotypic distances from the optimum (see Eq A25 in S1 Text for closed form); and under plausible assumptions about the rate and size of mutations (i.e., when 1 ≫ *U* ≫ 1/2N and *a*^2^ ≪ *w*^2^), it satisfies *σ*^2^ ≪ *w*^2^, implying small variance in fitness in the population (Section 4.2 in S1 Text). Intuitively, the phenotypic distribution is normal because it derives from additive and (approximately) independently and identically distributed contributions from many segregating sites. Moreover, the population mean remains extremely close to the optimum because stabilizing selection becomes increasingly stronger with the displacement from it and because any displacement is rapidly offset by minor changes to allele frequencies at many segregating sites.

With phenotypes close to the optimum, only the curvature of the fitness function at the optimum (i.e., the multidimensional second derivative) affects the selection acting on individuals. In addition, it is always possible to choose an orthonormal coordinate system centered at the optimum, in which the trait under consideration varies along the first coordinate and a unit change in other traits (along other coordinates) near the optimum has the same effect on fitness. These considerations suggest that the equilibrium behavior is insensitive to our choice of fitness function around the optimum. Moreover, in S1 Text (Section 5), we show that the rapid offset of perturbations of the population mean from the optimum (by minor changes to allele frequencies at numerous sites) lends robustness to the equilibrium dynamics with respect to the presence of major loci, moderate changes in the optimal phenotype over time, and moderate asymmetries in the mutational distribution.

### Allelic dynamic

Next, we consider the dynamic at a segregating site and generalize previous results for the case with a single trait [68–70]. This dynamic can be described in terms of the first 2 moments of change in allele frequency in a single generation (see, e.g., [75]). To calculate these moments for an allele with phenotypic effect $\vec{a}$ and frequency *q* (=1-*p*), we note that the phenotypic distribution can be well approximated as a sum of the expected contribution of the allele to the phenotype, $2q\vec{a}$, and the distribution of contributions to the phenotype from all other sites, $\vec{R}$. From Eq 3, it then follows that the distribution of background contributions is well approximated by probability density:

$$\text{f}\left(\vec{R}|\vec{a},q\right)=\frac{1}{{\left(2\pi \sigma^{2}\right)}^{n/2}}\text{exp}\left(-\frac{{\left(\vec{R}+2q\vec{a}\right)}^{2}}{2\sigma^{2}}\right).$$ (4)

By averaging the fitness of the 3 genotypes at the focal site over the distribution of genetic backgrounds, we find that the first moment is well approximated by

$$\text{E}\left(\Delta q\right)\approx \frac{a^{2}}{w^{2}}pq\left(q-\frac{1}{2}\right),$$ (5)

assuming that *a*^2^ and *σ*^2^ ≪ *w*^2^ (Section 4 in S1 Text). By the same token, we find that

$$\text{V}\left(\Delta q\right)\approx \frac{pq}{2N},$$ (6)

which is the standard second moment with genetic drift.

The functional form of the first moment is equivalent to that of the standard viability selection model with underdominance. This result is a hallmark of stabilizing selection on (additive) quantitative traits: with the population mean at the optimum, the dynamics at different sites are decoupled, and selection at a given site acts to reduce its contribution to the phenotypic variance (2*a*^*2*^*pq*), thereby pushing rare alleles to loss. Comparison with the standard viability selection model shows that the selection coefficient in our model is *s* = *a*^2^/*w*^2^, or *S* = 2*Ns* = 2*Na*^2^/*w*^2^ in scaled units. In other words, the selection acting on an allele is proportional to its size squared in the *n*-dimensional trait space (where *w* translates effect size into units of fitness).

### The relationship between selection and effect size

**Fig 1:**
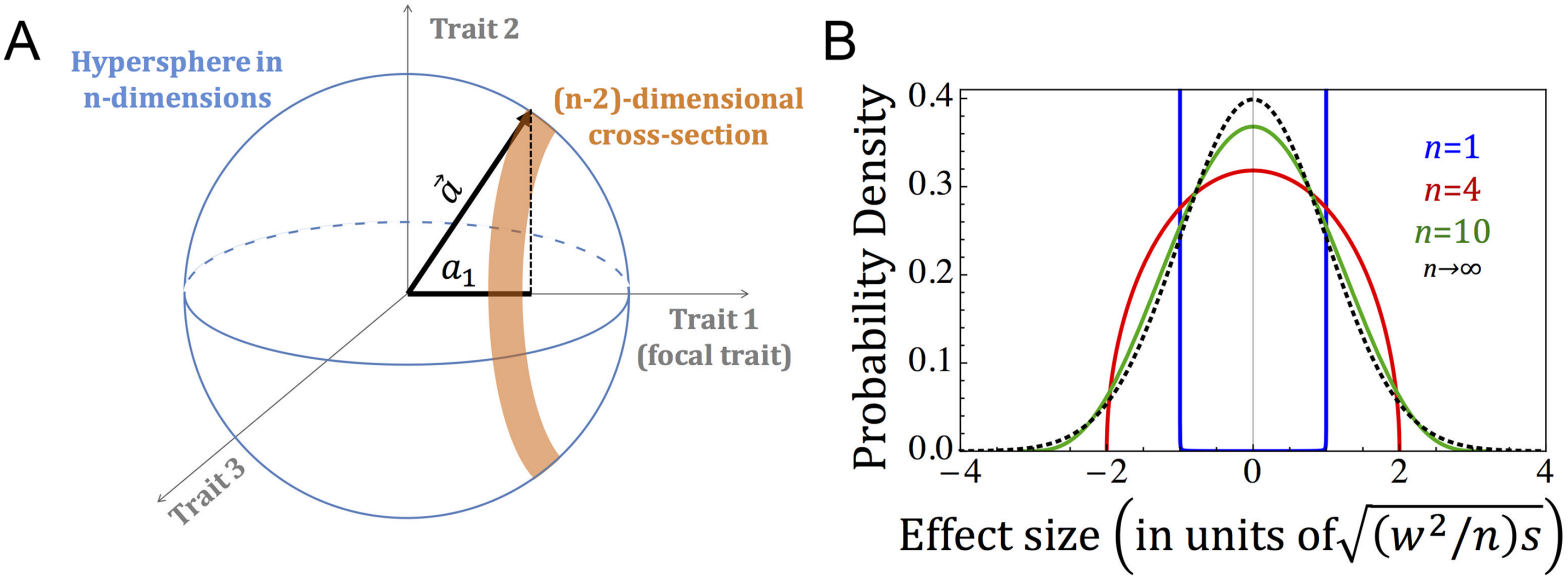
The distribution of effect sizes corresponding to a given selection coefficient. (A) Mutations with selection coefficient, *s*, lie on a hypersphere in *n* dimensions with radius $a=w\sqrt{s}$. The probability that such mutations have effect size *a*_1_ on the focal trait is proportional to the volume of the (**n** − **2**)–dimensional cross section of the hypersphere, with projection *a*_1_ on the coordinate corresponding to the trait. (B) The distribution of effect sizes on the focal trait, conditional on the selection coefficient being *s*, measured in units of the distribution’s standard deviation (see Eq 11).

The statistical relationship between the strength of selection acting on mutations and their effect on a given trait follows from the aforementioned geometric interpretation of selection. Specifically, all mutations with a given selection coefficient, *s*, lie on a hypersphere in *n*-dimensions with radius $a=w\sqrt{s}$, and any given mutation satisfies

$$s=\frac{1}{w^{2}}a^{2}=\frac{1}{w^{2}}\sum_{i=1}^{n}a_{i}^{2},$$ (7)

where *a*_*i*_ is the allele’s effect on the i-th trait (Fig 1A). Our assumption that mutation is isotropic then implies that the probability density of mutations on the hypersphere is uniform.

The distribution of effect sizes on a focal trait, *a*_1_, corresponding to a given selection coefficient, *s*, follows. Given that mutation is symmetric in any given trait, E(*a*_1_|*s*) = 0, and given that it is symmetric among traits,

$$\text{E}\left(a_{1}^{2}|s\right)=a^{2}/n=\left(w^{2}/n\right)s.$$ (8)

More generally, the probability density corresponding to an effect size *a*_1_ is proportional to the volume of the (*n* − 2)–dimensional cross section of the hypersphere with projection *a*_1_ (Fig 1A). For a single trait, this implies that *a*_1_ = ±*a* with probability ½, and for *n* > 1, it implies the probability density

$$\varphi_{n}\left(a_{1}|a\right)=\frac{\Gamma \left(n/2\right)/\Gamma \left(\left(n-1\right)/2\right)}{\sqrt{n/2}\;}\frac{1}{\sqrt{2\pi \left(a^{2}/n\right)}}{\left(1-\frac{1}{n}\frac{a_{1}^{2}}{\left(a^{2}/n\right)}\right)}^{\frac{n-3}{2}}$$ (9)

(Section 1.2 in S1 Text). Intriguingly, when the number of traits *n* increases, this density approaches a normal distribution, i.e.,

$$\frac{a_{1}}{\sqrt{a^{2}/n}}\sim \text{N}\left(0,1\right),$$ (10)

implying that the distribution of effect sizes given the selection coefficient becomes

$$a_{1}\sim \text{N}\left(0,\left(w^{2}/n\right)s\right).$$ (11)

This limit is already well approximated for a moderate number of traits (e.g., *n* = 10; Fig 1B).

The limit behavior also holds when we relax the assumption of isotropic mutation. This generalization is important because, having chosen a parameterization of traits in which the fitness function near the optimum is isotropic, we can no longer assume that the distribution of mutations is also isotropic [76]. Specifically, mutations might tend to have larger effects on some traits than on others, and their effects on different traits might be correlated. In Section 5.4 in S1 Text, we show that the limit distribution (Eq 11) also holds for anisotropic mutation (excluding pathological cases). To this end, we introduce the concept of an effective number of traits, *n*_*e*_, which can take any real value ≥1 and is defined as the number of equivalent traits required to generate the same relationship between the strength of selection on mutations and their expected effects on the trait under consideration (i.e., replacing *n* in Eq 11). The robustness of our model, along with mounting evidence that genetic variation is highly pleiotropic (see “Introduction”), suggests that the limit form may apply quite generally. In that regard, we note that even in this limit, the strength of selection on mutations and their effects on the focal trait are correlated, implying that the kind of “purely pleiotropic” extreme postulated in previous works cannot arise [18–20].

### Genetic architecture

We can now derive closed forms for summary statistics of the genetic architecture (see Section 2.3 in S1 Text). For mutations with a given selection coefficient, the frequency distribution follows from the diffusion approximation based on the first 2 moments of change in allele frequency (Eqs 5 and 6; [75]), and the distribution of effect sizes follows from the geometric considerations of the previous section. Conditional on the selection coefficient, these distributions are independent, and therefore, the joint distribution of frequency and effect size equals their product. Summaries of architecture can be expressed as expectations over the joint distribution of frequencies and effect sizes for a given selection coefficient and then weighted according to the distribution of selection coefficients. While we know little about the distribution of selection coefficients of mutations affecting quantitative traits, we can draw general conclusions from examining how summaries of architecture depend on the strength of selection.

### Expected variance per site

We focus on the distribution of additive genetic variances among sites, a central feature of architecture that is key to connecting our model with GWAS results. We start by considering how selection affects the expected contribution of a site to additive genetic variance in a focal trait. We include monomorphic sites in the expectation, such that the expected total variance is given by the product of the expectation per-site and the population mutation rate, 2*NU*. Under the infinite sites assumption, sites are monomorphic or biallelic, and their expected contribution to variance is

$$\text{E}\left(2a_{1}^{2}pq|S\right)=\text{E}\left(a_{1}^{2}|S\right)\text{E}\left(2pq|S\right)=\frac{w^{2}}{2Nn}S\;\text{E}\left(2pq|S\right)$$ (12)

(expressed in terms of the scaled selection coefficient *S*). Thus, the degree of pleiotropy only affects the expectation through a multiplicative constant.

This multiplicative factor would have a discernable effect in generalizations of our model in which the degree of pleiotropy varies among sites. For example, if the degree of pleiotropy of one set of sites was *k* and of another set was *l* > *k*, and both sets were subject to the same strength of selection, then the expected contribution to genetic variance of sites in the first set would be *l*/*k* times greater than in the second (from Eq 12). While such generalizations may prove interesting in the future, here we focus on the model in which the degree of pleiotropy is constant. In this case, the multiplicative factor introduced by pleiotropy is not identifiable from data, because even if we could measure genetic variance in units of fitness (e.g., rather than in units of the total phenotypic variance), we still would not be able to distinguish between the effects of *w* and *n* on the genetic variance per site. We therefore focus on the effect of selection on the relative contribution to variance, which is insensitive to the degree of pleiotropy in our model.

**Fig 2:**
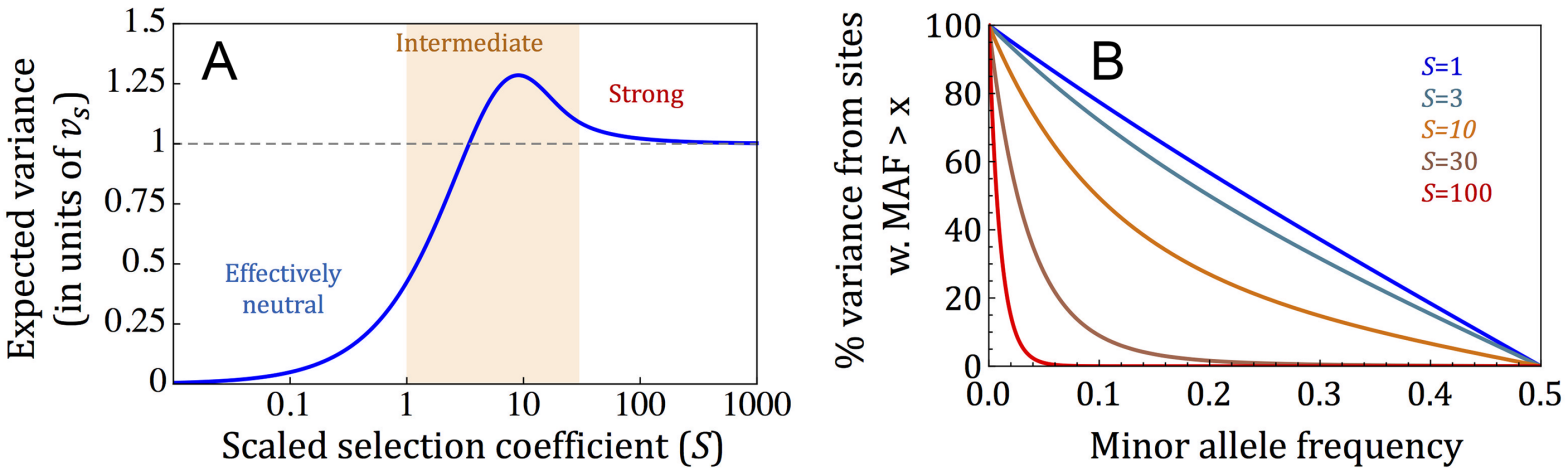
The distribution of additive genetic variance among sites. In (A), we plot the expected contribution as a function of the scaled selection coefficient. We measure genetic variance in units of **v**_**S**_—the expected contribution at sites under strong selection. In (B), we show the proportion of additive genetic variance that arises from sites with minor allele frequency (MAF) greater than the value on the *x*-axis, for different selection coefficients.

The effect of selection on the relative contribution to genetic variance was described by Keightley and Hill (in the one-dimensional case [68]) and is depicted in Fig 2A. When selection is strong (roughly corresponding to *S* > 30), its effect on allele frequency (which scales with 1/*S*) is canceled out by its relationship with the effect size (Eq 8), yielding a constant contribution to genetic variance per site, *v*_*S*_ = 2*w*^2^/*nN*, regardless of the selection coefficient (Section 3.1 in S1 Text; Fig 2A and Fig A1b in S1 Text). Henceforth, we measure genetic variance in units of *v*_*S*_. When selection is effectively neutral (roughly corresponding to *S* < 1) and thus too weak to affect allele frequency, the expected contribution of a site to genetic variance scales with the effect size and equals ½*S* (·*v*_*s*_) and therefore is lower than under strong selection (Section 3.1 in S1 Text; Fig 2A and Fig A1a in S1 Text). In between these selection regimes, selection effects on allele frequency are more complex and are influenced by underdominance (Section 3.1 in S1 Text). As the selection coefficient increases, the expected contribution to variance reaches *v*_*S*_ at *S* ≈ 3 and continues to increase until it reaches a maximal contribution that is approximately 30% greater at *S* ≈ 10 (Fig 2A), after which it slowly declines to the asymptotic value of *v*_*S*_ (Fig 2A and Fig A1b in S1 Text). Henceforth, we refer to this selection regime as intermediate (not to be confused with the nearly neutral range, which is much narrower and does not include selection coefficients with *S* > 10). These results suggest that effectively neutral sites should contribute much less to genetic variance than intermediate and strongly selected ones [67,68].

While intermediate and strongly selected sites contribute similarly to variance, their minor allele frequencies (MAFs) can differ markedly (Fig 2B). As an illustration, segregating sites with MAF > 0.1 account for approximately 72% and approximately 49% of the additive genetic variance for intermediate selection coefficients of *S* = 3 and 10, respectively, when almost no segregating sites would be found at such high MAF for a strong selection coefficient of *S* = 100 (Fig 2B). Thus, within the wide range of selection coefficients characterized as intermediate and strong, genetic variance arises from sites segregating at a wide range of MAFs ranging from common to exceedingly rare.

### Distribution of variances among sites

Next, we consider how genetic variance is distributed among sites with a given selection coefficient. We focus on the distribution among segregating sites (including monomorphic effects would just add a point mass at 0). This distribution is especially relevant to interpreting the results of GWASs, because, to a first approximation, a study will detect only sites with contributions to variance exceeding a certain threshold, $v(=2a_{1}^{2}pq)$, which decreases as the study size increases (see “Discussion”). We therefore depict the distribution in terms of the proportion of genetic variance, G(*v*), arising from sites whose contribution to genetic variance exceeds a threshold *v*.

**Fig 3:**
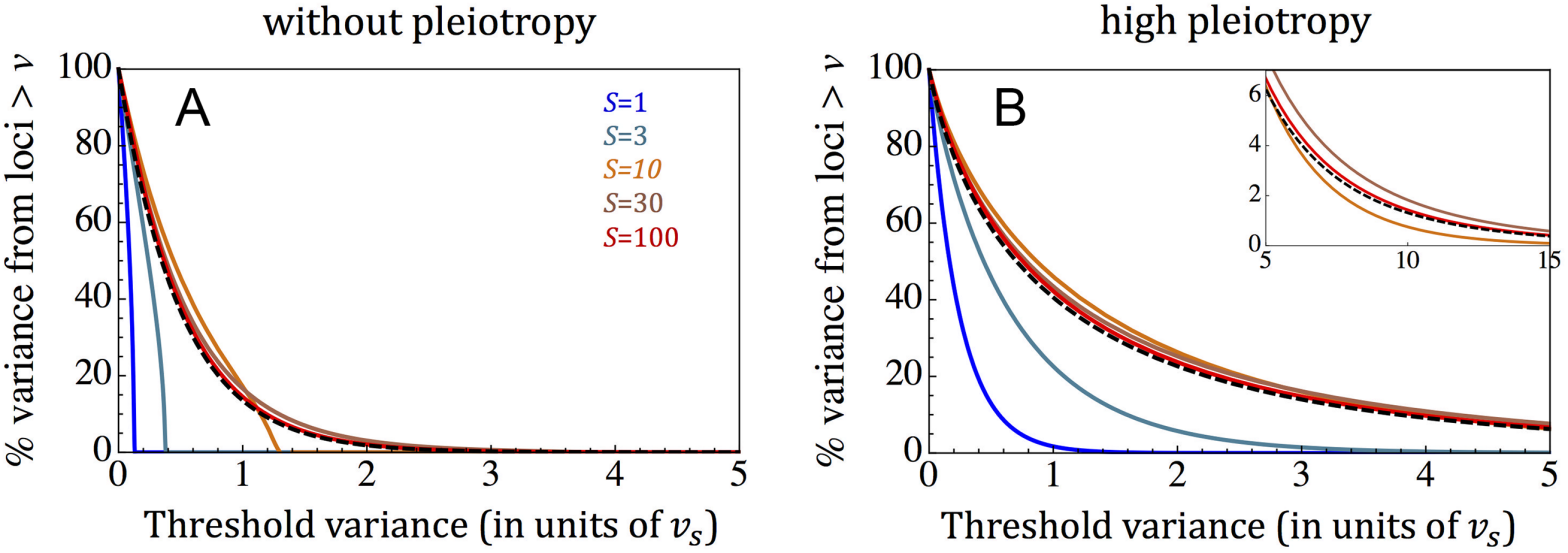
The proportion of additive genetic variance that arises from sites that contribute more than the value on the *x*-axis, for a single trait (A) and in the pleiotropic limit (B). Our approximations for sites under strong selection (Eqs 12 and 14) are shown with the dashed black curves. For the approximations in the effectively neutral limit (Eqs 14 and 16), see Fig A4 in S1 Text.

We begin with the case without pleiotropy (*n* = 1), in which selection on an allele determines its effect size (Fig 3A). When selection is strong (*S* > 30), the proportion of genetic variance exceeding a threshold *v* is also insensitive to the selection coefficient and takes a simple form, with

G(v)=exp(−2v) (13)

(Fig 3A; Section 3.2 in S1 Text). In contrast, in the effectively neutral range (*S* < 1),

$$\text{G}\left(v\right)=\sqrt{1-v/v_{max}},$$ (14)

where the dependency on the selection coefficient enters through $v_{max}=\frac{1}{8}S$, which is the maximal contribution to variance and corresponds to an allele frequency of ½ (Fig A4a; Section 3.2 in S1 Text). In the intermediate selection regime, G(*v*) is also intermediate and takes a more elaborate functional form (Section 3.2 in S1 Text). These results suggest how genetic variance would be distributed among sites given any distribution of selection coefficients (Fig 3A): starting from sites that contribute the most, the distribution would at first be dominated by strongly selected sites, and then the intermediate selected sites would begin to contribute, whereas effectively neutral sites would enter only for $v<\frac{1}{8}S\ll 1$.

Pleiotropy causes sites with a given selection coefficient to have a distribution of effect sizes on the focal trait, thereby increasing the contribution to genetic variance of some sites and decreasing it for others. In Section 3.2 of S1 Text, we show that increasing the degree of pleiotropy, *n*, increases the proportion of genetic variance, G(*v*), for any threshold, *v*, regardless of the distribution of selection coefficients (Fig A5 in S1 Text). When variation in a trait is sufficiently pleiotropic for the distribution of effect sizes to attain the limit form (Eq 11)

$$\text{G}\left(v\right)=\left(1+2\sqrt{v}\right)\;\text{exp}\left(-2\sqrt{v}\right)$$ (15)

for strongly selected sites and

G(v)=exp(−4v/S) (16)

for effectively neutral ones (Fig 3B and Fig A4b in S1 Text; Section 3.2 in S1 Text). The intermediate selection range is split between these behaviors: on the weaker end, roughly corresponding to *S* < 5, G(*v*) is similar to the effectively neutral case (Fig A4b and Section 3.2 in S1 Text); and on the stronger end, roughly corresponding to *S* > 5, G(*v*) is similar to the case of strong selection, with measurable differences only when *v* ≫ *v*_*s*_ (inset in Fig 3B and Section 3.2 in S1 Text). We would therefore expect that as the sample size of a GWAS increases and the threshold contribution to variance decreases, intermediate and strongly selected sites (more precisely, sites with *S* > 5) will be discovered first, and effectively neutral sites will be discovered much later. In S1 Text (Section 3.2 and Fig A3 in S1 Text), we also derive corollaries for the distribution of numbers of segregating sites that make a given contribution to genetic variance.

## Discussion

### Interpreting the results of human GWASs

In humans, GWASs for many traits display a similar behavior: when sample sizes are small, the studies discover almost nothing, but once they exceed a threshold sample size, both the number of associations discovered and the heritability explained begin to increase rapidly [4,77]. Intriguingly though, both the threshold study size and rate of increase vary among traits. These observations raise several questions: How is the threshold study size determined? How should the number of associations and explained heritability increase with study size once this threshold is exceeded? With an order of magnitude increase in study sizes into the millions imminent, how much more of the genetic variance in traits should we expect to explain? The theory that we developed provides tentative answers to these questions.

To relate the theory to GWASs, we must first account for the power to detect loci that contribute to quantitative genetic variation. In studies of continuous traits, the power can be approximated by a step function, where loci that contribute more than a threshold value v* to additive genetic variance will be detected and those that contribute less will not (Section 6.1 in S1 Text). The threshold depends on the study size, *m*, and on the total phenotypic variance in the trait, *V*_*P*_, where v* ∝ *V*_*P*_/*m* (Section 6.1 in S1 Text; [77]); conversely, the study size *m* needed to detect loci with contributions above v* is proportional to *V*_*P*_/v*. Given a trait and study size, the number of associations discovered and heritability explained then follow from our predictions for the distribution of variances among sites.

**Fig 4:**
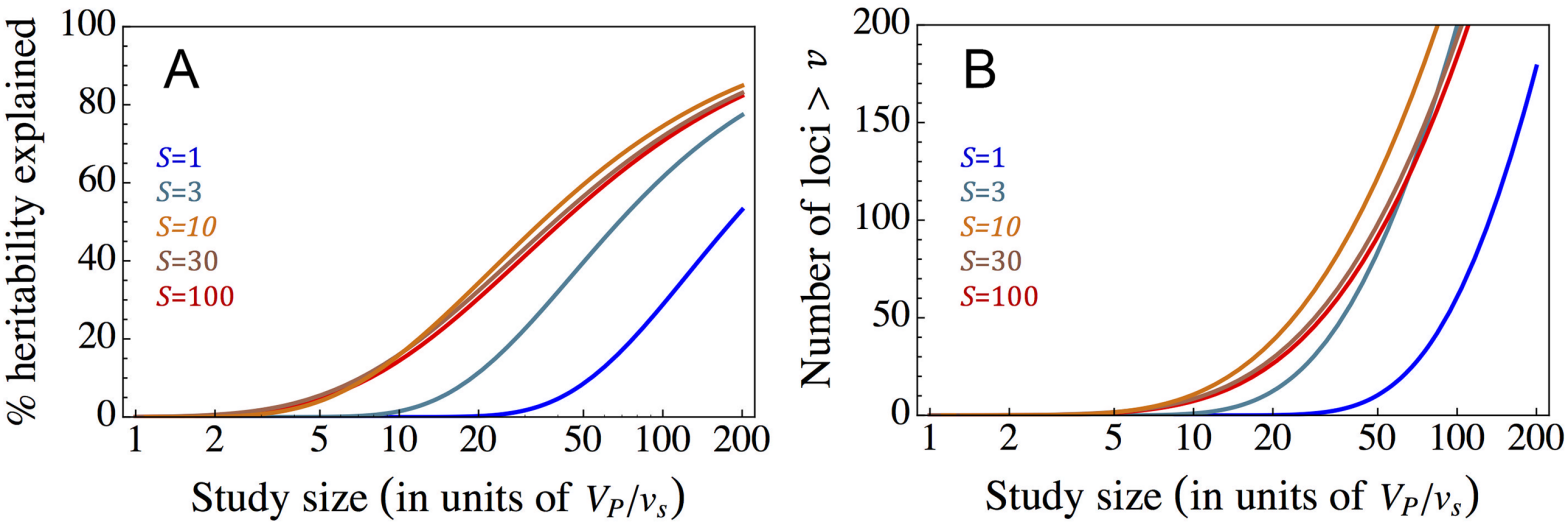
The proportion of heritability (A) and the number of variants (B) identified in a genome-wide association study (GWAS) as a function of study size. We assume the pleiotropic limit and a mutational target size of 1 Mb (see Sections 3.3 and 6.1 in S1 Text for derivations). For the case without pleiotropy, see Fig A22 in S1 Text.

When genetic variation in a trait is sufficiently pleiotropic, our results suggest that the first loci to be discovered in GWASs will be intermediate or strongly selected, with correspondingly large effect sizes (i.e., $S\approx \frac{2Nn}{w^{2}}a_{1}^{2}>5)$. The functional form of the distribution of variances among these loci (Eq 15 and Fig 3B) implies that for GWASs to capture a substantial proportion of the genetic variance, their threshold variance for detection v* has to be on the order of the expected variance contributed by strongly selected sites, *v*_*s*_, or smaller. We therefore expect the threshold study size for the discovery of intermediate and strongly selected loci to be proportional to *V*_*P*_/*v*_*s*_. When the study size exceeds this threshold, the number of associations detected and proportion of variance explained depend on the study size measured in units of *V*_*P*_/*v*_*s*_ (Fig 4) and follow from the functional forms that we derived (Eq 15 and Table A1 in S1 Text). The dependence on *V*_*P*_/*v*_*s*_ makes intuitive sense, as the total phenotypic variance *V*_*P*_ is background noise for the discovery of individual loci whose contributions to variance are on the order of *v*_*s*_. Some results are modified when variation in a trait is only weakly pleiotropic, which is probably less common: notably, the threshold study size for strongly selected loci would be higher, and loci under intermediate selection would begin to be discovered only after the strongly selected ones (Fig A22 in S1 Text, Eq 13, and Eq A35 in S1 Text). Regardless of the degree of pleiotropy, effectively neutral loci would only begin to be discovered at much larger study sizes, after the bulk of intermediate and strongly selected variance has been mapped (Fig 4 and Fig A22 in S1 Text). Thus, the dependence of the explained heritability on study size is largely determined by *V*_*P*_/*v*_*s*_ and by the proportion of heritable variance arising from intermediate and strongly selected loci, whereas the number of associations also depends on the mutational target size, providing a tentative explanation for why the performance of GWASs varies among traits.

### Inference and prediction

**Fig 5:**
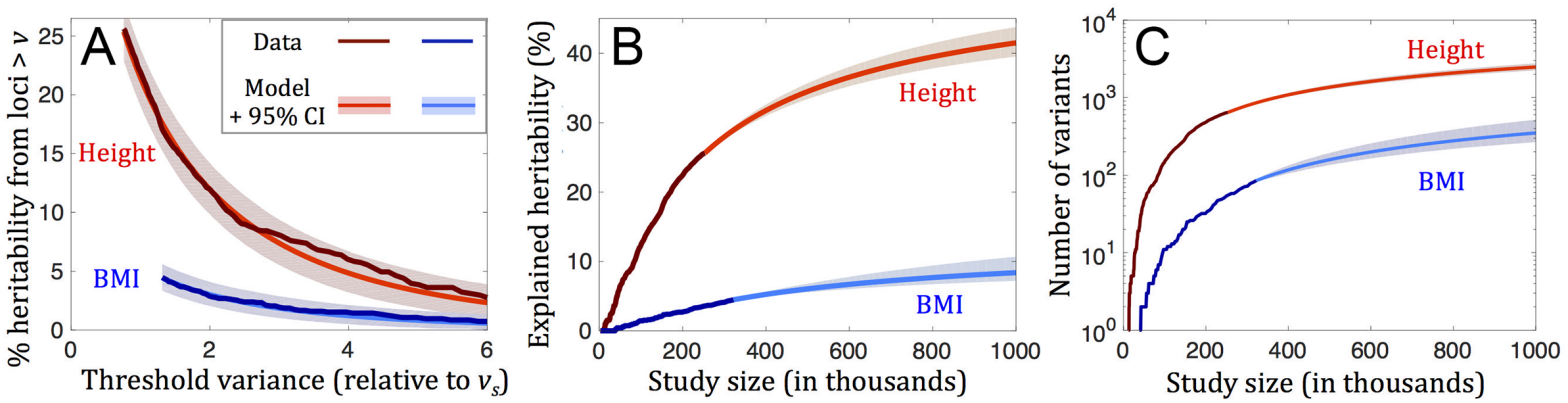
Model fit and predictions for height and body mass index (BMI), based on data from [16] and [15], respectively. In (A), we show the fit for associated loci. In (B) and (C), we show our predictions for future increases in the heritability explained and number of variants identified as genome-wide association study (GWAS) size increases. 95% CIs are based on bootstrap; see Section 7.4 in S1 Text for details.

Importantly, these theoretical predictions can be tested. As an illustration, we consider height and body mass index (BMI) in Europeans, 2 traits for which GWASs have discovered a sufficiently large number of genome-wide significant (GWS) associations (697 for height [16] and 97 for BMI [15]) for our test to be well powered. We fit our theoretical predictions to the distributions of variances among GWS associations reported for each of these traits, assuming that these distributions faithfully reflect what they would look like for the true causal loci (see Section 6.3 in S1 Text). We further assume that these loci are under intermediate or strong selection (as our predictions suggest) and that they are highly pleiotropic (see "Introduction"; [37, 42, 45]). Under these assumptions, we expect the distribution of variances to be well approximated by a simple form (Eq A89 in S1 Text), which depends on a single parameter, *v*_*s*_. We find that the theoretical distribution with the estimated *v*_*s*_ fits the data for both traits well (Fig 5A): we cannot reject our model based on the data for either trait (by a Kolmogorov-Smirnov test, *p* = 0.14 for height and *p* = 0.54 for BMI; Section 7.5 in S1 Text). By comparison, without pleiotropy (*n* = 1), our predictions provide a poor fit to these data (by a Kolmogorov-Smirnov test, *p* < 10^−5^ for height and *p* = 0.05 for BMI; Fig A14 in S1 Text).

Fitting the model to GWAS results further allows us to make inferences about evolutionary parameters (Sections 7.1 and 7.3 in S1 Text). By including the degree of pleiotropy (*n*) as an additional parameter, we find that for both height and BMI, *n* is sufficiently large for it to be indistinguishable from the high pleiotropy limit. Based on the shape of the distributions in this limit and on scaling the threshold values of v* in units of our estimates for *v*_*s*_, we estimate that the proportion of variance arising from mutations within the range of detectable selection effects is approximately 50% for height and approximately 15% for BMI. Further relying on the number of associations that fall above the thresholds, we infer that, within this range, height has a mutational target size of approximately 5 Mb, whereas BMI has a target size of approximately 1 Mb (Table A2 in S1 Text).

These parameter estimates can help to interpret GWAS results. They suggest that, despite their comparable sample sizes, the GWAS for height succeeded in mapping a substantially greater proportion of the heritable variance than the GWAS for BMI (approximately 20% compared to approximately 3%–5%) primarily because the proportion of variance arising from mutations within the range of detectable selection effects for height is much greater than for BMI. Moreover, the estimates of target sizes and the relationship between sample size and threshold contribution to variance can be used to predict how the explained heritability and number of associations should increase with sample size (Fig 5B and 5C). These predictions are likely underestimates as the range of detectable selection effects itself should also increase with sample size.

We can also examine to what extent our inferences are consistent with data and estimates from earlier studies. For example, the distribution of variances that we inferred for height fits those obtained in a recent GWAS of height based on exome genotyping (Kolmogorov-Smirnov test, *p* = 0.99; Fig A15b and Section 8.1 in S1 Text). In addition, the proportion of variance that we estimate to arise from the range of selection effects detectable in existing GWASs for height and BMI is consistent with estimates of the heritable variance tagged by all single-nucleotide polymorphisms (SNPs) with MAF > 1% [60, 61]; Section 8.2 in S1 Text.

### The effect of polygenic adaptation

While we have assumed that quantitative traits have been subject to long-term stabilizing selection, recent studies indicate that some traits, and height in particular, have also been subject to recent directional selection [78–82]. Under plausible evolutionary scenarios, recent directional selection can induce large changes to the mean phenotype through the collective response at many segregating loci while having a negligible effect on allele frequencies at individual loci [21,83]. This very subtle effect on allele frequencies is likely one reason why polygenic adaptation is so difficult to detect and why studies have to pool faint signals across many loci to do so [78–82]. In Section 5.1 of S1 Text, we show that the distribution of allele frequencies on which our results rely is insensitive to sizable recent changes to the optimal phenotype. Importantly then, even when recent directional selection has occurred and its effects are discernable, the genetic architecture of a trait is nonetheless likely to be dominated by the effects of longer-term stabilizing selection.

### The effect of demography

In contrast, recent changes in the effective population size are likely to have had a dramatic effect on allele frequencies and thus on the genetic architecture of quantitative traits [84,85]. In particular, European populations in which the GWASs for height and BMI were performed are known to have experienced dramatic changes in population size, including an Out-of-Africa (OoA) bottleneck about 100 KYA and explosive growth over the past 5 KY [86–89]. To study how these changes would have affected genetic architecture, we simulated allelic trajectories under our model and historical changes in population sizes in Europeans (relying on the model of [89]; Section 9 in S1 Text).

**Fig 6:**
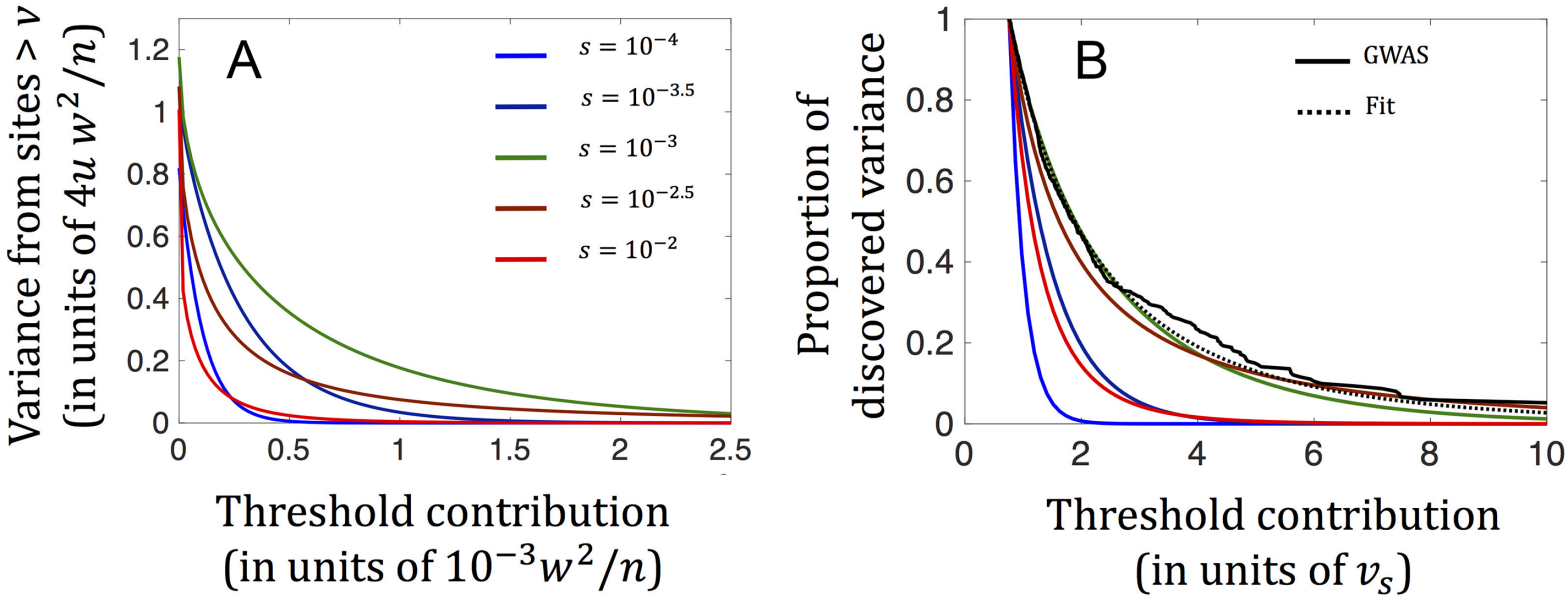
The combined effect of selection and changes in population size (as inferred by [89] for Europeans) on the distribution of variances among segregating sites. (A) The cumulative variance arising from sites with contributions above a threshold as a function of the threshold, for different selection coefficients. Cumulative variance is measured in units of 4*u* · *w*^2^/*n*, the equilibrium expectation for a strongly selected site, while the threshold is in units of 10^−3^ · *w*^2^/*n*. (B) The distribution of variances among loci identified in the genome-wide association study (GWAS) of height. The empirical distribution is in solid black, and our inferred fit is in dashed black. Simulation results for each selection coefficient (in color) are normalized such that the proportion of variance at the study threshold is always 1. For similar results corresponding to BMI, see Fig A20b in S1 Text, and for further details, see Section 9 in S1 Text.

Our results suggest that the individual segregating sites with the greatest contribution to the extant genetic variance have selection coefficients around *s* = 10^−3^ and are due to mutations that originated shortly before or during the OoA bottleneck (Fig 6A and Section 9 in S1 Text). These mutations ascended to relatively high frequencies during the bottleneck and minimally decreased in frequency during subsequent, recent increases in population size, thereby resulting in large contributions to current genetic variance. Segregating sites under weaker selection contribute much less to variance because of their smaller effect sizes (i.e., for the same reason that applied in the case with a constant population size). Finally, and in contrast to the case with a constant population size, individual segregating sites under stronger selection (e.g., *s* ≥ 10^−2.5^) contribute much less to current variance than those with *s* ≈ 10^−3^. Mutations at these sites are younger and arose after the bottleneck, when the population size was considerably larger, resulting in much lower initial and current frequencies and therefore a lower per (segregating) site contribution to variance (as distinct from the proportion of strongly selected sites that are currently segregating, which will have greatly increased, resulting in the same total contribution to variance; [84, 85]). In Section 10 in S1 Text, we discuss one implication of these demographic effects: that the reliance on genotyping rather than resequencing in GWASs had a minimal effect on the discovery of associations.

Segregating loci with *s* ≈ 10^−3^ not only make the largest contributions to the current variance but also are likely to account for most of the GWS associations in the GWASs of height and BMI (Section 9 in S1 Text). When we account for the discovery thresholds of these studies, the expected distribution of variances for loci with *s* ≈ 10^−3^ closely matches the distribution observed among GWS associations (Fig 6B and Fig A20b in S1 Text). Moreover, these distributions closely match our theoretical predictions for *s* ≈ 10^−3^ and an *N*_*e*_ ≈ 5,000 (Fig 6B)—roughly the effective population size experienced by mutations that originated shortly before or during the bottleneck. This match likely explains why the results predicted on a constant population size fit the data well nonetheless. Our interpretation of GWAS findings is supported by other aspects of the data (Section 9 in S1 Text).

Our conclusions about the high degree of pleiotropy of genetic variation for height and BMI and the differences between these traits are likely robust to demographic effects, given how well our model fits the distributions of variances among loci, once we account for European demographic history. However, we might be underestimating the mutational target sizes and total heritable variances associated with the selection effects currently visible in GWASs, as simulations with European demographic history indicate that the proportion of variance arising from loci with *s* ≈ 10^−3^ explained by current GWASs is lower than our equilibrium estimates (approximately 42% compared to approximately 53% for height and approximately 29% compared to approximately 38% for BMI). By the same token, we likely underestimated the future increases in explained heritability with increases in study sizes (Fig 5B and 5C).

### Conclusion

In summary, a ground-up model of stabilizing selection and pleiotropy can go a long way toward explaining the findings emerging from GWASs. Important next steps involve explicitly using more information from GWASs in the inferences. In particular, we can learn more about the selection acting on quantitative genetic variation by explicitly incorporating information about frequency and effect size (rather than their combination in terms of variance) and by including information from associations that do not attain genome-wide significance. Doing so will further require directly incorporating the effects of recent demographic history on genetic architecture [84,85]. An extended version of the inference, applied to the myriad traits now subject to GWASs, should allow us to learn about differences in the genetic architectures of traits and answer long-standing questions about the evolutionary forces that shape quantitative genetic variation.

## Supporting information

S1 Text Supplementary information. (PDF)

## Abbreviations

BMI: body mass index
GWAS: genome-wide association study
GWS: genome-wide significant
MAF: minor allele frequency
OoA: Out of Africa
SNP: single-nucleotide polymorphism
