# Supplementary material for: A population genetic interpretation of GWAS findings for human quantitative traits
Source: PLoS Biol. 2018 Mar 16;16(3):e2002985. doi: 10.1371/journal.pbio.2002985 (PMC5871013; doi:10.1371/journal.pbio.2002985)
Supplement: S1 Text — (PDF) [file pbio.2002985.s001.pdf]

A population genetic interpretation of GWAS findings for human quantitative traits

Supplementary Information

Yuval B. Simons[a,*], Kevin Bullaughey[b], Richard R. Hudson[b], and Guy Sella[a,*]

[a] Department of Biological Sciences, Columbia University, New York, NY 10027;

[b] Department of Ecology & Evolution, University of Chicago, Chicago, Illinois 60637;

[*] To whom correspondence should be addressed: yuval.simons@columbia.edu, gs2747@columbia.edu

Table of Contents

1. The model ... 3
1.1. Absorbing the environmental contribution into the fitness function ... 3
1.2. The distribution of mutational effect sizes on a given trait ... 3
2. Solving for summaries of genetic architecture ... 5
2.1. The first two moments of change in allele frequency ... 5
2.2. Sojourn time ... 6
2.3. Calculating expectations of summaries of architecture ... 6
3. Additive genetic variance and number of segregating sites ... 8
3.1. Expectations ... 8
3.2. Densities ... 10
3.3. Comparing predictions against simulations ... 14
4. Justification for assumptions ... 17
4.1. Normal and isotropic phenotypic distribution around the optimum ... 17
4.2. The phenotypic variance satisfies $\sigma^2 \ll w^2$ ... 17
4.3. Mutational effect sizes satisfy $a^2 \ll w^2$ ... 18
4.4. Deviations of the mean phenotype from the optimum can be neglected ... 18
5. Model robustness ... 20
5.1. Changes to the optimal phenotype ... 20
5.2. Asymmetric mutational input ... 22
5.3. Major effect loci ... 26

5.4. Anisotropic mutation ... 27
6. The power to detect loci in GWAS ... 33
6.1. Re-sequencing studies ... 33
6.2. Genotyping ... 35
6.3. Tagging ... 36
7. Inference ... 38
7.1. The composite likelihood ... 38
7.2. Determining v^* and removing outliers ... 39
7.3. Estimating target size and explained variance ... 40
7.4. Estimating confidence intervals ... 41
7.5. Testing goodness of fit ... 42
8. Consistency with other datasets and analyses ... 44
8.1. Exome association study of height ... 44
8.2. The heritability arising from common SNPs ... 46
8.3. The relationship between SNP heterozygosity and effect size ... 48
9. The effects of demographic history ... 50
10. The effects of genotyping ... 57
11. Glossary of notation ... 59
12. Additional figure ... 60
13. Bibliography ... 61

1. The model

1.1. Absorbing the environmental contribution into the fitness function

Here, we show that the additive environmental contribution to the phenotype can be absorbed into the fitness function, which justifies considering only the additive genetic contribution in our analysis. This result has been derived multiple times for the one dimensional case (e.g., 1). The argument in the multi-dimensional case is similar and included for completeness.

First, assume that the additive environmental contribution to the phenotype, $\vec{r}_e$, is distributed as a multi-normal with mean 0 and isotropic variance V_e. The expected absolute fitness of an individual with additive genetic contribution to the phenotype, $\vec{r}_g$, is given by averaging fitness over the distribution of environmental contributions. Namely,

$$\overline{\mathrm{W}}(\vec{r}_g) = \int_{\vec{r}_e} \frac{1}{(2\pi V_e)^{n/2}} e^{-\frac{\|\vec{r}_e\|^2}{2V_e}} \mathrm{W}(\vec{r}_g + \vec{r}_e) = \int_{\vec{r}_e} \frac{1}{(2\pi V_e)^{n/2}} e^{-\frac{\|\vec{r}_e\|^2}{2V_e}} e^{-\frac{\|\vec{r}_g+\vec{r}_e\|^2}{2w^2}}$$

$$= \frac{1}{(1+V_e/w^2)^{n/2}} e^{-\frac{\|\vec{r}_g\|^2}{2(w^2+V_e)}}. \qquad \text{(A1)}$$

Given that absolute fitness is defined up to a multiplicative constant, we can therefore absorb the additive environmental contribution by using the Gaussian fitness function

$$\widetilde{\mathrm{W}}(\vec{r}_g) = \exp\left(-\frac{\|\vec{r}_g\|^2}{2\widetilde{w}^2}\right), \qquad \text{(A2)}$$

where $\widetilde{w}^2 = w^2 + V_e$. Even when the environmental contribution is anisotropic, we can always choose a coordinate system in which the effective fitness function takes an isotropic form around the fitness peak (Eq. 1, which appears in the Model section of the main text).

1.2. The distribution of mutational effect sizes on a given trait

In the main text, we define the distribution of phenotypic effects of newly arising mutations in the n-dimensional trait space, $\vec{a}$. Here, we consider the projection of these effects on a given trait, a_1, taken without loss of generality to be on the 1st dimension. The distribution of effect sizes on a focal trait will depend on the degree of pleiotropy, n, and the form of this dependency becomes important when we consider how pleiotropy affects genetic architecture.

We want to calculate the distribution of effect sizes on the focal trait, a_1, conditional on their overall effect, $a = \|\vec{a}\|$. We assume that the distribution of effects of de novo

mutations is isotropic in trait space. The effect of a mutation, $\vec{a}$, therefore has equal probability to occupy any point on an n-dimensional sphere with radius a. Let $\mathrm{S}_m(x)$ denote the surface area of an m-dimensional sphere of radius x and θ denote the angle between the vector $\vec{a}$ and its projection a_1, i.e., $a_1 = a\cos\theta$. In these terms, the surface area element corresponding to angle θ is

$$\mathrm{S}_{n-1}(a\sin\theta)\; a\; d\theta, \tag{A3}$$

and by a change of variables, the surface area element corresponding to projection a_1 on the focal trait is

$$\mathrm{S}_{n-1}(a\sin\theta)\; a\; d\theta = \mathrm{S}_{n-1}\left(\sqrt{a^2-a_1^2}\right)\frac{a}{\sqrt{a^2-a_1^2}}da_1, \tag{A4}$$

since $da_1 = \left|\frac{da_1}{d\theta}\right| d\theta = a\sin\theta\, d\theta = \sqrt{a^2-a_1^2}d\theta$. This result implies that the probability density of a_1 is

$$\varphi_n(a_1|a) = \frac{\mathrm{S}_{n-1}\left(\sqrt{a^2-a_1^2}\right)}{\mathrm{S}_n(a)}\frac{a}{\sqrt{a^2-a_1^2}} = \frac{\Gamma\left(\frac{n}{2}\right)}{\sqrt{\pi}\,\Gamma\left(\frac{n-1}{2}\right)}\left(1-\frac{a_1^2}{a^2}\right)^{\frac{n-3}{2}}\frac{1}{a} \tag{A5}$$

(for a similar derivation, see (2)).

Next, we consider the high pleiotropy limit form of this distribution. For any degree of pleiotropy, the symmetry of the mutational distribution implies that

$$\mathrm{E}(a_1|a) = 0 \tag{A6}$$

and the equivalence among traits implies that

$$\mathrm{V}(a_1|a) = a^2/n \tag{A7}$$

(see main text for more details). It follows that when n becomes sufficiently large, $a_1/a \ll 1$ and therefore

$$\left(1-\frac{a_1^2}{a^2}\right)^{\frac{n-3}{2}} \approx \exp\left(-\frac{n}{2}\;\frac{a_1^2}{a^2}\right). \tag{A8}$$

In addition, $\Gamma\left(\frac{n}{2}\right)/\Gamma\left(\frac{n-1}{2}\right) \approx \sqrt{n/2}$. Substituting these expressions into Eq. A5, we find that for sufficiently large n the distribution of effect sizes approaches the normal distribution

$$\varphi_n(a_1|a) \approx \frac{1}{\sqrt{2\pi(a^2/n)}}\exp\left(-\frac{1}{2}\;\frac{a_1^2}{a^2/n}\right). \tag{A9}$$

As we elaborate in the main text, important implications about quantitative genetic variation follow from this high pleiotropy limit. The limit also holds quite generally when the distribution of effect sizes is anisotropic (see Section 5.4).

2. Solving for summaries of genetic architecture

Here, we derive closed forms for summaries of genetic architecture under our model. We begin by deriving the first two moments of change in allele frequency in a single generation. With these moments at hand, we use the diffusion approximation to calculate the sojourn time for alleles that contribute to quantitative genetic variation (3). Together with the distribution of effect sizes derived in the previous section, the sojourn time allows us to obtain closed forms for summaries of genetic architecture. Specifically, we can obtain a closed form for any summary that can be described as a function of allele frequencies and effect sizes at sites contributing to quantitative genetic variation. We use these expressions to calculate the summaries used in the main text, for example the expected additive genetic variance and its distribution across sites.

2.1. The first two moments of change in allele frequency

We assume that:

- The phenotypic distribution at steady state is well approximated by an isotropic multivariate normal distribution centered at the optimum, namely by the probability density
  $$f(\vec{r}) = \frac{1}{(2\pi\sigma^2)^{n/2}} exp\left(-\frac{r^2}{2\sigma^2}\right). \quad \text{(A10)}$$
- Both a^2 and $\sigma^2 \ll w^2$.

These assumptions are justified in Section 3.3.

We rely on these assumptions to calculate the first two moments of change in frequency in a single generation for an allele with phenotypic effect $\vec{a}$ and frequency q. The fitnesses of the three genotypes at the site depend on its distribution of genetic backgrounds, i.e., on the total phenotypic contribution of sites other than the focal one, $\vec{R}$. Following Eq. A10 and assuming every allele contributes only a small proportion of the genetic variance, the distribution of $\vec{R}$ is well approximated by

$$\mathrm{f}(\vec{R}|\vec{a}, q) = \frac{1}{(2\pi\sigma^2)^{n/2}} \exp\left(-\frac{\|\vec{R}+2q\vec{a}\|^2}{2\sigma^2}\right). \quad \text{(A11)}$$

The expected fitnesses of the three genotypes then follow from integrating over backgrounds:

$$W_{00} = \int_{\vec{R}} \mathrm{f}(\vec{R}|\vec{a}, q)\mathrm{W}(\vec{R}) = \left(\frac{w}{\sqrt{w^2+\sigma^2}}\right)^n \exp\left(-\frac{4a^2q^2}{2(w^2+\sigma^2)}\right), \quad \text{(A12)}$$

$$W_{01} = \int_{\vec{R}} \mathrm{f}(\vec{R}|\vec{a}, q)\mathrm{W}(\vec{R}+\vec{a}) = \left(\frac{w}{\sqrt{w^2+\sigma^2}}\right)^n \exp\left(-\frac{4a^2(q-1/2)^2}{2(w^2+\sigma^2)}\right) \quad \text{(A13)}$$

and

$$W_{11} = \int_{\vec{R}} \mathrm{f}(\vec{R}|\vec{a}, q)\mathrm{W}(\vec{R}+2\vec{a}) = \left(\frac{w}{\sqrt{w^2+\sigma^2}}\right)^n \exp\left(-\frac{4a^2(q-1)^2}{2(w^2+\sigma^2)}\right). \quad \text{(A14)}$$

The first moment of change in allele frequency is then

$$\mathrm{E}(\Delta q) = -pq\frac{p(W_{00}-W_{01})+\mathrm{q}(W_{01}-W_{11})}{\bar{W}} \approx -\frac{a^2}{w^2}pq\left(\frac{1}{2}-q\right), \quad \text{(A15)}$$

relying on our assumptions that a^2 and $\sigma^2 \ll w^2$. The functional form of the first moment is equivalent to that of the standard viability selection model with under-dominance and selection coefficient $s = \frac{a^2}{w^2}$ or scaled selection coefficient

$$S = 2N\frac{a^2}{w^2}. \quad \text{(A16)}$$

Similarly, we find that

$$\mathrm{V}(\Delta q) \approx \frac{pq}{2N}, \quad \text{(A17)}$$

which is the standard second moment with genetic drift.

2.2. Sojourn time

Based on the first two moments, we can use the diffusion approximation to calculate the sojourn time as a function of allele frequency, i.e., the density of the time that an allele spends at a given frequency q before it fixes or is lost (3). For a mutant allele with initial frequency $1/2N$ and scaled selection coefficient S, the sojourn time is

$$\tau(q|S) = \begin{cases} \frac{N\sqrt{\pi/S}}{\mathrm{erf}(\sqrt{S}/2)} \frac{e^{S(1-2x)^2/4}}{x(1-x)} \mathrm{f}_-(S,q)\mathrm{f}_+(S,1/2N) & 0 \le q \le 1/2N \\ \frac{N\sqrt{\pi/S}}{\mathrm{erf}(\sqrt{S}/2)} \frac{e^{S(1-2x)^2/4}}{x(1-x)} \mathrm{f}_+(S,q)\mathrm{f}_-(S,1/2N) & 1/2N \le q \le 1 \end{cases} \quad \text{(A18)}$$

where erf is the error function and $\mathrm{f}_\pm(S,y) \equiv \mathrm{erf}(\sqrt{S}/2) \pm \mathrm{erf}(\sqrt{S}(1-2y)/2)$.

The sojourn time takes simple limiting forms when selection is effectively neutral ($S \ll 1$) or strong ($S \gg 1$). In the effectively neutral range, it is well approximated by $\tau(q|S) = 2/\mathrm{q}$, and in the strongly selected range, it is well approximated by $\tau(q|S) = 2\exp(-Sq)/q$.

2.3. Calculating expectations of summaries of architecture

Many summaries of interest can be expressed as sums over segregating sites of some function $c(q, a_1)$, where q is the derived allele frequency and a_1 is the effect size on the

trait. For example, the additive genetic variance in a trait is given by the sum of $v(q, a_1) = \ 2a_1^2 q(1-q)$ over sites. The expectation over such summaries can be expressed as

$$\mathrm{E}(C) = 2NU \int_q \int_{a_1} c(q, a_1)\rho(q, a_1)\ , \qquad \text{(A19)}$$

where C is the summery summed over all sites, $2NU$ is the population mutation rate per generation and $\rho(q, a_1)$ is the density of sites with the corresponding frequency and effect size per unit mutational input.

The density $\rho(q, a_1)$ can be broken down into contributions from sites with different selection coefficients, i.e.,

$$\rho(q, a_1) = \int_S \mathrm{f}(S)\big(\tau(q|S)\eta(a_1|S)\big), \qquad \text{(A20)}$$

where $\mathrm{f}(S)$ is the distribution of selection coefficients and $\tau(q|S)$ is the sojourn time of a mutation with selection coefficient S (Eq. A18). The probability density $\eta(a_1|S)$ of effect sizes given selection coefficient S follows from Eqs. A5 and A16

$$\eta(a_1|S) = \varphi_{\mathrm{n}}(a_1|a(S)) = \varphi_{\mathrm{n}}\left(a_1\middle|\sqrt{(w^2/2N)S}\right). \qquad \text{(A21)}$$

This allows us to break down our summaries into contributions from sites with different selection coefficients

$$\mathrm{E}(C) = 2NU \int_S \mathrm{f}(S)\mathrm{E}(C|S) \qquad \text{(A22)}$$

with

$$\mathrm{E}(C|S) = \int_q \int_{a_1} c(q, a_1)\tau(q|S)\eta(a_1|S). \qquad \text{(A23)}$$

We use Eq. A23 to study how summaries of architecture depend on the strengths of selection, and how these summaries will depend on different distributions of selection coefficients. This allows us to draw general implications about genetic architecture despite our limited knowledge about this distribution.

3. Additive genetic variance and number of segregating sites

The distributions of additive genetic variance and of the number of segregating sites are critical to understanding genetic architecture and specifically to interpreting results of GWAS. Here we derive closed forms for both distributions as well as simple approximations under strong and effectively neutral selection.

3.1. Expectations

We begin by considering the expected contribution of a site to additive genetic variance. Substituting the contribution to variance from a single site $v(q, a_1) = 2a_1^2 q(1-q)$ into Eq. A23, we find that

$$\mathrm{E}(V|S) = \int_q \int_{a_1} v(q, a_1)\tau(q|S)\eta(a_1|S) = \int_q 2q(1-q)\tau(q|S) \int_{a_1} a_1^2 \eta(a_1|S)$$

$$= \frac{2w^2}{nN} \int_q \frac{1}{2} Sq(1-q)\tau(q|S)\,. \qquad \text{(A24)}$$

The total additive genetic variance over all sites is

$$\sigma^2 = 2NU \int_S \mathrm{f}(S)\mathrm{E}(V|S). \qquad \text{(A25)}$$

The closed form for $\mathrm{E}(V|S)$ in Eq. A24 was integrated numerically to obtain Fig. 2a in the main text. We can use the results of Keightley and Hill (4) to obtain an analytic approximation for $\mathrm{E}(V|S)$:

$$\mathrm{E}(V|S) = \frac{2w^2}{nN} \sqrt{\frac{\pi S}{4}}\, \mathrm{erfi}\left(\sqrt{S}/4\right) \exp(-S/4) + O\left(\frac{1}{2N}\right), \qquad \text{(A26)}$$

where erfi is the imaginary error function ($\mathrm{erfi}(x) \equiv \mathrm{erf}(ix)/i$).

In the effectively neutral and strong selection limits, we can use limit forms of the sojourn time to derive simple approximations for $E(V|S)$. In the effectively neutral limit, i.e., when $S \ll 1$, $\tau(q|S) \approx 2/q$ and therefore

$$\mathrm{E}(V|S) \approx \frac{2w^2}{nN} \frac{S}{2}. \qquad \text{(A27)}$$

In practice, this expression provides a decent approximation when $S < 1$ (Fig. A1a). In the strong selection limit, when $S \gg 1$, $\tau(q|S) \approx 2\exp(-Sq)/q$ and therefore

$$\mathrm{E}(V|S) \approx \frac{2w^2}{nN}. \qquad \text{(A28)}$$

In practice, this expression provides a decent approximation when $S > 30$ (Fig. A1a). The constant $2w^2/nN$, which recurs in our derivations (e.g., Eq. A24), thus has a simple interpretation: it is the expected contribution of strongly selected sites to additive genetic

variance (per unit mutational input). We therefore denote it by v_s, and henceforth measure variance in these units.

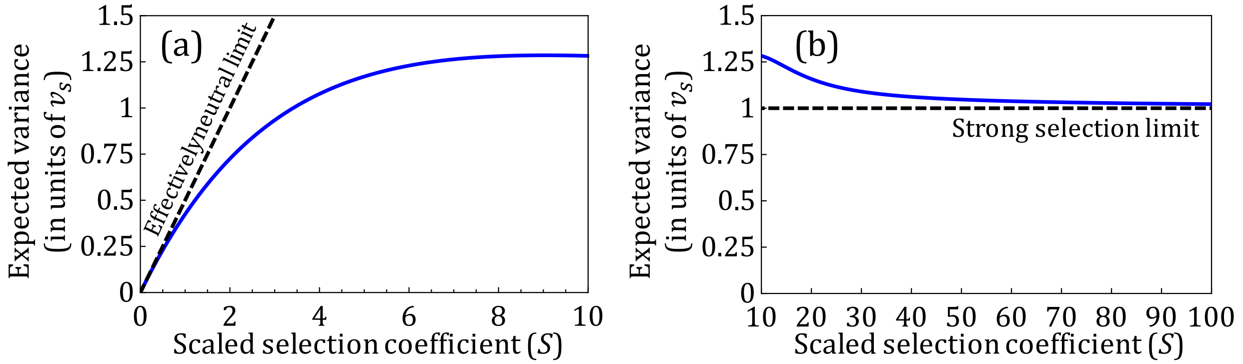

Figure A1. The effectively neutral and strong selection approximations for the expected contribution to genetic variance per site. (A) The expression in the limit of $S \ll 1$ provides a decent approximation when $S < 1$. (B) The expression in the limit of $S \gg 1$ provides a decent approximation when $S > 30$.

We next consider how the expected number of segregating sites depends on the strength of selection. This expectation (per unit mutational input) is simply the mean sojourn time of a newly arising mutation. Formally, it follows from substituting $\mathrm{k}(q, a_1) = 1$ into Eq. A23, i.e.,

$$\mathrm{E}(K|S) = \int_q \int_{a_1} \tau(q|S)\eta(a_1|S) = \int_q \tau(q|S) \int_{a_1} \eta(a_1|S) = \int_q \tau(q|S). \qquad \text{(A29)}$$

In Fig. A2, we calculate this integral numerically for different values of S, to find that the number of segregating sites depends only weakly on S. Intuitively, this follows from the fact that the vast majority of mutations, be they effectively neutral, intermediate, or strongly selected, spend only a few generations in the population at low copy numbers before going extinct.

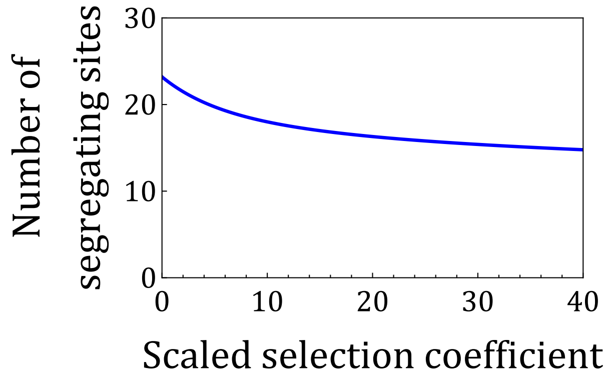

Figure A2. The number of segregating sites per unit mutational input (or, equivalently, the expected sojourn time of a newly arising mutation), Eq. A29, is only weakly dependent on the strength of selection. Calculated for a population size of 20,000.

3.2. Densities

Here, we consider how the additive genetic variance is distributed among sites. We begin by deriving a closed form for the density of segregating sites with a given contribution to variance v. This density follows from substituting Dirac's delta function $\delta\left(v-2a_1^2q(1-q)\right)$ into Eq. A23:

$$\rho(v|S) = \mathrm{E}\left(\delta\left(v-2a_1^2q(1-q)\right)|S\right) = \int_q \int_{a_1} \delta\left(v-2a_1^2q(1-q)\right)\tau(q|S)\eta(a_1|S) =$$

$$= \int_{a_1}\left(\tau(\mathrm{q}_+(v,a_1)|S)\left|\frac{\partial \mathrm{q}_+(v,a_1)}{\partial v}\right| + \tau(\mathrm{q}_-(v,a_1)|S)\left|\frac{\partial \mathrm{q}_-(v,a_1)}{\partial v}\right|\right)\eta(a_1|S)$$

$$= \int_{a_1}\left(\tau(\mathrm{q}_+(v,a_1)|S) + \tau(\mathrm{q}_-(v,a_1)|S)\right)\frac{1}{2a_1^2\sqrt{1-2v/a_1^2}}\,\eta(a_1|S), \qquad \text{(A30)}$$

where $\mathrm{q}_\pm(v,a_1) = \frac{1}{2}\left(1 \pm \sqrt{1-2v/a_1^2}\right)$ are the two frequencies for which $v = 2a_1^2q(1-q)$. This integral can be calculated numerically for any S and degree of pleiotropy n (by using the corresponding density $\eta(a_1|S)$). Moreover, as we illustrate below, summary statistics of the distribution of variances among sites can be expressed and calculated in terms of integrals over the density $\rho(v|S)$.

We can greatly simplify the expression for $\rho(v|S)$ in the limits of effectively neutral and strong selection, and especially in the cases without pleiotropy or with extensive pleiotropy. When selection is effectively neutral ($S \ll 1$), then $\tau(q|S) \cong 2/q$ and thus

$$\rho(v|S) = \int_{a_1}\left(\frac{2}{\mathrm{q}_+(v,a_1)} + \frac{2}{\mathrm{q}_-(v,a_1)}\right)\frac{1}{2a_1^2\sqrt{1-2v/a_1^2}}\,\eta(a_1|S)$$

$$= \int_{a_1}\left(\frac{4}{1+\sqrt{1-2v/a_1^2}} + \frac{4}{1-\sqrt{1-2v/a_1^2}}\right)\frac{1}{2a_1^2\sqrt{1-2v/a_1^2}}\,\eta(a_1|S) = \int_{a_1}\frac{2}{v\sqrt{1-2v/a_1^2}}\,\eta(a_1|\mathrm{S}), \qquad \text{(A31)}$$

with variance measured in units of v_s and effect size measured in units of $\sqrt{v_s}$. Without pleiotropy ($n=1$), the effect size is $a_1 = \pm\frac{1}{2}\sqrt{S}$ and the expression for the density simplifies to

$$\rho(v|S) = \frac{2}{v\sqrt{1-v/v_{max}}}, \qquad \text{(A32)}$$

where $v_{max} \equiv S/8$ is the maximal contribution to variance for a mutation with selection coefficient S, which is obtained when both alleles have frequency ½. When the degree of pleiotropy is high ($n \gg 1$), a_1 is approximately normally distributed with mean 0 and variance $S/4$ (Eq. 11, which appears in the Results section of the main text) and the expression for the density simplifies to

$$\rho(v|S) = \int_{a_1>\sqrt{2v}} \frac{2}{v\sqrt{1-2v/a_1^2}} \frac{2}{\sqrt{2\pi S/4}} \exp(-2a_1^2/S) = 2\exp(-4v/S)/v. \tag{A33}$$

When selection is strong, derived alleles are rare ($q \ll 1$), implying that $v \ll a_1^2$ and $q \approx v/2a_1^2$, and that the sojourn time is well approximated by $\tau(q|S) = 2\exp(-Sq)/q$. The density $\rho(v|S)$ then simplifies to

$$\rho(v|S) \approx \int_{a_1} \tau(q(v,a_1)|S) \frac{1}{2a_1^2} \eta(a_1|S) \approx \int_{a_1} \frac{4a_1^2}{v} \exp(-Sv/2a_1^2) \frac{1}{2a_1^2} \eta(a_1|S)$$

$$= \int_{a_1} \frac{2}{v} \exp(-Sv/2a_1^2)\, \eta(a_1|S). \tag{A34}$$

Without pleiotropy, this expression further simplifies to

$$\rho(v|S) \approx 2\exp(-2v)/v, \tag{A35}$$

and when the degree of pleiotropy is high ($n \gg 1$), then

$$\rho(v|S) \approx \int_{a_1} \frac{2}{v} \exp(-Sv/2a_1^2) \frac{2}{\sqrt{2\pi S/4}} \exp(-2a_1^2/S) = 2\exp\left(-2\sqrt{v}\right)/v. \tag{A36}$$

We are especially interested in the distribution of variances among sites that exceed some threshold contribution v^*. As we discuss in the main text and in Section 6, to a first approximation, the loci identified in a GWAS would be those with contributions to additive variance that exceed the study's threshold contribution v^*. In particular, our inferences based on GWAS data rely on fitting the probability density of the number of segregating sites with variance v that exceed a given threshold contribution v^* (Section 7). This probability density is:

$$\mathrm{f}(v|S) = \rho(v|S)/\mathrm{K}(v^*|S), \tag{A37}$$

where

$$\mathrm{K}(v^*|S) \equiv \int_{v>v^*} \rho(v|S) \tag{A38}$$

is the expected number of segregating sites with contributions to variance exceeding v^* per unit mutational input.

In our analysis, we focus on the expected proportion of additive genetic variance arising from sites that exceed a threshold contribution v^*, which approximates the heritable variance explained in GWAS. This proportion is given by

$$\mathrm{G}(v^*|S) = \frac{\int_{v>v^*} v\,\rho(v|S)}{\int_v v\,\rho(v|S)} = \frac{\int_{v>v^*} v\,\rho(v|S)}{E(V|S)}. \tag{A39}$$

Given a distribution of selection coefficients, $\mathrm{f}(S)$, the corresponding proportion is

$$\mathrm{G}(v^*) = \frac{\int_S \mathrm{G}(v^*|S)\mathrm{E}(V|S)\mathrm{f}(S)}{\int_S \mathrm{E}(V|S)\mathrm{f}(S)} = \int_S \mathrm{G}(v^*|S) \frac{\mathrm{E}(V|S)\mathrm{f}(S)}{\int_S \mathrm{E}(V|S)\mathrm{f}(S)}. \tag{A40}$$

The dependences of the proportion of variance $G(v^*|S)$ and the number of sites $K(v^*|S)$ on the strength of selection S for cases without pleiotropy and with extensive pleiotropy are shown in Figs. 3 and A3, respectively. We rely on Eqs. A32, A33, A35, A36, A38 and A39 to derive simplified forms for these summaries in the effectively neutral and strongly selected limits (Table A1). While the expressions for the effectively neutral limit were derived for $S \ll 1$, in practice they provide a decent approximation when $S < 1$ (Fig. A4a & b). In the strong selection limit ($S \gg 1$), the expressions for the case without pleiotropy provide a decent approximation for $S > 30$ (Fig. 3a), whereas with extensive pleiotropy they already work quite well for $S > 5$ (Fig. 3b).

Selection	Effectively neutral ($S \ll 1$)		Strongly selected ($S \gg 1$)	
# of traits	$n = 1$	$n \gg 1$	$n = 1$	$n \gg 1$
$E(V\|S)$	$S/2$		1	
$G(v^*\|S)$	$\sqrt{1-8v^*/S}$	$\exp(-4v^*/S)$	$\exp(-2v^*)$	$(1+2\sqrt{v^*})\exp(-2\sqrt{v^*})$
$K(v^*\|S)$	$4 \cdot \text{artanh}(\sqrt{1-8v^*/S})$	$2 \cdot I(4v^*/S)$	$2 \cdot I(2v^*)$	$4 \cdot I(2\sqrt{v^*})$

Table A1. Limits for the expected proportion of variance and expected number of sites corresponding to sites that exceed a threshold contribution to additive genetic variance v^*. $I(x) \equiv \int_{t>x} \exp(-t)/t$ is an exponential integral and artanh is the inverse hyperbolic tangent.

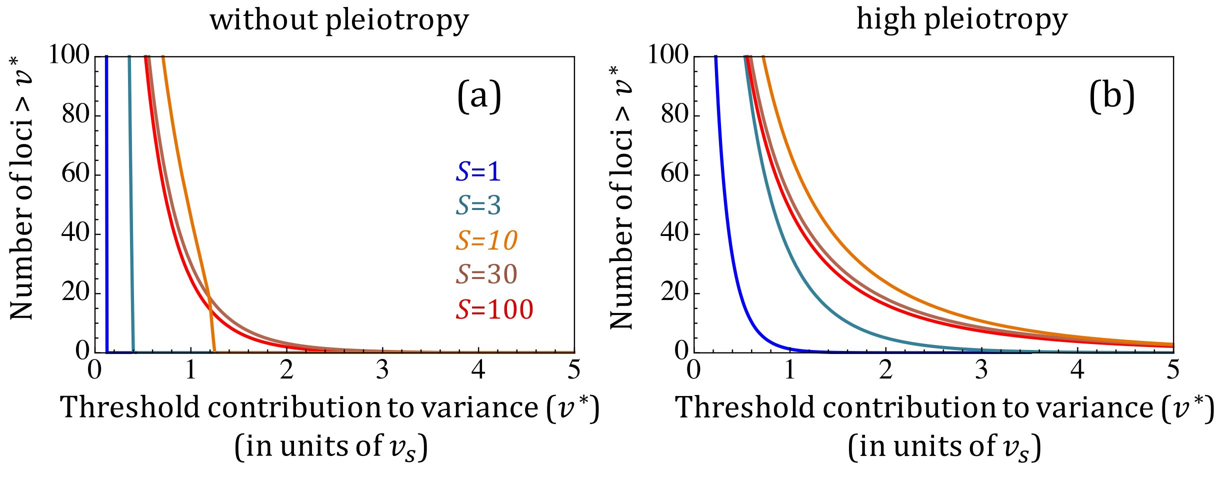

Figure A3. The number of loci per Mb contributing more than v^* to the variance, as a function of v^*, in the case without pleiotropy, $n = 1$ (a), and in the high pleiotropy limit, $n \gg 1$ (b). We assume a constant population size of 20,000, with a mutation rate of $1.2 \cdot 10^{-8}$ (5).

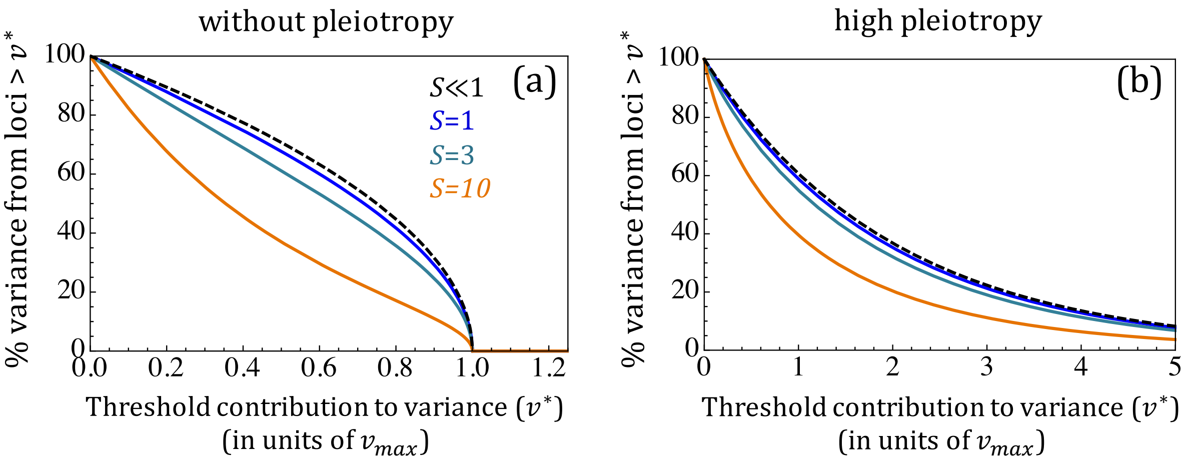

Figure A4. The proportion of additive genetic variance that arises from sites that contribute more than the value on the x-axis, for a single trait (a) and in the pleiotropic limit (b). We show the x-axis in units of $v_{max} = (S/8) \cdot v_s$ in order to evaluate the approximations in the effectively neutral limit (in dashed black; Eqs. 14 & 16, which appear in the Results section of the main text); note that v_{max} is not the maximal variance in cases with pleiotropy.

Both the proportion of variance, $\mathrm{G}(v^*|S)$, and number of variants, $\mathrm{K}(v^*|S)$, appear to always increase with the degree of pleiotropy, n (Fig. A5). We do not have a proof for this property but can suggest an intuitive explanation. Without pleiotropy (n=1), the selection coefficient determines the effect size, such that any contribution v^* to genetic variance corresponds to a specific minor allele frequency q^*. The sites with contributions $v > v^*$ are therefore those with minor allele frequencies $q > q^*$. Pleiotropy causes sites with a given selection coefficient to have a distribution of effect sizes on the trait under consideration. As a result, some sites with frequencies above q^* end up with contributions to variance below v^* while others exceed v^*. To understand how this affects $\mathrm{G}(v^*|S)$, recall that for any selection coefficient, the density of variants always rapidly increases as q^* decreases. As long as the contribution v^* and the corresponding frequency without pleiotropy q^* are not close to 0, we may therefore expect that introducing pleiotropy would result in pushing more sites above v^* than below v^*, resulting in a net increase to the proportion $\mathrm{G}(v^*|S)$. For the same reasons, the number of variants with $v > v^*$ shows a similar behavior and also grows with n.

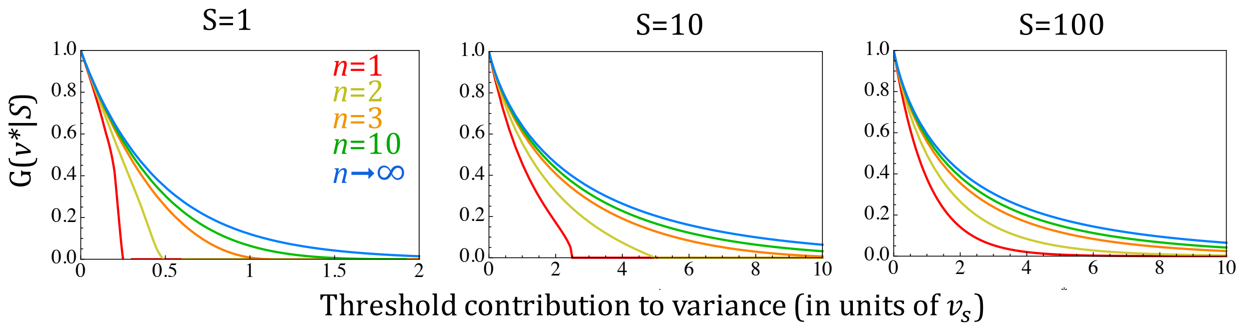

Figure A5. The effect of pleiotropy on the proportion of the variance explained by sites contributing more then v^* to the variance, $G(v^*|S)$ (see Eq. A39). For all selection coefficients, the proportion of variance explained increases as the number of traits, i.e., the degree of pleiotropy, increases.

3.3. Comparing predictions against simulations

We tested our theoretical derivations for the total genetic variance and its distribution among sites against forward computer simulations. The code and documentation are available at https://github.com/sellalab/GenArchitecture. The simulation implements the model as specified in the main text, with the following additional details and one exception. First, we assume the infinite sites model for mutation. Second, the distribution of scaled selection coefficients, or equivalently the distribution of mutation sizes (see Eq. 7, which appears in the Results section of the main text), is taken to be a Gamma distribution, with specified parameters (see below). For computational efficiency, we use fecundity rather than viability selection; however, we ran a smaller number of simulations to verify that this choice does not lead to a detectable difference in the results. Each simulation is run for a burn-in period of $10N$ generations, to ensure convergence to the steady state behavior, before the variances at segregating sites are measured.

We explore a range of parameter values chosen to balance biological plausibility (see Section 4) and manageable running times. Notably, we used a population size of $N = 1000$, with a burn-in time of 10,000 generations. We vary the number of traits to include $n = 1, 3, \& 10$, and vary the mutation rate per haploid genome per generation within the range $1/2\text{N} \leq U \leq 1$ (see Section 4), including U=0.0005, 0.001. 0.002, 0.005, 0.01, 0.02, 0.05, 0.1, 0.2, 0.5 & 1. Selection coefficients are chosen from an exponential distribution (setting the shape parameter for the Gamma distribution to 1) with means $E(S)$ = 0.1, 10, and 50. For

simplicity, we take $w^2 = 1$, which is equivalent to choosing the units used to measure effect sizes.

The simulation results for the total genetic variance and its distribution among sites are in close agreement with our theoretical predictions (Fig. A6). Specifically, within the parameter ranges that we assume, i.e., when $1/2N \ll \sigma^2/w^2 \ll 1$ (see Section 4), the total genetic variances measured in simulations are indistinguishable from our predictions (Fig. A6a). Moreover, simulations and prediction seem to agree even when $\sigma^2/w^2 \leq 1/2N$, although we consider this range to be less relevant, given our focus on highly polygenic traits (see Section 4.4). We also compare simulated and predicted distributions of variances among sites, in terms of $G(v)$, the proportion of the variance arising from sites that contribute more than v (Eq. A40), and find them to be in close agreement (Fig. A6b).

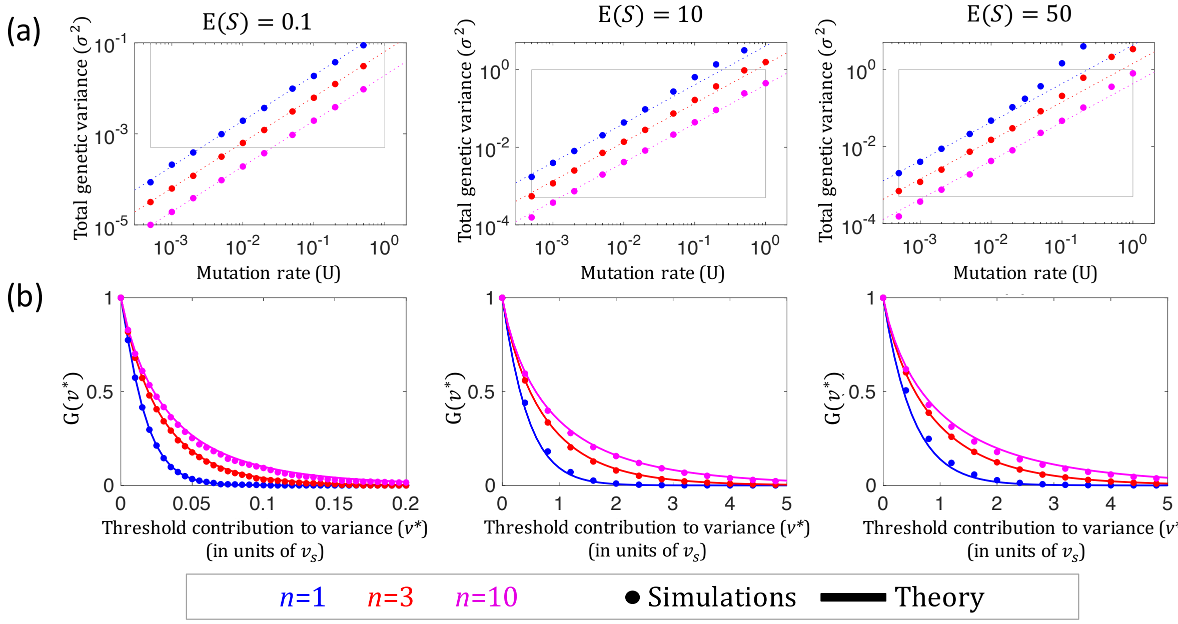

Figure A6. Testing theoretical predictions for genetic variance against simulation results. (a) Total genetic variance (in units of w^2) as a function of the mutation rate. The biologically relevant range of mutation rates, $1/2N \ll U \ll 1$, and the range in which we expect our predictions to be valid, $w^2/2N \ll \sigma^2 \ll w^2$, are marked by a grey box. (b) The distribution of variances among sites, for $U = 0.01$; $G(v^*)$ is the proportion of variance from sites contributing more than v^* (Eq. A40). Error bars represent one standard deviation. For each set of parameters, the number of simulations was chosen to obtain standard deviations below 10%. In practice, we often obtain much smaller standard deviations, which is why most error bars are too small to be visible.

We ran two additional variations on the basic simulation procedure (also available at https://github.com/sellalab/GenArchitecture): one to explore the effects of a shift in the

optimal phenotype (Section 5.1 and Fig. A7) and another to explore the effects of asymmetric mutational input (Section 5.2 and Fig. A8). To these ends, for simplicity, we consider the case without pleiotropy, i.e., with $n = 1$. In the first, after the $10N$ generations burn-in period, we introduce a shift in the optimal phenotype, and trace the allelic behavior over an additional 4,000 generations (see Section 5.1). In the second, after the $10N$ generations burn-in period, we introduce asymmetry in the rates of trait increasing and decreasing mutations, and trace the allelic trajectories over an additional 10,000 generations. The parameters of these simulations are detailed in Sections 5.1 and 5.2, respectively.

4. Justification for assumptions

Here, we justify the assumptions that we relied upon in deriving the first two moments of change in allele frequency (see Section 2.1; modeling assumptions are motivated in the introduction of the main text). We rely in part on self-consistency arguments, which should not be mistaken for being circular: specifically, we make assumptions about the behavior of the system and show that the solution to which we arrive satisfies these assumptions.

4.1. Normal and isotropic phenotypic distribution around the optimum

The assumption that the phenotypic distribution is well approximated by a normal distribution stems from the additive model of quantitative traits. By assuming that the phenotype arises from many additive contributions and that these additive contributions arise from some underlying distribution, normality follows from the law of large numbers. In terms of model parameters, we would expect normality to hold if the rate of mutations affecting the trait is sufficiently large, i.e., when $2NU \gg 1$.

We further assume that the phenotypic distribution is isotropic and its mean is at the optimum. Isotropy of the phenotypic distribution follows from assuming isotropy in the mutational input. In Section 5.4, we explore the consequences of anisotropy in the mutational input. In Section 4.4, we further show that the fluctuations of the mean phenotype around the optimum over time have negligible effects on allelic dynamics; a similar argument applies to fluctuations in the variance.

4.2. The phenotypic variance satisfies $\sigma^2 \ll w^2$

With the mean phenotype centered at the optimum, requiring that $\sigma^2 \ll w^2$ is equivalent to assuming that moving a standard deviation away from the mean phenotype entails only a minor reduction in fitness. This seems plausible for many phenotypes: if, for example, this assumption did not hold for human height, then individuals whose height is a standard deviation or more away from the population mean would suffer a substantial reduction in fitness. Arguably, deviations from the mean height would then be recognized as a very common and severe disease.

Another line of argument that it is likely that $\sigma^2 \ll w^2$ is based on our results. If we assume that mutations are strongly selected, then our results suggest that

$$\sigma^2 = 2NU \cdot v_s = 4Uw^2/n. \qquad \text{(A41)}$$

It follows that if the rate of mutations affecting the phenotype under consideration satisfies $U \ll 1$ then $\sigma^2 \ll w^2$. The number of mutations per diploid human genome per generation is estimated to be ~60 (5), and less than 10% of the genome is assumed to be functional (6), suggesting that the number of de novo mutations with any effect on function is less than 3 per haploid per generation. It then seems plausible that the (haploid) mutation rate affecting a specific trait satisfies $U \ll 1$. Assuming that mutations are weakly selected increases the variance in Eq. A41 only moderately and assuming the mutations are effectively neutral would suggest it is much smaller, leaving the above argument intact.

4.3. Mutational effect sizes satisfy $a^2 \ll w^2$

As we argued in the introduction of the main text, variants for which the stronger condition $a^2 \ll \sigma^2$ holds account for most or all of the heritability explained in GWAS for many traits (e.g., 7, 8-10). Moreover, evidence for many traits suggests that the same is true for the variants that underlie the heritability that remains to be explained (11-14). Indeed, for this assumption to be violated, much of the genetic variance would have to arise from mutations that have a very large impact on fitness (i.e., with s on the order of 1). While this may be the case for some diseases (e.g., autism (15)), it does not appear to be the case for most quantitative traits that have been examined.

4.4. Deviations of the mean phenotype from the optimum can be neglected

In reality, the mean phenotype of the population fluctuates around the optimum. Here, we derive equations for the dynamic of the mean phenotype in order to estimate the magnitude and timescale of these fluctuations. We then show that these fluctuations have a negligible effect on the first two moments of change in allele frequency and thus on the results that follow from these moments.

We begin by deriving the first and second moment of change in mean phenotype. To this end, we assume the distribution of phenotypes is a multivariate normal centered around a mean phenotype, $\bar{r}$, i.e. that

$$f(\vec{r}) = \frac{1}{(2\pi\sigma^2)^{n/2}} \exp\left(-\frac{|\vec{r}-\bar{r}|^2}{2\sigma^2}\right). \quad \text{(A42)}$$

The expected change in mean phenotype due to selection in one generation is therefore

$$E(\Delta \bar{r}) = \frac{\int_{\vec{r}} f(\vec{r})W(\vec{r})\vec{r}}{\int_{\vec{r}} f(\vec{r})W(\vec{r})} - \bar{r} = -\frac{\sigma^2}{w^2+\sigma^2}\bar{r} \approx -\frac{\sigma^2}{w^2}\bar{r}. \quad \text{(A43)}$$

By the same token, the variance in $\Delta\bar{r}$ is simply the sampling variance

$$\mathrm{V}(\Delta \bar{r}) \approx \frac{\sigma^2}{N}, \tag{A44}$$

where in both cases we relied on the assumption that $\sigma^2 \ll w^2$.

These two moments define an Ornstein-Uhlenbeck process in $\bar{r}$, allowing us to rely on well-known results (16). Notably, when the mean phenotype $\bar{r}$ starts far from the optimum, it decays exponentially to the optimum with exponent σ^2/w^2 (see Section 5.1 below). At steady state, $\bar{r}$ will fluctuate with mean zero and $\mathrm{E}(\bar{r}^2) = nw^2/2N$ over a time scale of w^2/σ^2 generations. The typical displacement of $\bar{r}$ in any given direction will be $\sqrt{w^2/2N}$, reflecting a balance between drift and the pull of selection toward the optimum.

Next we show that these fluctuations of the mean have negligible effects on allelic trajectories. To this end, we derive the first two moments of change in allele frequency, but this time, we include the effect of the displacement of $\bar{r}$ from the optimum. While the second moment remains the same, the first moment becomes

$$\mathrm{E}(\Delta q) \approx -\frac{\bar{r}\cdot\vec{a}}{w^2}pq - \frac{a^2}{w^2}pq\left(\frac{1}{2} - q\right) = -\frac{\bar{r}_a}{\sqrt{w^2/2N}}\frac{\sqrt{S}}{2N}pq - \frac{S}{2N}pq\left(\frac{1}{2} - q\right), \tag{A45}$$

where $\bar{r}_a$ is $\bar{r}$'s component in the direction of $\vec{a}$. However, our analysis establishes that $\frac{\bar{r}_a}{\sqrt{w^2/2N}}$ is a scalar on the order of 1, which fluctuates around zero on a timescale of w^2/σ^2. We can therefore compare the first term in the above equation, which represents directional selection, and the second term, which represents stabilizing selection. When stabilizing selection is strong, $S \gg 1$, the stabilizing selection term dominates over the directional selection term. In contrast, when selection is weak, i.e., $S \approx 1$ or smaller, then in any given generation, the directional term is not necessarily negligible. However, in this case, both terms affect substantial change in allele frequency only over a timescale of $2N$ generations; on this timescale, if $2N \gg w^2/\sigma^2$, the directional effect would average to zero. The directional term will become important only when $2N \leq w^2/\sigma^2$, that is $\sigma^2 \leq w^2/2N$. For σ^2 to be that small, virtually all alleles must have $S \ll 1$, such that their trajectories will be determined by drift, not selection. In summary, regardless of the selection acting on an allele, fluctuations of the mean phenotype around the optimum will have a negligible effect on its trajectory.

5. Model robustness

In this section, we consider the sensitivity of our results to relaxing some of the simplifying modeling assumptions about selection and mutation. Specifically, we show our results to be robust to moderate changes to the optimal phenotype; small asymmetry in the mutational input; the presence of major loci maintained at high frequency by selection on traits that are not included in the model; as well as to most forms of anisotropic mutation.

5.1. Changes to the optimal phenotype

We first consider how changes to the optimal phenotype over time would affect our results. It is easy to imagine how events such as migration from Africa to Europe or the onset of agriculture may have introduced rapid changes in optimal phenotypes. In order to evaluate the potential impact of such events, we consider how an instantaneous change to the optimal phenotype would affect the allelic dynamics. Similar models have recently been analyzed in the limit of infinite population size (17, 18).

We begin by considering how such an instantaneous change to the optimum would affect the mean phenotype. If the shift to the optimum is small, on the order of the fluctuations in the mean phenotype at steady state or smaller, then the arguments provided in Section 4.4 will still hold and the shift would have a negligibly small effect on our results. We therefore assume that the shift in optimum, $\vec{z}$, is large compared to the scale of fluctuations ($z^2 \gg w^2/2N$). This assumption means that we can use a deterministic approximation (based on Eq. A43) and describe the change in mean phenotype in a single generation by

$$\Delta \bar{r} \approx \mathrm{E}(\Delta \bar{r}) = -\frac{\sigma^2}{w^2}(\bar{r} - \vec{z}) \qquad \text{(A46)}$$

(neglecting higher moments). Further assuming that the mean phenotype was at the optimum, $\vec{0}$, before the optimum shifted (at time $t = 0$) and neglecting changes to the genetic variance σ, we find that

$$\bar{r}(t) = \vec{z}\left(1 - \exp\left(-\frac{\sigma^2}{w^2}t\right)\right). \qquad \text{(A47)}$$

Thus, the mean $\bar{r}$ adapts to the new optimum on a timescale of w^2/σ^2 generations (see (19) for a similar derivation).

We can rely on this approximation to learn when a shift in optimum will have negligible effects on allele trajectories. Recalling Eq. A45, the first moment of change in allele frequency is given by

$$E(\Delta q) \approx -\frac{(\bar{r}(t)-\vec{z})\cdot\vec{a}}{w^2}pq - \frac{a^2}{w^2}pq\left(\frac{1}{2}-q\right), \quad \text{(A48)}$$

where, based on our approximation (Eq. A47), the directional selection term introduced by the shift in optimum takes the time-dependent form

$$E(\Delta_D q) = -\frac{(\bar{r}(t)-\vec{z})\cdot\vec{a}}{w^2}pq \approx \frac{\vec{z}\cdot\vec{a}}{w^2}\exp\left(-\frac{\sigma^2}{w^2}t\right)pq. \quad \text{(A49)}$$

The effect of this directional term over the entire adaptive trajectory can be quantified by comparing the expected allele frequency after adaptation to the shift, q_D, with initial frequency before the shift, q_0, i.e.,

$$\ln(q_D/q_0) = \int_t \frac{E(\Delta_D q)}{q(t)} = \frac{\vec{z}\cdot\vec{a}}{w^2}\int_t p(t)\exp\left(-\frac{\sigma^2}{w^2}t\right) < \frac{\vec{z}\cdot\vec{a}}{w^2}\int_t \exp\left(-\frac{\sigma^2}{w^2}t\right) = \frac{\vec{z}\cdot\vec{a}}{\sigma^2}. \quad \text{(A50)}$$

This result suggests that the relative change in allele frequency will be negligible so long as

$$(\vec{z}\cdot\vec{a})/\sigma^2 \ll 1. \quad \text{(A51)}$$

This condition suggests that mutations with smaller effects would be less affected by the shift in the optimum. It further suggests that alleles that satisfy $a^2 \ll \sigma^2$, as appears to be the case for most loci discovered in GWAS (e.g., 7, 8-10), will be negligibly affected by shifts in optimum on the order of the total genetic variation (i.e., $z \leq \sigma$). These analytic predictions are confirmed by simulations (Fig. A7).

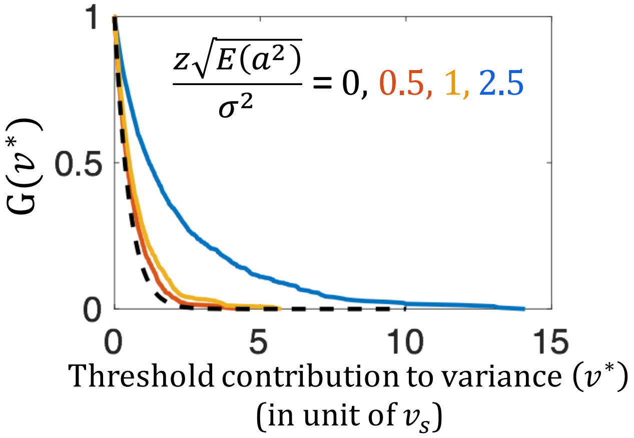

Figure A7. Distribution of the contributions of sites to variance after a shift in the optimum. The y-axis is the proportion of the variance explained by sites that contribute more than v^* to the variance. The theoretical prediction without adaptation is shown in dashed black, and simulation results for different shifts in the optimal phenotype are shown in color. When the root mean square of $(\vec{z}\cdot\vec{a})/\sigma^2$ becomes larger than 1, directional selection substantially affects allele frequencies and therefore the contributions of sites to variance, as predicted by Eq. A51. (Since mutation is symmetric the mean of $(\vec{z}\cdot\vec{a})/\sigma^2$ is zero and we quantify its characteristic value by its root mean square $z\sqrt{E(a^2)}/\sigma^2$.)

Simulations were run with an exponential distribution of selection coefficients with $\mathrm{E}(S) = 25$, $N = 1{,}000$, $n = 1$, $U = 0.01$, and a burn-in time of 10,000 generations. Results were taken 50 generations after the shift in optimum, which, for these parameters, is just after the population mean has reached the new optimum.

5.2. Asymmetric mutational input

In this section, we consider the sensitivity of our results to asymmetries in the mutational input, i.e., to the case in which mutations in a given direction in trait space are more likely to arise than mutations in the opposite direction (see (20) for treatment of this problem in the limit of high per-site mutation rate).

An asymmetric mutational input introduces a shift in the mean phenotype, $\bar{r}$, every generation. With new mutations arising at frequency $1/2N$, the expected shift is

$$\Delta_{\mathrm{M}}\bar{r} = 4NU\, \mathrm{E}_{\mathrm{M}}(\vec{a}) \cdot 1/2N = 2U\mathrm{E}_{\mathrm{M}}(\vec{a}), \quad \text{(A52)}$$

where E_{M} is the expectation over newly arising mutations. For each trait, effects have a characteristic size $\sqrt{\mathrm{E}(a^2)/n}$=$\sqrt{v_S}\sqrt{\mathrm{E}(S/4)}$. The characteristic effect size sets the scale for the maximal shift in any direction, that is $\|\Delta_M\bar{r}\|$ is of the order of $2U\sqrt{\mathrm{E}(a^2)/n}$ or smaller. We therefore parameterize the shift in mean phenotype due to new mutations by

$$\Delta_{\mathrm{M}}\bar{r} = 2U\mathrm{E}_{\mathrm{M}}(\vec{a}) = 2U\sqrt{\mathrm{E}(a^2)/n}\,\vec{b}, \quad \text{(A53)}$$

where the vector $\vec{b}$ parameterizes the strength and direction of the bias and $b = \|\vec{b}\|$ is assumed to be << 1.

At steady state, the mutational shift must be offset by selection, such that

$$\Delta_{\mathrm{M}}\bar{r} + \Delta_{\mathrm{D}}\bar{r} + \Delta_{\mathrm{S}}\bar{r} = \vec{0}, \quad \text{(A54)}$$

where $\Delta_{\mathrm{D}}\bar{r}$ and $\Delta_{\mathrm{S}}\bar{r}$ are the expected shifts due to directional and stabilizing selection, respectively. We previously found that the expected directional shift is

$$\Delta_D\bar{r} = -\frac{\sigma^2}{w^2}\bar{r}, \quad \text{(A55)}$$

where $\bar{r}$ denotes the mean phenotype (see Eq. A43). As we show next, when mutations are strongly selected, stabilizing selection offsets the mutational shift to maintain the mean phenotype at the optimum, implying that directional selection is negligible. In contrast, when mutations are effectively neutral, stabilizing selection is negligible and a directional term might not be negligible by comparison. However, as long as asymmetry is small, $b \ll 1$, we show that this directional term is not large enough to change the allele dynamics,

both when all mutations are effectively neutral and when some mutations are strongly selected.

First, we consider the shift in mean phenotype due to stabilizing selection. This shift arises because, with asymmetric mutational input, the distribution of phenotypes becomes skewed. Therefore, stabilizing selection may change the mean phenotype even if it is at the optimum. We have already shown (Eq. A15) that the expected change in allele frequencies per generation due to stabilizing selection at any given site i is

$$E(\Delta q_i) = -\frac{a_i^2}{w^2} p_i q_i \left(\frac{1}{2} - q_i\right). \tag{A56}$$

The expected change in mean phenotype can then be calculated by adding up the contributions over sites

$$\Delta_S \bar{r} = -\mathrm{E}\left(\sum_i 2\vec{a}_i \frac{a_i^2}{w^2} p_i q_i \left(\frac{1}{2} - q_i\right)\right). \tag{A57}$$

The right-hand side of this equation reflects the skewness of the phenotypic distribution. Indeed, in one dimension, it can be shown that

$$\Delta_S \bar{r} = -\frac{\mu_3(r)}{2w^2}, \tag{A58}$$

with $\mu_3(r)$ being the third central moment of the phenotypic distribution. In n-dimensions, for every direction x,

$$\Delta_S \bar{r}_x = -\frac{1}{2w^2}\mathrm{E}((\vec{r} - \bar{r})_x(\vec{r} - \bar{r})^2) = -\frac{1}{2w^2}\sum_k \mu_3(\vec{r})_{xkk}, \tag{A59}$$

with $\mu_3(\vec{r})_{jkl} = \mathrm{E}\big((\vec{r} - \bar{r})_j(\vec{r} - \bar{r})_k(\vec{r} - \bar{r})_l\big)$.

When sites are under strong selection, $\Delta_S \bar{r}$ takes a simple form. Assuming the asymmetry is small, the shift due to stabilizing selection can be expanded in orders of b. The leading term in the frequency distribution takes the same form as it does without the bias. For strongly selected alleles with no bias, $q \ll 1$ and therefore the frequency dependence in this term can be approximated by $pq\left(\frac{1}{2} - q\right) \approx \frac{1}{2}q$. Moreover, q scales with $1/a^2$, implying that the distribution of $a^2 q$ is independent of $\vec{a}$ and that $\mathrm{E}(a^2 q) = w^2/N$ (see Section 3.1). Therefore, when all sites are strongly selected, the leading term in the shift due to stabilizing selection is

$$\Delta_S^0 \bar{r} = -\mathrm{E}\left(\sum_i 2\vec{a}_i \frac{a_i^2}{w^2}\frac{q_i}{2}\right) = -\frac{\mathrm{E}(a^2 q)}{w^2}\mathrm{E}\left(\sum_i \vec{a}_i\right) = -\frac{1}{N}2NU\mathrm{E}_M(\vec{a}) = -\Delta_M \bar{r}. \tag{A60}$$

Thus, to a first order in b, the shift of the mean phenotype due to stabilizing selection offsets the mutational shift, implying that there will be no directional term and that the allele dynamics will not be affected by asymmetry.

When alleles are instead effectively neutral, then $a^2/w^2 \ll 1/2N$ (see Section 2.2) and allele frequencies are well approximated by the neutral sojourn time, $\tau(q) \approx 2/q$. The shift due to stabilizing selection then satisfies

$$\Delta_S\bar{r} = -\mathrm{E}\left(\sum_i 2\vec{a}_i \frac{a_i^2}{w^2} p_i q_i \left(\frac{1}{2} - q_i\right)\right) \approx -\mathrm{E}\left(pq\left(\frac{1}{2} - q\right)\right) \mathrm{E}\left(\sum_i 2\vec{a}_i \frac{a_i^2}{w^2}\right)$$

$$= -\frac{1}{6}\,\mathrm{E}\left(\sum_i 2\vec{a}_i \frac{a_i^2}{w^2}\right) \ll -\frac{1}{N}\mathrm{E}(\sum_i \vec{a}_i\,) = -\Delta_M\bar{r}, \qquad \text{(A61)}$$

implying that it makes a negligible contribution to offsetting the mutational shift. In this case, the mutational effect on the mean phenotype is therefore offset by directional selection, where

$$\Delta_D\bar{r} = -\frac{\sigma^2}{w^2}\bar{r} \approx -\Delta_M\bar{r}, \qquad \text{(A62)}$$

indicating a displacement of the mean phenotype from the optimum

$$\bar{r} = \frac{w^2}{\sigma^2}\Delta_M\bar{r}. \qquad \text{(A63)}$$

This displacement introduces a directional selection term into the first moment of change in allele frequency that, if large enough, could alter allele dynamics (see Section 4.4). However, when all alleles are effectively neutral, we have

$$\bar{r} = \frac{w^2}{\sigma^2}\Delta_M\bar{r} = \frac{w^2}{2NUv_s\mathrm{E}(S)/2}2U\sqrt{v_s}\sqrt{\mathrm{E}(S/4)}\,\vec{b} = \frac{1}{2N}\frac{2w^2}{\sqrt{v_s}\sqrt{\mathrm{E}(S)}}\,\vec{b}, \qquad \text{(A64)}$$

and therefore the scaled directional selection coefficient, for an allele with effect size $\vec{a}$ and scaled stabilizing selection coefficient $S = 2N\frac{a^2}{w^2}$, is of the order of

$$2N\frac{\bar{r}\cdot\vec{a}}{w^2} = \frac{2b_a a}{\sqrt{v_s}\sqrt{\mathrm{E}(S)}} \sim \frac{2(b/\sqrt{n})a}{\sqrt{v_s}\sqrt{\mathrm{E}(S)}} = \frac{b\sqrt{v_s}\sqrt{S}}{\sqrt{v_s}\sqrt{\mathrm{E}(S)}} = \sqrt{\frac{S}{\mathrm{E}(S)}}\;b, \qquad \text{(A65)}$$

with $b_a \sim b/\sqrt{n}$ being the projection of $\vec{b}$ in the direction of $\vec{a}$. Since $b \ll 1$, for all alleles other than those with unusually large selection coefficients, the scaled directional selection coefficient will be much smaller than 1 and the trajectories will still be determined by drift and not selection. Even in this case, therefore, we do not expect asymmetry to affect allele dynamics.

Next, we consider the case where there is a mix of effectively neutral and strongly selected mutations. The existence of strongly selected mutations in addition to effectively neutral

ones reduces the deviation of the mean phenotype from the optimum. Denoting the proportion of strongly selected mutations by p_s, we have

$$\bar{r} = \frac{w^2}{\sigma^2} U(1 - p_s)\sqrt{v_s}\sqrt{\mathrm{E}(S^{e.n.})}\,\vec{b}, \tag{A66}$$

where $\mathrm{E}(S^{\mathrm{e.n.}}) \leq 1$ is the mean scaled stabilizing selection coefficient for effectively neutral mutations. Since $\sigma^2 > 2NUp_s v_s$, we can then obtain an upper bound to the magnitude of scaled directional selection coefficient for an allele with effect size $\vec{a}$ and scaled stabilizing selection coefficient $S = 2N\frac{a^2}{w^2}$:

$$2N\frac{\bar{r}\cdot\vec{a}}{w^2} = 2N\frac{1}{\sigma^2}U(1 - p_s)\sqrt{v_s}\sqrt{\mathrm{E}(S^{e.n.})}\,\vec{b}\cdot\vec{a}$$

$$< \frac{1}{Up_s v_s}U(1 - p_s)\sqrt{v_s}\sqrt{E(S^{e.n.})}\,\vec{b}\cdot\vec{a} \sim \frac{1-p_s}{2p_s}\sqrt{E(S^{e.n.})}\,\sqrt{S}\,b. \tag{A67}$$

With a substantial proportion of strongly selected sites, $(1 - p_s)/2p_s$ is of the order of 1, and therefore $\frac{1-p_s}{2p_s}\sqrt{\mathrm{E}(S^{e.n.})}\,b \ll 1$. This condition implies that for effectively neutral alleles (i.e., $S \leq 1$), the scaled directional selection coefficient is $\ll 1$ and allele trajectories will be determined by genetic drift, whereas for strongly selected alleles (i.e., when $S \gg 1$), the scaled directional selection coefficient is $\ll S$ and therefore negligible compared to the scaled stabilizing selection coefficient.

Weakly selected alleles (with $1 < S < 30$) behave largely like strongly selected alleles except that stabilizing selection on them only partially cancels out the mutational bias (for example, for $S = 10$ only 85% of the bias is canceled). The rest of the bias is canceled by directional selection and therefore induces a small shift in the mean phenotype. It is straightforward to repeat the arguments given above and show that the shift in the mean phenotype for a trait with only weakly selected alleles or a mixture that includes weakly selected alleles negligibly affects allele trajectories.

Thus, we conclude that small asymmetry in mutation will not affect the allelic dynamic (see Fig. A8).

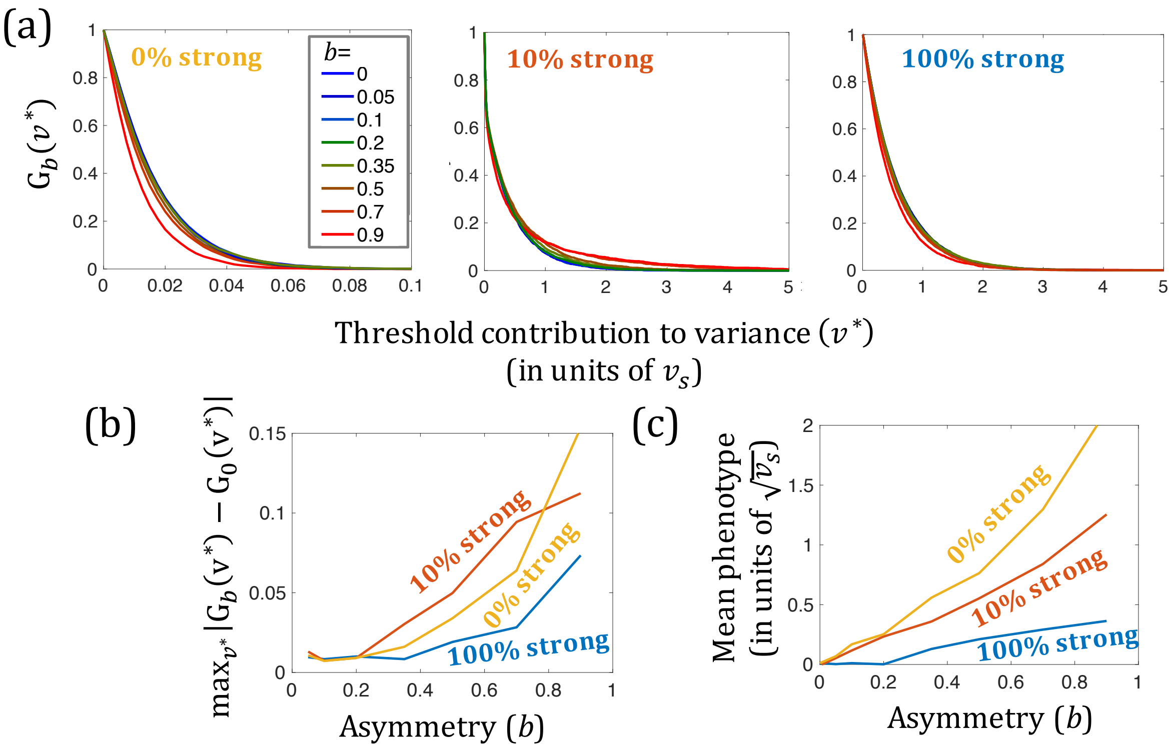

Figure A8. The effect of asymmetric mutational input on the contribution of sites to variance and the mean phenotype. (a) Proportion of genetic variance as a function of the threshold contribution to variance v^*, i.e., $G_b(v^*)$, for different bias strengths. (b) The maximal distance of $G_b(v^*)$ from $G_0(v^*)$, i.e. $\max_{v^*}|G_b(v^*) - G_0(v^*)|$, as a function of b. (c) The mean phenotype $\bar{r}$, in units of $\sqrt{v_s}$, as a function of mutational bias b. Simulations were run with $N = 1{,}000$, $n = 1$ and with different mixtures of effectively neutral (exponentially distributed with $E(S) = 0.1$) and strong (exponentially distributed with $E(S) = 50$) selection coefficients. Asymmetry was simulated by having more trait increasing than trait decreasing mutations; if β is the proportion of trait increasing mutations then the asymmetry coefficient is $b = 2\beta - 1$. As expected, for small biases (when $b \ll 1$), there are no substantial changes in the distribution of the contribution of sites to variance. Simulations were run with a 10,000 generations burn-in period without asymmetry and then 10,000 generations with asymmetry and averaged over many runs (>300), with the number of runs varied across plots keep errors in (a) below 1%.

5.3. Major effect loci

In this section, we show that our results are insensitive to the presence of major loci, i.e., individual loci that contribute substantially to quantitative genetic variation. We have in mind, for example, loci whose alleles are maintained at high frequencies by balancing selection on a Mendelian trait but have pleiotropic effects on the quantitative traits under

consideration (e.g., HLA loci (21, 22)). While such loci violate our assumptions, we show that they do not affect the dynamics at other loci that fulfill them.

To this end, we calculate the first two moments of change in allele frequency in the presence of a major locus. We denote the frequency and effect size of the focal allele by q and $\vec{a}$, and the frequency and effect size of the major allele by q_M and $\vec{a}_M$, respectively. As in our previous derivations (Section 2.1), the distribution of background phenotypic contribution from all other loci, $\vec{R}$, is well approximated by the normal distribution

$$\mathrm{f}\left(\vec{R}\middle|\vec{a}_M, q_M, \vec{a}, q\right) = \frac{1}{\left(2\pi(\sigma^2-\sigma_M^2)\right)^{n/2}} \exp\left(-\frac{\left(\vec{R}+2q\vec{a}+2q_M\vec{a}_M\right)^2}{2(\sigma^2-\sigma_M^2)}\right), \qquad \text{(A68)}$$

where σ_M^2 is the contribution to genetic variance from the major locus. The population mean remains close to the optimum because any shift caused by the major locus is quickly compensated for by the other loci (see Section 4.4). We then average over both this distribution and the three genotypes at the major locus to calculate the mean fitness associated with each genotype at the focal locus. Namely,

$$W_{00} = (1-q_M)^2 \int_{\vec{R}} \mathrm{f}\left(\vec{R}\middle|\vec{a}_M, q_M, \vec{a}, q\right) \mathrm{W}\left(\vec{R}\right) + 2q_M(1-q_M) \int_{\vec{R}} \mathrm{f}\left(\vec{R}\middle|\vec{a}_M, q_M, \vec{a}, q\right) \mathrm{W}\left(\vec{R}+\vec{a}_M\right)$$
$$+q_M^2 \int_{\vec{R}} \mathrm{f}\left(\vec{R}\middle|\vec{a}_M, q_M, \vec{a}, q\right) \mathrm{W}\left(\vec{R}+2\vec{a}_M\right), \qquad \text{(A69)}$$

and similarly for the other genotypes. In this way, we obtain the first moment of the change in allele frequency

$$\mathrm{E}(\Delta q) = -pq\frac{p(W_{00}-W_{01})+q(W_{01}-W_{11})}{\bar{W}} \approx -\frac{a^2}{w^2}pq\left(q-\frac{1}{2}\right), \qquad \text{(A70)}$$

which is the same as we derived in the absence of a major locus (Eq. A15). Similarly, we find the second moment to be unaffected.

5.4. Anisotropic mutation

In this section, we consider how relaxing the assumption that the distribution of newly arising mutations is isotropic in trait space would affect our results. As noted, we can always choose an orthonormal coordinate system centered at the optimum, in which the trait under consideration varies along the first coordinate and a unit change in other traits (i.e., in other coordinates) near the optimum have the same effect on fitness. There is, however, no obvious reason for the distribution of newly arising mutations to be isotropic in this coordinate system (see (23) for generalizations of Fisher's Geometric Model along similar lines).

Anisotropy in mutation does not affect the moments of change in allele frequency, as these depend only on the selection on an allele or equivalently on its effect size but not on its direction in trait space. Anisotropy could affect the distribution of allelic effect sizes on the focal trait conditional on the selection acting on them. Here, we provide heuristic arguments suggesting that, barring extreme cases, we can define an effective number of traits n_e and an effective strength of selection w_e^2 for which the relationship between selection and effect size in anisotropic models is well approximated by the relationship found for isotropic ones (Eqs. 9 & 11, which appear in the Results section of the main text; Section 1.2).

We focus on a family of anisotropic mutational distributions that can be described as a projection of a multivariate normal distribution on the unit sphere in trait space. Namely, we draw the size of a mutation $a = ||\vec{a}||$ from some distribution and to obtain its direction, we draw a vector $\vec{\alpha}$ from a multi-variate normal distribution $\text{MVN}(0, \boldsymbol{\Sigma})$ and normalize it, i.e.,

$$\vec{a} = a\frac{\vec{\alpha}}{\alpha}, \qquad \text{(A71)}$$

and therefore

$$a_1 = a\frac{\alpha_1}{\alpha}. \qquad \text{(A72)}$$

This family of mutational distributions gives us a mathematically tractable framework with which to examine the behavior of our model with anisotropy.

With anisotropy, the behavior of our model greatly depends on the relative contribution of the focal trait to selection, which we parameterize by

$$\gamma_1 \equiv \frac{\text{E}(\alpha_1^2)}{\text{E}(\alpha^2)} = \frac{\boldsymbol{\Sigma}_{11}}{\text{tr}(\boldsymbol{\Sigma})}. \qquad \text{(A73)}$$

When selection acts mainly on our focal trait, i.e. when $\gamma_1 \approx 1$, then $|\alpha_1| \approx \alpha$ and therefore $a_1 \approx \pm a$. Such a relationship between the strength of selection and effect size is well approximated by an isotropic model with $n_e = 1$. We therefore focus on cases in which there is a significant pleiotropic contribution to selection, i.e., γ_1 is substantially less than 1. Anisotropy then has two effects: the first is to introduce heterogeneity in the strength of selection on different traits and the second is to introduce correlations in the effects of a mutation on different traits, notably between the focal trait and others.

We first consider the case in which the strength of selection differs among traits, but traits are uncorrelated, corresponding to a diagonal covariance matrix, $\boldsymbol{\Sigma}$. When many traits have

a non-negligible contribution to selection, $\alpha^2 = \alpha_1^2 + \alpha_2^2 + \cdots \alpha_n^2$ would have a small coefficient of variation, i.e., $\mathrm{C_V^2}(\alpha^2) = \mathrm{V}(\alpha^2)/\mathrm{E}(\alpha^2)^2 \ll 1$, because of the law of large numbers. In this case,

$$a_1 = a\frac{\alpha_1}{|\vec{\alpha}|} = a\frac{\alpha_1}{\sqrt{E(\alpha^2)}}\left(1 + \mathrm{O}\left(\mathrm{C_V^2}(\alpha^2)\right)\right) \approx a\frac{\alpha_1}{\sqrt{\mathrm{E}(\alpha^2)}} = \frac{a}{\sqrt{1/\gamma_1}}\ \frac{\alpha_1}{\sqrt{\mathrm{E}(\alpha_1^2)}}, \quad \text{(A74)}$$

Since $\alpha_1/\sqrt{\mathrm{E}(\alpha_1^2)} \sim \mathrm{N}(0,1)$ and $s = \frac{1}{w^2}a^2$, this implies that, conditional on the selection coefficient S, the effect size on the focal trait will be distributed as

$$a_1 \sim \mathrm{N}\left(0, \frac{w^2}{n_e}s\right) \quad \text{(A75)}$$

with $n_e = 1/\gamma_1$. This is the same relationship between selection and effect size as in the high pleiotropy isotropic model with $n = n_e$ (Eq. 11, which appears in the Results section of the main text). This result suggests the concept of an effective number of traits, which can be thought of as the number of traits that have the same effect on fitness as the focal one and are required to produce the same strength of selection on alleles. The effective number of traits describes the distribution of effect sizes both in the limit of high pleiotropy $n_e \gg 1$ and low pleiotropy $n_e \approx 1$ and simulations show that it describes the distribution, at least qualitatively, also for intermediate values of n_e (Fig. A9).

However, there is an extreme scenario in which an effective number of traits cannot describe the distribution of effect sizes. This happens when $\mathrm{C_V^2}(\alpha^2) \geq 1$, that is when selection acts mainly on a small number of traits but our focal trait contributes very little to selection ($\gamma_1 \ll 1$). In this case, we might be tempted to use $n_e = 1/\gamma_1 \gg 1$ but, as Eq. A74 suggests, the high pleiotropy limit would be inadequate. In fact, the variance in selection on newly-arising mutations (due to the contribution of the selected traits) will result in a long-tailed distribution of effect sizes on the focal trait, which is not well-approximated by any isotropic model. Excluding these extreme cases, isotropic models provide a good approximation for the relationship between selection and effect size, even when there is heterogeneity in the strength of selection on different traits.

To illustrate the effect of heterogeneity in the strength of selection among traits, we consider a simple example in which all non-focal traits make the same contribution to selection and therefore can be modeled by

$$\boldsymbol{\Sigma} = \begin{pmatrix} 1 & 0 & 0 & \\ 0 & c^2 & 0 & \cdots \\ 0 & 0 & c^2 & \\ & \vdots & & \ddots \end{pmatrix}, \tag{A76}$$

where c^2 is the ratio between the expected fitness effects of non-focal and focal traits. In Fig. A9 we compare numerical results of this model with isotropic models with $n_e = 1/\gamma_1 = 1 + (n-1)c^2$.

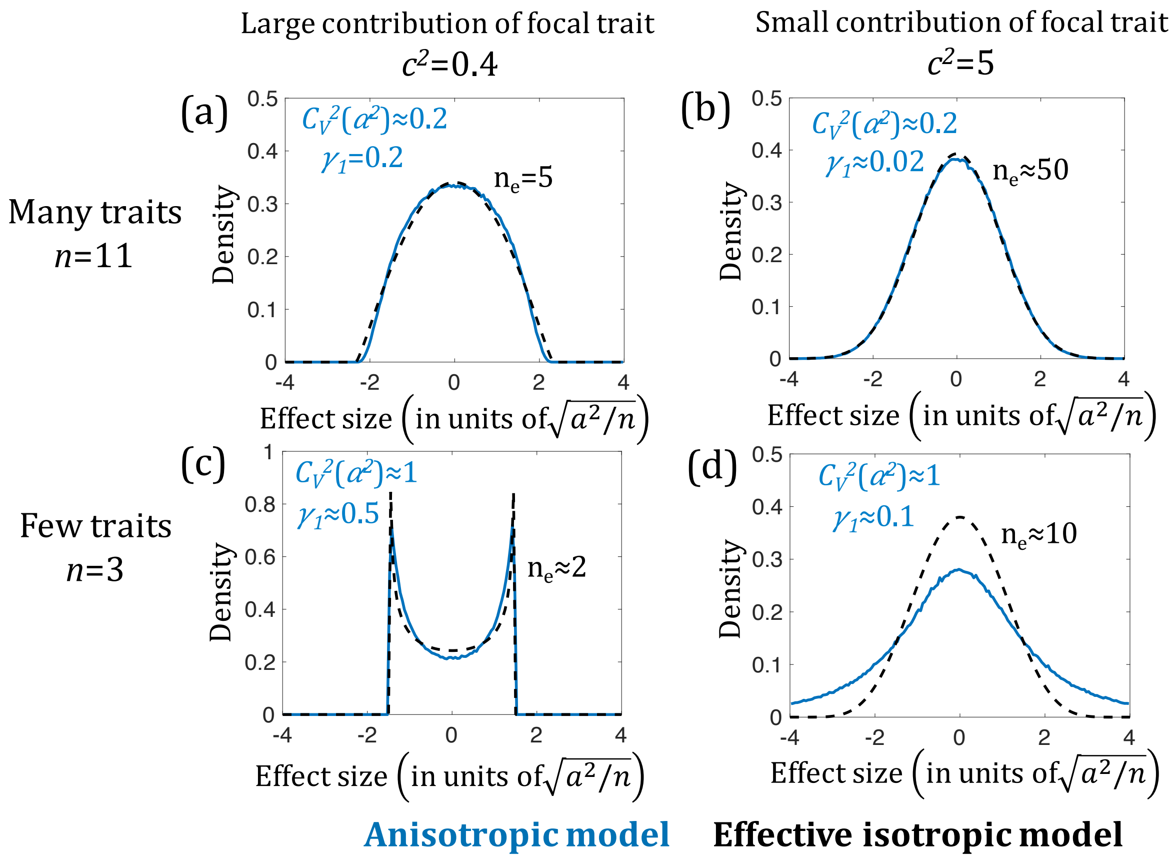

Figure A9. The effects of heterogeneity in the strength of selection on different traits on the distribution of effect sizes in the focal trait. Numerical results for models with the correlation matrix defined in Eq. A76 are shown in blue and the corresponding isotropic model in black dashes. When there are many selected traits, an isotropic model with $n_e = 1/\gamma_1 = 1 + (n-1)c^2$ provides a good approximation of the distribution of effect sizes, both when the focal trait contributes substantially to selection (a) and when it does not (b). When there are few traits, an isotropic model with $n_e = 1/\gamma_1 = 1 + (n-1)c^2$ provides a good approximation only when the focal trait contributes substantially to selection (c & d).

Next, we consider the case in which the effect sizes on different traits are correlated, i.e., when the covariance matrix $\boldsymbol{\Sigma}$ has off-diagonal terms. $a_1^2 \propto \alpha_1^2/\alpha^2$ and therefore we parameterize the effect of these terms using the correlation between α_1^2 and α^2, $\rho^2 \equiv$

$\mathrm{corr}(\alpha^2, \alpha_1^2)$. If the correlation is small, $\rho^2 \ll 1$, then our previous reasoning holds. In the other extreme, when all selected traits are highly correlated with the focal trait, i.e. $\rho^2 \approx 1$, then the proportional contribution of the focal trait to selection is constant, $\alpha_1^2/\alpha^2 \approx \mathrm{E}(\alpha_1^2)/\mathrm{E}(\alpha^2) = \gamma_1$, and the effect size is $a_1 = \pm\sqrt{\gamma_1}\, a$. This model is therefore equivalent to an isotropic one with $n_e = 1$ and $w_e^2 = \gamma_1 w^2$; the latter change corresponds to increasing the strength of selection on the focal trait to account for selection on the other, highly-correlated traits. Intermediate cases are more complex: while effect sizes are still of the order of $\sqrt{\gamma_1}\, a$, the shape of the distribution of effect sizes is intermediate between the single trait and high pleiotropy limits. Isotropic models with an effective number of traits, $n_e < 1/\gamma_1$, and increased selection $w_e^2 = \gamma_1 n_e w^2$ can describe these cases qualitatively but may not completely capture the distribution of effect sizes. The value of n_e would change from 1 when $\rho^2 \to 1$ to $1/\gamma_1$ when $\rho^2 \to 0$. Note that with a large number of traits, very strong correlations among many of the traits will be necessary in order to create a large enough ρ^2 to have a significant effect on n_e (see Fig. A10).

To illustrate the effect of correlations among traits, we consider the following simple example (Fig. A10). We assume the correlation matrix $\boldsymbol{\Sigma}$ takes the form

$$\boldsymbol{\Sigma} = \begin{pmatrix} 1 & r^2 & r^2 & \\ r^2 & 1 & r^2 & \cdots \\ r^2 & r^2 & 1 & \\ & \vdots & & \ddots \end{pmatrix}, \tag{A77}$$

meaning that that all traits contribute equally to the fitness and every pair of traits has the same correlation coefficient r^2. When $r^2 = 0$ this becomes an isotropic model. When $r^2 = 1$, effect sizes are always identical for every trait; thus, this case is equivalent to having only one trait with the strength of selection increased n-fold. Intermediate cases can be approximated by finding an effective number of traits $n_e < 1/\gamma_1 = n$, such that an isotropic model with n_e and $w_e^2 = \gamma_1 n_e w^2 = w^2 n_e/n$ qualitatively describes the distribution of effect sizes. Numerical results of this model are shown in Fig. A10.

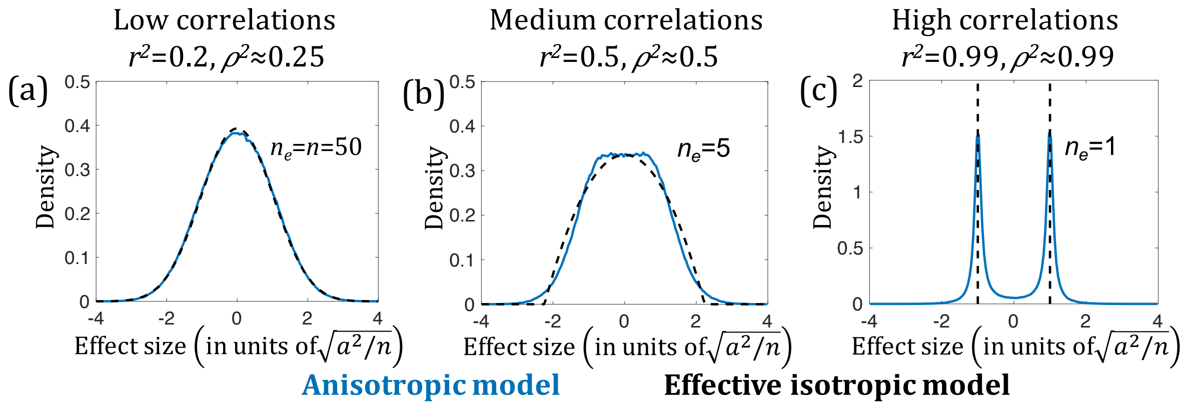

Figure A10. Effects of correlations among traits on the distribution of effect sizes. Numerical results for our model with the correlation matrix defined in Eq. A77 and $n = 50$ traits are shown in blue and the corresponding isotropic model in black dashes. (a) When correlations are low, the isotropic model approximates the distribution of effect sizes well. (b) With large correlations, we need to use an effective number of traits, in this example $n_e = 5$, and rescale selection, in this case to $w_e^2 = w^2 n_e/n = w^2/10$, in order to approximate the distribution of effect sizes. (c) When the correlations approach 1, the distribution of effect sizes becomes singular and approaches the distribution for an isotropic model with $n_e = 1$ and $w_e^2 = w^2/n = w^2/50$.

6. The power to detect loci in GWAS

In this section, we summarize the results that we rely on in connecting our theoretical predictions with the observations from GWAS (see Discussion in main text). These results provide a first approximation of the power to detect loci in GWAS in re-sequencing and genotyping studies. In Section 6.3 we consider potential complications that arise when GWAS do not identify causal loci but rather SNPs in LD with them.

6.1. Re-sequencing studies

First, we consider how the power to identify a locus in a GWAS depends on its contribution to genetic variance. To this end, we follow Sham and Purcell (24) in assuming a simplified model for a GWAS in which loci are detected using a linear regression of the phenotype against the genotype at individual loci, and the dependence of phenotype on genotype follows an additive model. The slope of the regression (the regression coefficient), which is also the estimate of the effect size, $\hat{a}_1$ is then approximately normally distributed as

$$\hat{a}_1 \sim \mathrm{N}\left(a_1, \frac{V_P/m}{2x(1-x)}\right), \tag{A78}$$

where a_1 is the true effect size and x is the minor allele frequency at the locus (which, due to the large study sizes, we assume to be estimated without error), V_P is the total phenotypic variance, and m the study size (which in reality may be an effective size reflecting study design, e.g., when the sample is split into discovery and validation panels) (24).

Under the null hypothesis, the true effect size is 0, meaning that

$$\hat{a}_{1\,\mathrm{null}} \sim \mathrm{N}\left(0, \frac{V_P/m}{2x(1-x)}\right) \tag{A79}$$

and therefore, the estimated contribution to variance has a chi-squared distribution with one degree of freedom

$$\frac{\hat{v}_{\mathrm{null}}}{V_P/m} = \frac{2\hat{a}_{1\,\mathrm{null}}^2 x(1-x)}{V_P/m} \sim \chi_1^2. \tag{A80}$$

The power to identify a locus as significant with p-value p^* is the probability that the estimated contribution of the locus to variance, $\hat{v}$, is large enough that

$$\Pr(\hat{v}_{\mathrm{null}} > \hat{v}) < p^*. \tag{A81}$$

This condition can be translated into a threshold contribution to variance v^* for which loci with $\hat{v} > v^*$ are considered significant, i.e. $\Pr(\hat{v}_{\mathrm{null}} > v^*) = p^*$, with v^* given by

$$\frac{v^*}{V_P/m} = 2\left(\text{erf}^{-1}(1-p^*)\right)^2, \tag{A82}$$

and erf denoting the error function. The power to identify a locus with a contribution v to the genetic variance is $\Pr(\hat{v} > v^*|v)$ and the distribution of $\hat{v}$ is given by

$$\frac{\hat{v}}{V_P/m} = \frac{2\hat{a}^2 x(1-x)}{V_P/m} \sim \chi_1^2\left(\frac{v}{V_P/m}\right), \tag{A83}$$

where χ_1^2 denotes a non-central chi-squared distribution with one degree of freedom. Therefore, power is given by

$$\text{H}(v, p^*) = \Pr(\hat{v} > v^*|v) = \text{h}_+\left(\frac{v}{V_P/m}, p^*\right) + \text{h}_-\left(\frac{v}{V_P/m}, p^*\right), \tag{A84}$$

where $\text{h}_\pm(y, p^*) = \frac{1}{2}\left(1 \pm \text{erf}\left(\sqrt{y/2} \mp \text{erf}^{-1}(1-p^*)\right)\right)$ and the two terms correspond to the estimated and true effect sizes having the same or opposite sign.

The form of the power function carries important implications (Eq. A84 and Fig. A11). Notably, it shows that (in this approximation) power depends only on the contribution of a locus to variance, and this contribution should be measured relative to, or in units of, V_P/m. This scale makes intuitive sense, because the total phenotypic variance generates the background noise for detecting an individual locus, and the background noise is inverse proportional to the study size. In particular, the threshold contribution to variance v^*, as defined above, is proportional to V_P/m and is also the contribution to variance at which power is 50%, i.e.,

$$\text{H}(v^*, p^*) = 1/2. \tag{A85}$$

The power function can then be approximated by a step function (see Fig. A11)

$$\text{H}(v) \approx \Theta(v - v^*) = \begin{cases} 1 & v > v^* \\ 0 & v < v^* \end{cases}. \tag{A86}$$

This will be a good approximation when the number of loci that fall at intermediate range (e.g., with power between 0.1 and 0.9) is negligible compared to the number that falls outside this range.

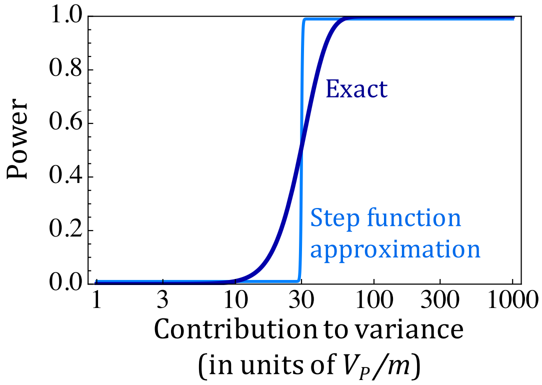

Figure A11. The power to detect loci as a function of their contribution to genetic variance (given in units of V_P/m). Shown are the exact power function (Eq. A84) and its step function approximation (Eq. A86) for $p = 5 \cdot 10^{-8}$.

Further insights come from considering this power function in conjunction with our theoretical results (Section 3). Notably, our results suggest that the first loci to be detected, those that contribute the most to variance, are intermediate and strongly selected, and that their contributions to variance are on the order of v_s. We therefore expect GWAS to begin to identify loci (and account for substantial genetic variance) when v^* is on the order of v_s, i.e., when the study size m is on the order of V_P/v_S. We would further expect the rate of increase in identifying new loci (and in accounting for genetic variance) to be similar for different traits when variance is measured in units of v_S.

6.2. Genotyping

Most current GWAS rely on genotyping instead of re-sequencing, resulting in an additional loss of power (26). Specifically, these studies impute the alleles at loci that are not included in the genotyping platform (27), and the imputation becomes imprecise when the imputed alleles are rare (Fig. A12). If causal loci with rare minor alleles are included in GWAS, this imprecision leads to an under-estimation of their effect size, resulting in loss of power (26). For loci with MAF x and effect size a, the expected estimate of the effect size would be reduced by a factor of $r(x)$, where $r^2(x)$ is the mean correlation between the imputed and real alleles (28), and the distribution of estimates can be approximated by

$$\hat{a}_1 \sim \mathrm{N}\left(ra_1, \frac{V_P/m}{2x(1-x)}\right). \quad \text{(A87)}$$

Employing the reasoning of the previous subsection, we can therefore approximate the power to detect a locus by $\mathrm{H}(r^2 v, p^*)$, where H is the power function defined in Eq. A84.

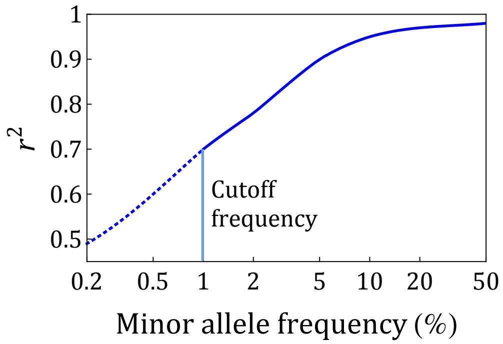

Figure A12. The precision of imputation decreases with MAF. Specifically we show the mean correlation between imputed and real genotypes as function of minor allele frequency, for a study using an Illumina 1M SNP array and the 1000 genomes phase III as an imputation panel (based on Extended Fig. 9A in (29)). We approximate the effect on power by excluding loci with MAF < 1% and assuming that loci with greater MAFs are imputed correctly.

In practice, GWAS often include only loci with MAF above a threshold, which is chosen to ensure precise imputation. We therefore approximate the effect of genotyping on power by excluding loci below a threshold MAF and assume that loci that exceed this threshold are imputed correctly.

6.3. Tagging

Our inference is predicated on the assumption that the distribution of estimated variances among genome-wide significant (GWS) associations faithfully reflects the distribution among causal loci. We have no obvious alternative but to make this assumption, and arguably, the good fit of our theoretical predictions to the distribution of variances among associations provides some support for this assumption. While this assumption cannot be directly tested at present, existing arguments and evidence suggest that it is plausible, for reasons that we briefly review.

Most of the variants discovered by GWAS are common. Specifically, all but one of the GWS associations for height and BMI, which we rely upon in our inference, have MAF>1%, and the MAF of most associations is considerably greater. In considering the validity of our assumption, we therefore consider what could be tagged by such common associations. One possibility is that a given common association is tagging a single common, causal variant. Given the accuracy of imputation for common variants (see Fig. A12), we would therefore expect that the tagging variant would be in almost perfect LD with the causal one (including the possibility that the association is actually with the causal variant). If that were the case, then we would expect the estimated frequency and effect size, and thus the estimated contribution to genetic variance, to be very similar to those of the causal variant. A second possibility is that a given association tags several common causal variants within the same genomic region. The number of causal variants would likely be small, as otherwise the tagging allele is highly unlikely to be in LD with causal alleles that affect the trait in the same direction. If that were the case, given the accuracy of imputation of the causal alleles, we would expect conditional analysis (e.g., 30) to successfully distinguish between the different causal variants, thus returning us to the previous scenario.

A third possibility involves a common association tagging rare, causal variants (25). While a single rare, causal variant would have to have an unreasonably large effect size in order to result in a common GWS association (31), it has been argued that several rare, causal

variants in the same genomic region may be tagged by a single “synthetic association” (25). In this case, the relatively low LD between the association and each of the causal variants would imply that the estimated contribution to variance of the association would have to be much smaller than the combined contribution of the causal variants (25, 31). If this were the case for many associations identified in GWAS, it would violate the premise of our inference.

However, multiple lines of evidence suggest that it is not a common occurrence. One is that, where data is available, associations often replicate across populations. For example, there is considerable overlap between GWS associations for height in Europeans and East-Asians (32). While we would not expect perfect replication even if associations were tagging single, common, causal variant, we would expect practically none if they were synthetic, both because the underlying rare, causal alleles would be less likely to be shared among populations and because the particular LD configuration that allows for their tagging in one population would likely break down in others (33, 34). A second is that simulation studies suggest that synthetic associations are expected to have much lower MAF than typically observed among associations in GWAS (31). Moreover, these simulations suggest that, because synthetic association should capture only a fraction of the variance contributed by the tagged loci, having many synthetic associations would imply there being much more heritable variance than is known to be present in the population. A third, and perhaps most direct line of evidence, is that, to the best of our knowledge, none of the studies that pursued fine-mapping around GWS associations have uncovered such synthetic associations (33, 35, 36). These arguments, together with other lines of evidence (e.g., 31) suggest that in practice synthetic associations are likely to be rare.

Perhaps a more plausible alternative is for an association to primarily tag one common, causal variant, with which it is in high LD, but also to pick up the effects of one or a few rare, causal variants, which are more poorly tagged. Under this scenario, we might expect the estimated contribution to variance to slightly overestimate the contribution of the dominant causal variant. To the best of our knowledge, this scenario has not been well characterized, making it difficult to assess how common it is or whether the overestimation would be substantial.

In summary, given what we now know, our assumption about the distribution of estimated variances among associations reflecting the distribution among causal loci seems sensible.

7. Inference

In this section, we describe how we used our model to make inferences based on GWAS results for height and body mass index (BMI). As we note in the Discussion, these inferences are meant as an illustration and do not incorporate the effects of demography and a few other factors (e.g., genotyping and errors in the estimation of effect sizes (24, 26)), which lie beyond the scope of this study.

7.1. The composite likelihood

Our inferences are based on a composite-likelihood approach. We begin by describing the composite-likelihood function and its maximization, when the loci detected by GWAS are strongly selected and can be described by the high-pleiotropy limit. In this case, we have shown that the distribution of variances among loci is insensitive to the distribution of selection coefficients, depends on a single parameter v_s, and is well approximated by the probability density

$$\rho(v) = \frac{2\exp\left(-2\sqrt{v/v_s}\right)}{v} \qquad \text{(A88)}$$

(Section 3.2). Further approximating the power in GWAS as a step function (see Section 6), we find that the probability density of sites that exceed a threshold v^* can be approximated by

$$\mathrm{f}(v|v_s, v^*) = \frac{\rho(v)}{\int_{v>v^*}\rho(v)} = \frac{\exp\left(-2\sqrt{v/v_s}\right)}{2v\ \mathrm{I}\left(2\sqrt{v^*/v_s}\right)}, \qquad \text{(A89)}$$

where $\mathrm{I}(x) \equiv \int_{t>x}\exp(-t)/t$ (see Eq. A37). We therefore approximate the log-composite-likelihood of v_s given the contributions to variance of the K loci detected in a GWAS, $\{v_i\}_{i=1}^K$, by

$$\mathrm{LCL}\left(v_s\middle|\{v_i\}_{i=1}^K, v^*\right) = \sum_{i=1}^{K}\log\left(\mathrm{f}(v_i|v_s)\right) =$$

$$= -\left(2/\sqrt{v_s}\right)\sum_{i=1}^K\sqrt{v_i} - K\log\left(\mathrm{I}\left(2\sqrt{v^*/v_s}\right)\right) - \sum_{i=1}^K\log\left(\sqrt{v_i}\right). \qquad \text{(A90)}$$

It follows that the composite-likelihood is maximized when

$$\hat{v}_s = \mathrm{argmin}_{v_s}\left\{2\overline{\sqrt{v}}/\sqrt{v_s} + \log\left(\mathrm{I}\left(2\sqrt{v^*/v_s}\right)\right)\right\}, \qquad \text{(A91)}$$

where $\overline{\sqrt{v}} \equiv \frac{1}{K}\sum_{i=1}^K\sqrt{v_i}$.

We also consider the models without pleiotropy and in which the degree of pleiotropy is a parameter. In the case without pleiotropy,

$$\rho(v) = \frac{2\exp(-2v/v_s)}{v} \tag{A92}$$

(see Section 3.2). By following the same steps, we find that the composite-likelihood is then maximized when

$$\hat{v}_s = \text{argmin}_{v_s}\{2\bar{v}/v_s + \log(\text{I}(2\,v^*/v_s))\}, \tag{A93}$$

where $\bar{v} \equiv \frac{1}{K}\sum_{i=1}^{K} v_i$. When the degree of pleiotropy n is a parameter of the model, we find that

$$\rho_n(v) = \int_{a_1} \frac{2}{v} exp\left(-\frac{2v/v_s}{a_1^2/(a^2/n)}\right) \varphi_n(a_1|a) \tag{A94}$$

(see Section 3.2). Again, following the same steps, we find that the probability density of sites that exceed a threshold v^* is

$$\text{f}_n(v|v_s) = \frac{\rho_n(v)}{\int_{v>v^*} \rho_n(v)} \tag{A95}$$

and the log-composite-likelihood is

$$\text{LCL}(v_s, n\big|\{v_i\}_{i=1}^{K}, v^*) = \sum_{i=1}^{K} \log(\rho_n(v_i)) - K\log\left(\int_{v>v^*} \rho_n(v)\right). \tag{A96}$$

In the latter case, we used numerical maximization to show that the composite-likelihood estimates for height and BMI converge to the high pleiotropy limit. Specifically, we maximized the composite-likelihood specifying an interval of [1,1000] for n, where for both traits the estimates converged to the upper limit of 1000. While numerical optimization does not allow us to specify an infinite interval, the likelihood function and maximal value for n=1000 are indistinguishable from those in the high-pleiotropy limit.

7.2. Determining v^* and removing outliers

Our likelihood maximization requires us to specify the value of the threshold v^*. We choose this threshold based on the empirical distributions of the contributions to variance among genome-wide significant associations (Fig. A13a & b). Specifically, when the contributions to variance approach the lower boundary for discovery, we observe a decline in the density of loci. This is likely due to a gradual reduction in power and suggests that our approximation for power (as a step function) breaks down for these values of variance. We therefore choose thresholds that appear to be above this decline ($v^* = 1.4 \cdot 10^{-4} V_P$ for height and $v^* = 1.35 \cdot 10^{-4} V_P$ for BMI; Fig. A13a & b), resulting in the removal of 53 loci for height and 11 for BMI. We also examine how our estimates of v_s depend on the choice of v^*, and find that they are much more sensitive to reducing the threshold than to increasing it; in fact, the estimates we obtain by increasing the threshold are within the confidence

intervals of the estimate with the chosen thresholds (Fig. A13c & d). This analysis further supports our choice to exclude the loci with the lowest contribution to variance. For BMI, we also dropped the locus with the largest contribution to variance (near FTO), which appears to be an outlier (Fig. A13b) and has been suggested to be under balancing selection (37).

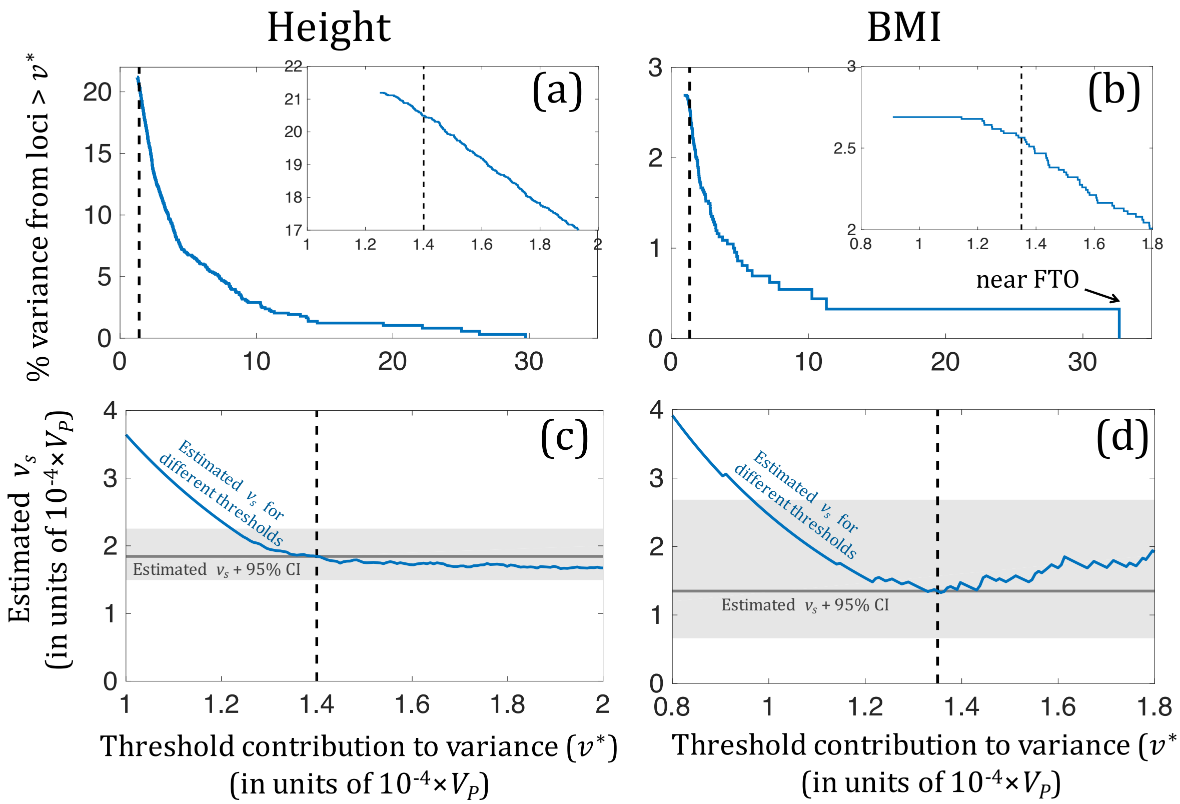

Figure A13. Determining v^* and removing outliers. The total variance from significant associations as a function of the threshold contribution to variance, for height (a) and BMI (b). The insets show a close up of the lower range of contributions to variance, highlighting the decline in the density of discovered loci. Our chosen thresholds are shown by the dashed vertical line (in all graphs). Our estimates of v_s as a function of the chosen threshold, for height (c) and BMI (d). When we increase the threshold, the estimates remain within the 95% CI of the estimate with our chosen threshold.

7.3. Estimating target size and explained variance

We estimate the target size and the variance explained, both for varying study size and total, based on our estimates of v_s. The population-scaled mutational input per generation from strongly selected loci, $2NU_s$, is estimated by

$$\widehat{2NU}_s = K / \int_{v>v^*} \rho(v|\hat{v}_s), \quad \text{(A97)}$$

(see Eq. A38) and the corresponding estimate for the target size is

$$\hat{L}_s = \widehat{2NU}_s / \widehat{2Nu}, \quad \text{(A98)}$$

where the estimate for the population scaled mutation rate per site per generation $\widehat{2Nu} \approx 0.5 \cdot 10^{-3}$ is based on heterozygosity (29). The explained variance corresponding to GWAS with study size m is estimated by

$$\hat{\sigma}_s^2(m) = \widehat{2NU}_s \int_{v>v^*(m)} v\rho(v|\hat{v}_s) = K\left(\int_{v>v^*(m)} v\rho(v|\hat{v}_s) / \int_{v>v^*(m_0)} \rho(v|\hat{v}_s)\right), \quad \text{(A99)}$$

where we approximate the threshold corresponding to study size m based on the study size, m_0, and threshold, v^*, in current GWAS, by

$$v^*(m) = v^* \cdot (m_0/m). \quad \text{(A100)}$$

To estimate the total variance arising from strongly selected loci, we simply set the threshold in Eq. A99 to 0.

7.4. Estimating confidence intervals

We use a combination of non-parametric and parametric bootstrap to estimate confidence intervals (CI). We use non-parametric bootstrap to estimate the CI for the model parameters v_s and L_s: specifically, we perform 10,000 iterations, in which we resample the loci identified by GWAS and repeat the estimation of v_s. We use parametric bootstrap to estimate the confidence intervals in Fig. 5a, describing the explained variance as a function of threshold based on our model. To that end, we rely on our model with the point estimates for v_s and L_S, to generate 10,000 samples from GWAS with the specified threshold, and then calculate the total variance explained by these samples. We use a combination of non-parametric and parametric bootstrap to calculate the CI for model predictions, including the total variance, σ_s^2, and the explained variance, $\sigma_s^2(m)$, and number of loci as a function of study size (Fig. 5b & c). In this case, we generate 10,000 samples by: i) estimating v_s based on a resampled set of GWAS loci (similar to the non-parametric procedure), and ii) using the estimated v_s and corresponding L_s to generate a GWAS hits above v^* based on our model (similar to the parametric procedure); we then calculate the appropriate summary based on the latter samples. This two stage procedure is intended to capture the uncertainty generated by both the errors in estimating our basic model parameters and the noise generated by the stochastic processes underlying the

number and variance at segregating loci that are yet to be discovered. The resulting estimates and CI are summarized in Table A2.

Parameter		Height	BMI
Contribution to variance per strongly selected locus, in units of the total phenotypic variance	$\hat{v}_s/V_P$	1.8 [1.5, 2.3]×10⁻⁴	1 [0.6, 1.7] ×10⁻⁴
Expected study size required to capture 50% of the strongly selected variance	$m_{50\%}$ $(\approx 43V_P/\hat{v}_s)$	230 [190, 290] K	420 [250, 770] K
Number of newly arising strongly selected mutations per generation in the population	$2NU_s$	2300 [1800, 3000]	600 [300, 1900]
Mutational target size for strongly selected mutations	L_s	4.6 [3.6, 6.0] Mbp	1.3 [0.6,3.8] Mbp
% contribution to phenotypic variance from strongly selected loci	σ_s^2/V_P	42 [39, 45] %	7 [5, 10] %
Proportion of heritability from strongly selected loci	σ_s^2/V_G $(= \sigma_s^2/h^2V_P)$	53 [49, 57] %	13 [10, 21] %

Table A2. Parameter estimates and their confidence intervals for height and BMI based on GWAS results; the heritability was assumed to be 0.8 for height and 0.5 for BMI (8, 10).

7.5. Testing goodness of fit

We use the Kolmogorov-Smirnov D statistic (38, 39) to test the goodness of fit of our models without pleiotropy and in the high pleiotropy limit. Since our parameter estimates are inferred from the data that we are testing against, we cannot rely on the standard tables for the p-values. We therefore generate null distributions for the D statistic using parametric bootstrap based on our models. Specifically: i) we generate 10^5 samples of K significant loci based on the model under consideration, with the corresponding estimate of v_s, ii) we infer v_s based on each sample, and iii) calculate the Kolmogorov-Smirnov D statistic between the distribution of variances for the K loci in each sample and the corresponding theoretical distribution based on the v_s inferred from that sample. The

resulting distribution of D statistics corresponds to our null hypothesis, i.e., that the loci detected in GWAS arose according to our model, and specifically to the way we calculate the D statistic between the observed distribution of variances for the K detected loci and the theoretical distribution that we inferred based on these observations. We then calculate the D statistic, D_r, based on the real data and corresponding theoretical distribution, and estimate the one-sided p-value by

$$\hat{p}_{K-S} = \frac{\#\text{ simulated datasets with } D>D_r}{\#\text{ simulated datasets}}. \quad \text{(A101)}$$

Note that unlike the common case, here the inability to reject the null indicates that the data is consistent with our model.

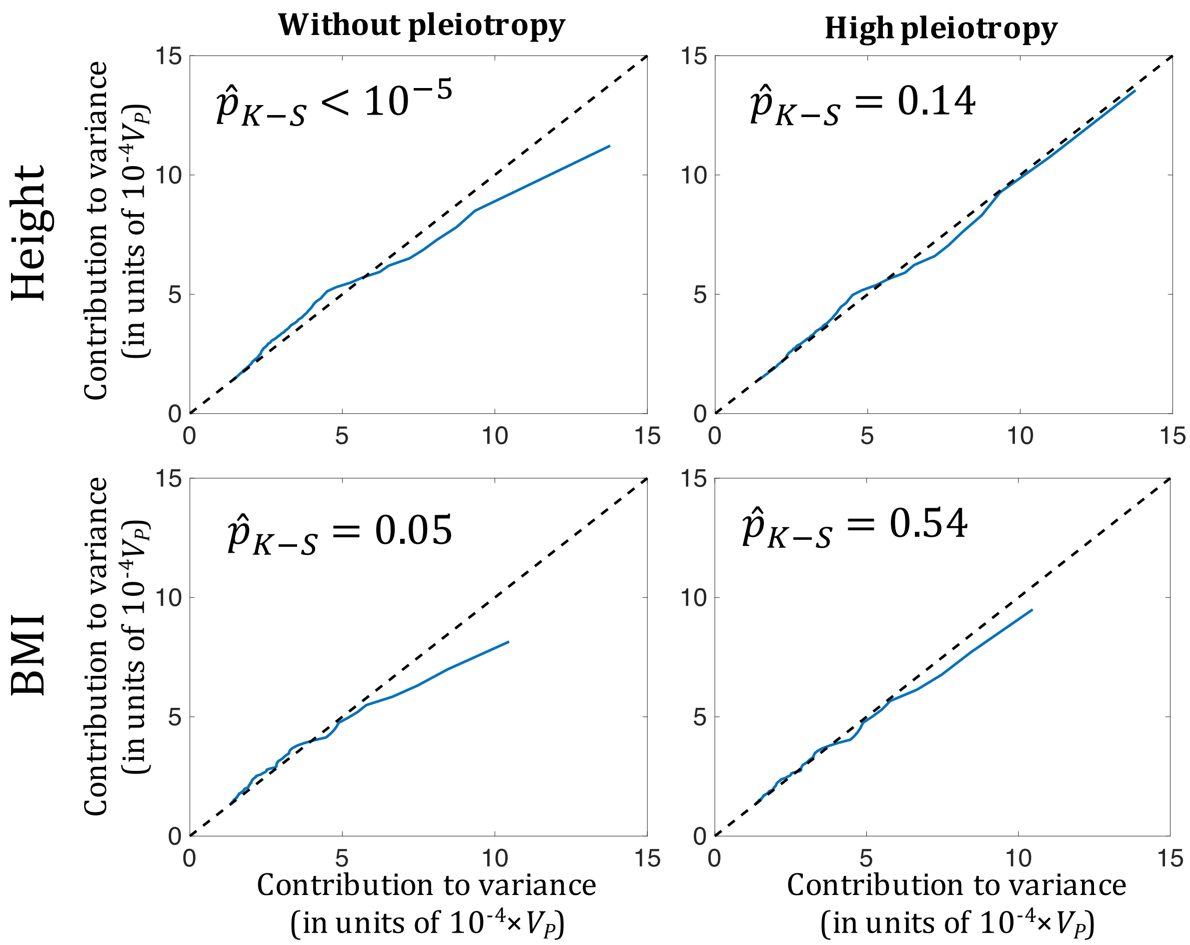

Figure A14. Q-Q plots comparing the distribution of variances among significant loci taken from the GWAS of height (10) and BMI (8) with the theoretical distributions inferred from these data, based on the models without pleiotropy (a) and in the high pleiotropy limit (b). These plots show that the model assuming high pleiotropy cannot be rejected for either trait and fits these data much better than the model without pleiotropy.

8. Consistency with other datasets and analyses

Here, we show that the results of our inference for height are consistent with findings of a recent GWAS based on exome genotyping; that our inferences for height and BMI are consistent with estimates of the heritability tagged by SNPs with MAF $> 1\%$ in the GWAS we used; and that our model is consistent with estimates about the relationship between effect size and MAF in these and other GWAS.

8.1. Exome association study of height

Marouli et al. (40) present an association study for height that was specifically designed to capture rare, exonic variants. They rely on the ExomeChip genotyping array (41), which includes the vast majority of protein-altering variants with MAF>0.1%, allowing them to directly (i.e., without imputation) test for associations among rare variants. Using a study size of more than 300,000 European individuals, they find over 400 genome-wide significant associations. Here we examine whether their findings are consistent with our inference based on the Wood et al. genome-wide, genotyping based GWAS for height (10).

In addition to protein altering variants, the ExomeChip includes some synonymous SNPs and ancestry informative markers, as well as all of the genome-wide significant associations listed in NHGRI from 2011. To avoid ascertainment biases, we consider only protein-altering variants, including non-synonymous, splice region, splice acceptor and stop codon variants. This leaves us with 250 of the Marouli et al. genome-wide significant associations. In addition, we apply the procedure described in Section 7.2, resulting in the removal of associations with contributions to variance below $v_E^* = 1.15 \cdot 10^{-4}\ V_P$, for which power is substantially diminished (Fig. A15a) ; this step leaves us with 147 associations. Next, we compare the distribution of variances among the remaining 147 associations with our theoretical prediction, with the v_S inferred from the Wood et al. data (Table A2) above the threshold v_E^* (Fig. A15b). We do not consider the fit to the number of associations, because it depends on the mutational target size for protein-altering variants affecting height, which is unknown.

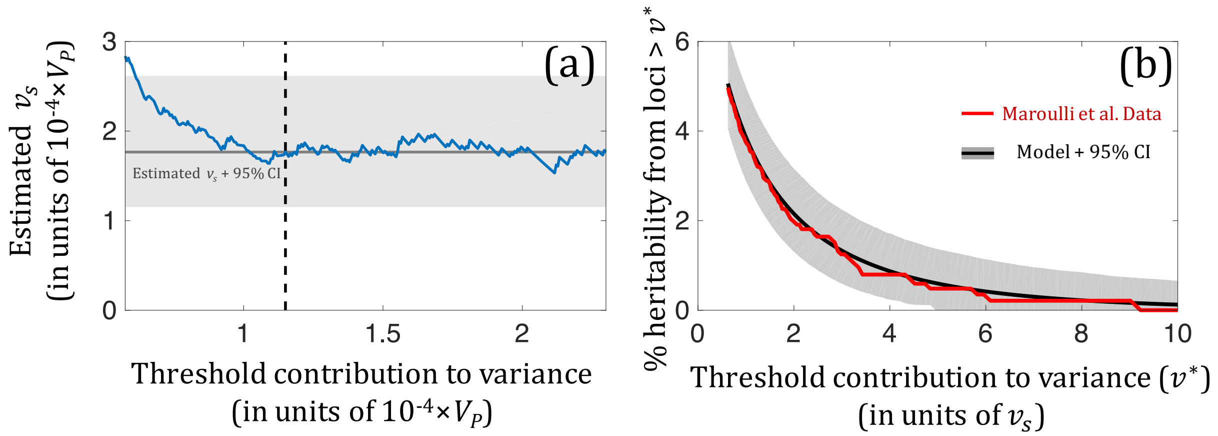

Figure A15. Comparing our inferences for height with the results of the Marouli et al. GWAS. (a) Choosing the threshold contribution to variance, v_{E}^*, above which our approximation for power applies; see Section 7.2 for details. (b) Comparing the predicted and observed distribution of variances above the threshold v_{E}^*. 95% CIs for our predictions are based on bootstrap; see Section 7.4 for details.

To test whether the observed distribution is consistent with our prediction, we calculate the Kolmogorov-Smirnov D statistic (38, 39) for this comparison, D_r, and ask whether we can reject our prediction based on the value of D_r. In approximating the null distribution of the D statistic, we must consider that: i) Some of the Marouli et al. associations might have been tagged by the genome-wide significant associations in Wood et al., which we relied upon in estimating v_S; this would lead to smaller values of the D statistic than if the two sets of associations were independent. ii) Our estimate of v_S includes some statistical error, due to the finite set of associations on which it relies. To account for these factors, we employ a parametric bootstrap procedure that mimics how the value of the D statistic arises, under the conservative scenario in which any of the associations from Marouli et al. could have been included in the data that we used in our inference. Specifically, we assume that the distribution of variances among loci follows the theoretical prediction with our estimate of v_S, and i) We sample 147 associations from the predicted distribution with threshold v_E^*, corresponding to the Marouli et al. associations. ii) Given the number, k, of these associations that fall above the threshold of the Wood et al. GWAS, $v_G^* = 1.4 \cdot 10^{-4}\ V_P$ (Section 7.2), we sample an additional $644 - k$ variants from the predicted distribution with threshold v_G^*. The resulting 644 simulated associations that fall above v_G^* correspond to the Wood et al. associations. iii) We infer $\hat{v}_S$ based on these 644 variants, thus mimicking our inference procedure, and calculate the D statistic for our predicted distribution with $\hat{v}_S$ and the distribution based on the 147 simulated variants. iv) We repeat this procedure 10^5

times to approximate the distribution of D statistic under our null, and estimate the one-sided p-value by

$$\hat{p}_{K-S} = \frac{\text{\# simulated datasets with } D > D_r}{\text{\# simulated datasets}}. \tag{A102}$$

Doing so, we find that $\hat{p}_{K-S} = 0.99$, and thus, we cannot reject our predictions based on the data from Marouli et al. (40). This result indicates a good fit to their findings.

8.2. The heritability arising from common SNPs

Yang et al. (42, 43) estimate the heritability that is tagged by common SNPs (MAF>1%) in GWAS of several traits, including height and BMI. Here we ask whether their estimates are consistent with our inferences based on genome-wide significant (GWS) associations from the same GWAS.

First, we consider our inferences predicated on equilibrium demography. On this assumption, we predict that GWS associations would be under intermediate or strong selection, roughly corresponding to $S > 5$. Our estimates then suggest what proportion of variance arises from loci under this range of selection effects, where the rest of the variance is assumed to arise from loci under weaker selection. The proportion of variance that arises from sites with $S < 5$ and MAF $> 1\%$, $P_w(> 1\%)$, can be bound from above by the variance that would arise if they were all effectively neutral, $P_n(> 1\%)$. Further denoting the proportion of variance that arises from sites with $S > 5$ and MAF $> 1\%$ by $P_s(> 1\%)$, and the overall proportion of variance from sites with MAF $> 1\%$ by $P(> 1\%)$, we obtain the following requirement:

$$P_s(> 1\%) = P(> 1\%) - P_w(> 1\%) \geq P(> 1\%) - P_n(> 1\%). \tag{A103}$$

For height, Yang et al. estimate that $P(> 1\%) = 0.59$ (42), and our estimate for $P_n(> 1\%) = 0.45$. As Fig. A16 shows, so long as most of the estimated variance with $S > 5$ (53%) arises from loci with $S < 135$, the requirement in Eq. A103 will be easily met. For BMI, Yang et al. estimate that $P(> 1\%) = 0.5$ (42), and our estimate for $P_n(> 1\%) = 0.83$. The lower bound in Eq. A103 is therefore negative, implying that requirement A103 is met regardless of the distribution of selection coefficients for $S > 5$.

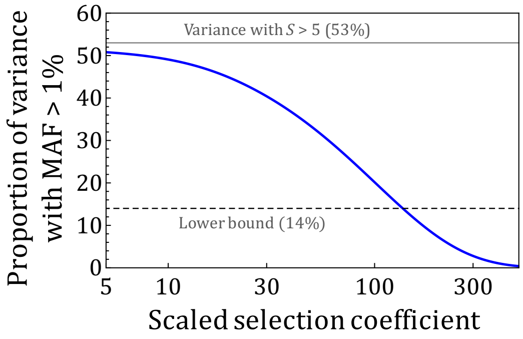

Figure A16. The Yang et al. (42, 43) estimate of the genetic variance in height arising from loci with $MAF > 1\%$ imposes weak constraints on the distribution of selection coefficients, assuming our estimate for the genetic variance with $S > 5$.

Next, we consider the results of our analysis in Section 9, incorporating the effects of recent changes in European population size. Our results suggest that GWS associations arise from loci with selection coefficients of $s \approx 10^{-3}$. We therefore ask whether the Yang et al. (42, 43) estimates are consistent with ours, when we attribute our equilibrium estimates of the proportion of variance arising from intermediate and strongly selected loci to selection coefficients of $s \approx 10^{-3}$, assuming that the remaining variance arises from loci under weaker or stronger selection (a more rigorous approach would be to account for demography in estimating the proportion of variance, but this extension lies beyond the scope of the current paper). The proportion of variance arising from sites under weaker selection with MAF $> 1\%$ is bound from above by $P_n(> 1\%)$, whereas the corresponding proportion from sites under stronger selection can be vanishingly small. Denoting the proportion of variance arising from sites with $s \approx 10^{-3}$ and MAF $> 1\%$ by $P_{10^{-3}}(> 1\%)$, we therefore obtain the following condition:

$$P(> 1\%) \geq P_{10^{-3}}(> 1\%) \geq P(> 1\%) - P_n(> 1\%). \quad \text{(A104)}$$

If we assume the Yang et al. (42) estimates for $P(> 1\%)$ and our estimates for $P_{10^{-3}}(> 1\%)$, Table A3 shows that this requirement is easily met for both height and BMI. More generally, our analysis illustrates that heritability estimates of this kind impose rather weak constraints on our inferences.

	$P(> 1\%)$		$P_{10^{-3}}(> 1\%)$		$P(> 1\%) - P_n(> 1\%)$
Height	0.59	≥	0.48	≥	0.59−0.38=0.21
BMI	0.5	≥	0.12	≥	0.5−0.83=−0.33

Table A3. Consistency between the Yang et al. (42) estimates of the total variance arising from loci with MAF $> 1\%$ and our estimates of the variance arising from sites with $s \approx 10^{-3}$ and MAF $> 1\%$.

8.3. The relationship between SNP heterozygosity and effect size

More recent studies of the heritability tagged by SNPs in GWAS also make inferences about the relationship between effect sizes and MAF (44-46). Specifically, they assume that the relationship between the contribution of a site to variance, $v = 2a_1^2 x(1-x)$, and its MAF, x, takes the form

$$\mathrm{E}(v|x) \propto \big(x(1-x)\big)^{\alpha+1}, \tag{A105}$$

or equivalently, that

$$\mathrm{E}(a_1^2|x) \propto \big(x(1-x)\big)^{\alpha}, \tag{A106}$$

and they estimate the value of α from the data.

Provided a distribution of selection coefficients, $\mathrm{f}(S)$, Eq. A20 implies that in our model

$$\mathrm{E}(a_1^2|x) = \frac{\int_{a_1} a_1^2 \rho(x,a_1)}{\int_{a_1} \rho(x,a_1)} = \frac{\int_S \int_{a_1} a_1^2 \mathrm{f}(S)\tau(x|S)\eta(a_1|S)}{\int_S \int_{a_1} \mathrm{f}(S)\tau(x|S)\eta(a_1|S)}$$

$$= \frac{2w^2}{nN} \cdot \frac{\int_S S\, \mathrm{f}(S)\tau(\mathrm{x}|\mathrm{S})}{\int_S \mathrm{f}(S)\tau(\mathrm{x}|\mathrm{S})} = \frac{2w^2}{nN} \cdot \mathrm{E}(S|x). \tag{A107}$$

Thus, in our model, assuming the relationship of Eq. A105 (or A106) would imply that

$$\mathrm{E}(S|x) \propto \big(x(1-x)\big)^{\alpha} \tag{A108}$$

(See (45) for a similar derivation).

The aforementioned studies assume the relationship in Eq. A105 (or A106), without providing any evidence that this somewhat arbitrary functional form fits the data better than others, and show that values of α between -1 and 0 provide the best fit to data from GWAS of a variety of traits. To show that our model is in agreement with theirs, all we therefore need to do is to find distributions of selection coefficients, $\mathrm{f}(S)$, that approximate the relationship of Eq. A108 for values of α between -1 and 0. In Fig. A17, we assume that selection coefficients follow a Gamma distribution, where we vary its expectation and variance. As expected, $\mathrm{E}(S|x)$ monotonically decreases as x increases. When $\mathrm{E}(S) \ll 1$ or the coefficient of variation $\mathrm{C_V^2}(S) \ll 1$, $\mathrm{E}(S|x)$ varies minimally with x and can approximated by Eq. A108 with $\alpha = 0$. In other cases, $E(S|x)$ varies more substantially with x. When we approximate those cases using Eq. A108, we obtain a range of α values between -1 and 0. Thus, our model appears to be consistent with the values of α reported in (44-46). Our inferences for height and BMI are not very informative about the distribution of selection coefficients and are therefore not comparable with estimates of α.

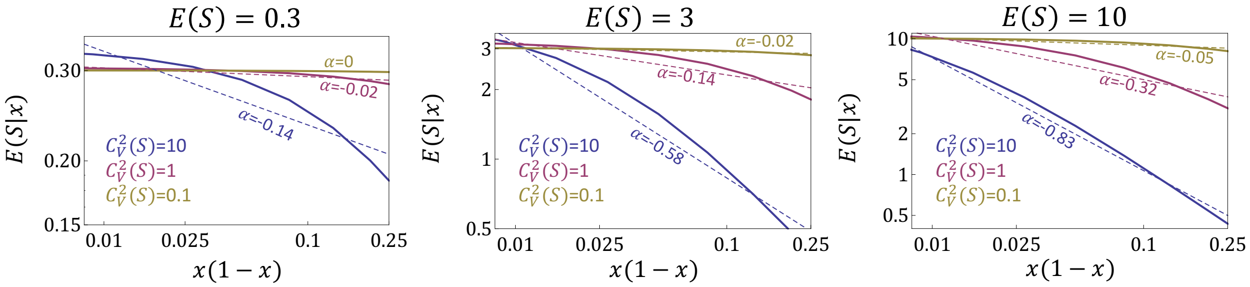

Figure A17. The relationship between effect size, or equivalently, selection coefficient, and MAF, shown on a log-log scale. Selection coefficients are gamma-distributed, with $\mathrm{E}(S) = 0.3, 3, 10$ and shape parameters $k = 0.1, 1, 10$. $\mathrm{E}(S|x)$ was approximated using the functional form $\mathrm{E}(S|x) \propto \left(x(1-x)\right)^{\alpha}$ (Eq. A108), by taking the values of $\log\left(\mathrm{E}(S|x)\right)$ and $\log\left(x(1-x)\right)$ on a grid of x values, $x = 0.5 \cdot 10^{-i/4}$ with $i = -8, -7, \dots, 0$, and preforming least-square linear regression.

9. The effects of demographic history

While our theoretical results were derived on the assumption of a panmictic population of constant size, the evolutionary history of human populations sharply deviates from these simplifying assumptions. Notably, most large GWAS, including the studies of height (10) and BMI (8) that we use to test our predictions, have been performed in predominantly European populations, which are known to have experienced dramatic changes in their effective population size, including an Out-of-Africa bottleneck about ~100 KYA and explosive population growth over the past ~5 KY (47-50). These changes in population size have dramatically impacted the frequencies of neutral and selected alleles (47-49, 51-53), and are therefore expected to have had a substantial impact on the architecture of quantitative traits (52, 53). These considerations raise several questions about the interpretation of the fit between our predictions and GWAS data. Notably, how will these historical changes in population size affect our prediction, and specifically, why do our equilibrium predictions fit GWAS data despite the dramatic historical changes in population size? While a comprehensive treatment of these questions warrants a study in itself, we briefly address them here.

Even with changing population size, our results for the dynamics at segregating sites should still hold. Notably, we would expect the mean phenotype in the population to maintain the optimal phenotypic value, because any displacement from the optimum would be quickly adjusted by small changes to allele frequencies at numerous loci (see Section 4.4). As a result, the dynamics at individual sites would be decoupled, and well approximated by the first two moments of change in allele frequency described in Eqs. 5 and 6, which appear in the Results section of the main text. In particular, the first moment would correspond to under-dominant selection, and the selection coefficient would be proportional to the size of the allele in the n-dimensional trait space (as described in Eq. 7, which appears in the Results section of the main text). We can therefore study the effect of historical changes in population size on allele frequencies with simulations, using a fixed (not population-scaled) selection coefficient with under-dominance, and having the population size change over time.

To this end, we modify the simulation from Simons et al. (53) to incorporate under-dominance, and the historical changes in the effective population size of European populations inferred by Schiffels and Durbin (50) (Fig. A18). In brief, we simulate a bi-

allelic site in a diploid, panmictic population, in which mutations, with selection coefficient s, arise at rate $u = 1.25 \cdot 10^{-8}$ per bp per generation (5, 50), and the next generation derives from Wright-Fisher sampling and fecundity selection. The simulation begins 150K generations ago (corresponding to 4.5 MYA with a generation time of 30 Y, as assumed by (50)), with a burn-in period with a constant population size of 14,448. In accordance with the Schiffels and Durbin inferences (50), changes in population size begin 55,940 generations ago (corresponding to 1.7 MYA). Specifically, we piece together the MSMC inferences from two and four haplotypes of European individuals (CEU) from HapMap project (54), where the four haplotype MSMC captures the bottleneck and recent growth and is used for times <170 KYA, and the two haplotype MSMC captures more ancient times and is used for times >170 kya (see Fig. A18). The derived allele frequency is recorded at the last generation corresponding to the present. The software and documentation can be found at https://github.com/sellalab/GenArchitecture.

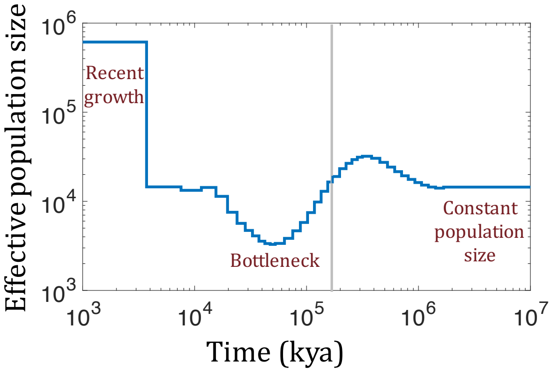

Figure A18. Changes in population size in the history of Europeans, as inferred by Schiffels and Durbin using MSMC (50). The cutoff between the two and four haplotype MSMC inferences is marked by the gray line.

We rely on such simulations to study how changes in populations size will affect the genetic architecture of a trait under the assumptions of our model. To this end, we consider a grid of selection coefficients: $s = 10^{-i/8}, i = 8, 9 \dots, 40$, where for each selection coefficient, we run $15 \cdot 10^6$ simulations. In this way, we obtain numerical approximations for the expected site frequency spectrum corresponding to each selection coefficient, which replaces the term $2Nu \cdot \tau(x|S)$ in our expressions for summaries of genetic architecture (Section 3). We further assume the high pleiotropy limit form for the distribution of effect sizes on the focal trait corresponding to a given selection coefficient (i.e., Eq. 11, which appears in the Results section of the main text).

We first consider how demography affects the distribution of genetic variances among sites with different selection coefficients (Fig. A19a). The expected contribution per site (including both sites that are segregating and monomorphic) peaks around a selection coefficient of $s \approx 10^{-3}$ and, as in the case with constant population size (Fig. 2a), when the strength of selection increases, it appears to approach a plateau (Fig. A19a). The distribution of variances among sites, however, is dramatically affected by changes in population size: for selection coefficients around $s \approx 10^{-3}$, a much greater proportion of variance comes from sites with large contributions than from those with both weaker and stronger selection coefficients (Fig. A19b). This behavior contrasts with the case of a constant population size, where for sufficiently strong selection ($S > 5$), the distribution of variances among sites is insensitive to the strength of selection (see Fig. 3b).

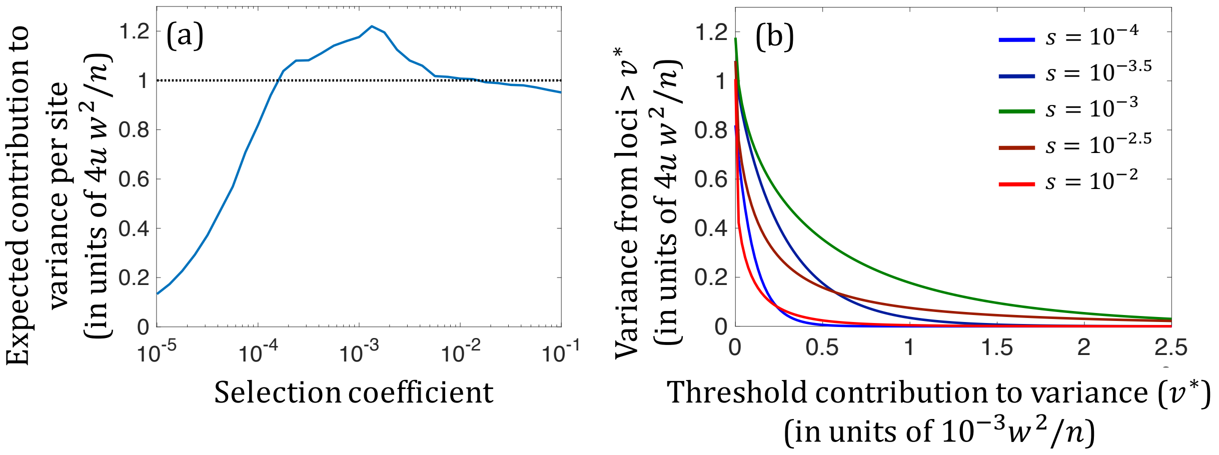

Figure A19. The joint effects of selection and changes in populations size (as inferred for Europeans by Schiffels and Durbin (50)) on the distribution of genetic variances among sites. (a) The expected contribution to variance per site, both segregating and monomorphic, as a function of the (unscaled) selection coefficient. Variance is measured in units of $4u\,w^2/n$, the equilibrium expectation for a strongly selected site. (b) The cumulative variance arising from sites with contributions above a threshold (y-axis) as a function of the threshold (x-axis); cumulative variance is measured in units of $4u\,w^2/n$, while the threshold in units of $10^{-3}w^2/n$.

As we establish below, these findings can be understood as follows. The segregating sites with the largest contribution to current genetic variance are due to mutations with $s \approx 10^{-3}$ that arose shortly before or during the Out-of-Africa bottleneck. Such mutations were under strong selection (i.e., with $2N_e s \approx 50$) before the bottleneck, but with the drop

to an effective population size of $N_e \approx 4000$ during the bottleneck, they experienced more relaxed selection (with $2N_e s \approx 10$), allowing some of them to ascend to higher frequencies. The durations of subsequent increases in population size, and of explosive growth in particular, were too short to allow for a substantial reduction in their frequencies (e.g., a mutation with $s = 10^{-3}$ that reached 20% frequency by the end of the bottleneck, 15 Kya, would have an expected frequency of 18% at present). As a result, these mutations would have large contributions to variance at present. Moreover, their site frequency spectrum and distribution of contributions to variance are well approximated by assuming a population size of $N_e \approx 5000$ – roughly the geometric mean of populations sizes from the beginning of the bottleneck to the present – and thus to scaled selection coefficients of $2N_e s \approx 10$.

Extant segregating mutations under substantially stronger selection are expected to be much younger. They therefore tend to have arisen after the bottleneck, when the population size was considerably larger. As a result, they have much lower frequencies and per segregating site contributions to variance at present. The larger population size, however, will also increase the mutational input and thus the number of extant segregating sites; so long as selection is sufficiently strong, these effects balance each other such that the per site contribution to variance, counting both segregating and monomorphic sites, remains insensitive to changes in population size (53). In turn, extant segregating mutations under substantially weaker selection are expected to contribute much less variance per site (considering either segregating sites alone or all sites) primarily because of their smaller effect sizes, which is the same reason that applied in the case with a constant population size (see Fig. 2a).

We find support for this verbal argument when we relate the results of our simulations with the findings from GWAS. To do so, we follow the same reasoning that we applied to the case with constant population size (see Discussion). Namely, based on the distribution of variances (Fig. A19a), we would expect sites with selection coefficients around $s \approx 10^{-3}$ to be the first to be discovered in GWAS. Further assuming that such sites account for most associations discovered in GWAS and that their distribution of variances corresponds to $N_e = 5000$, we can use our estimates of v_s for height and BMI to calculate the parameter w^2/n ($= ½N_e v_s$) for these traits. This approach allows us to plot the putative distribution of variances among sites that exceed the study thresholds, v^*, for different selection

coefficients (Fig. A20a & b). Doing so, we find that the observed and fitted distributions are well approximated by the distributions for sites with $s \approx 10^{-3}$, thus supporting our premise that most of the explained variance arises from such sites, and that their distribution of variances is well approximated by assuming a constant population size of $N_e \approx 5000$. Our simulations also suggest that the proportion of variance explained for sites with $s \approx 10^{-3}$ is much greater than the proportion for sites under weaker or stronger selection (Fig. A20c & d), and should therefore also be greater than the total proportion of variance explained by these GWAS. This expectation accords with our findings as well, with our simulations suggesting that the proportion of variance explained for sites with $s \approx 10^{-3}$ is ~40% for height and ~30% for BMI (Fig. A20c & d) compared to a total proportion of ~25% for height and ~5% for BMI in these GWAS (8, 10).

Examining the expected MAF and allelic ages at sites that we predict to have been identified by these GWAS lends further support to our interpretation (Fig. A20e-h). Notably, we find that the MAF for sites with $s \approx 10^{-3}$ that are predicted to have been identified by these studies are similar to those that are observed (Fig. A20e & f). Moreover, when we examine the ages of mutations at detected sites, we find that mutations at sites with $s \approx 10^{-3}$ are predicted to have originated during or shortly before the OoA bottleneck (Fig. A20g & h).

In summary, our analyses suggest that the bulk of associations identified in the GWAS for height and BMI tag segregating mutations with $s \approx 10^{-3}$, which originated shortly before or during the OoA bottleneck. As a result, we would expect the distribution of variances among these sites to be well approximated by our equilibrium predictions corresponding to an effective population of $N_e \approx 5000$. This finding provides an explanation for why our equilibrium predictions fit the findings of GWAS in Europeans, despite our ignoring the dramatic changes in population size during their recent evolutionary past.

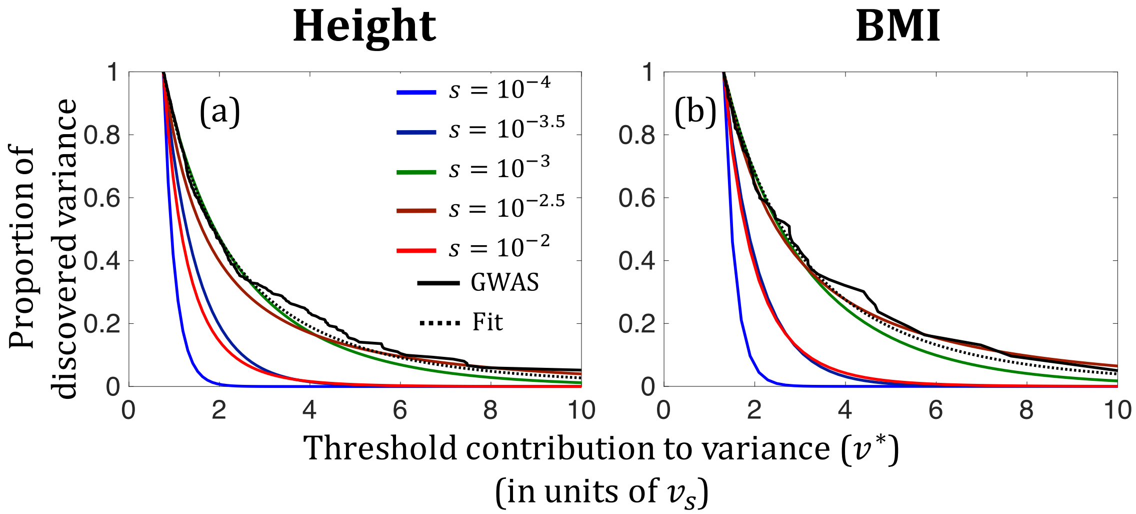

Height
BMI
(a)
(b)
$s = 10^{-4}$
$s = 10^{-3.5}$
$s = 10^{-3}$
$s = 10^{-2.5}$
$s = 10^{-2}$
GWAS
Fit
Proportion of discovered variance
Threshold contribution to variance (v^*)
(in units of v_s)

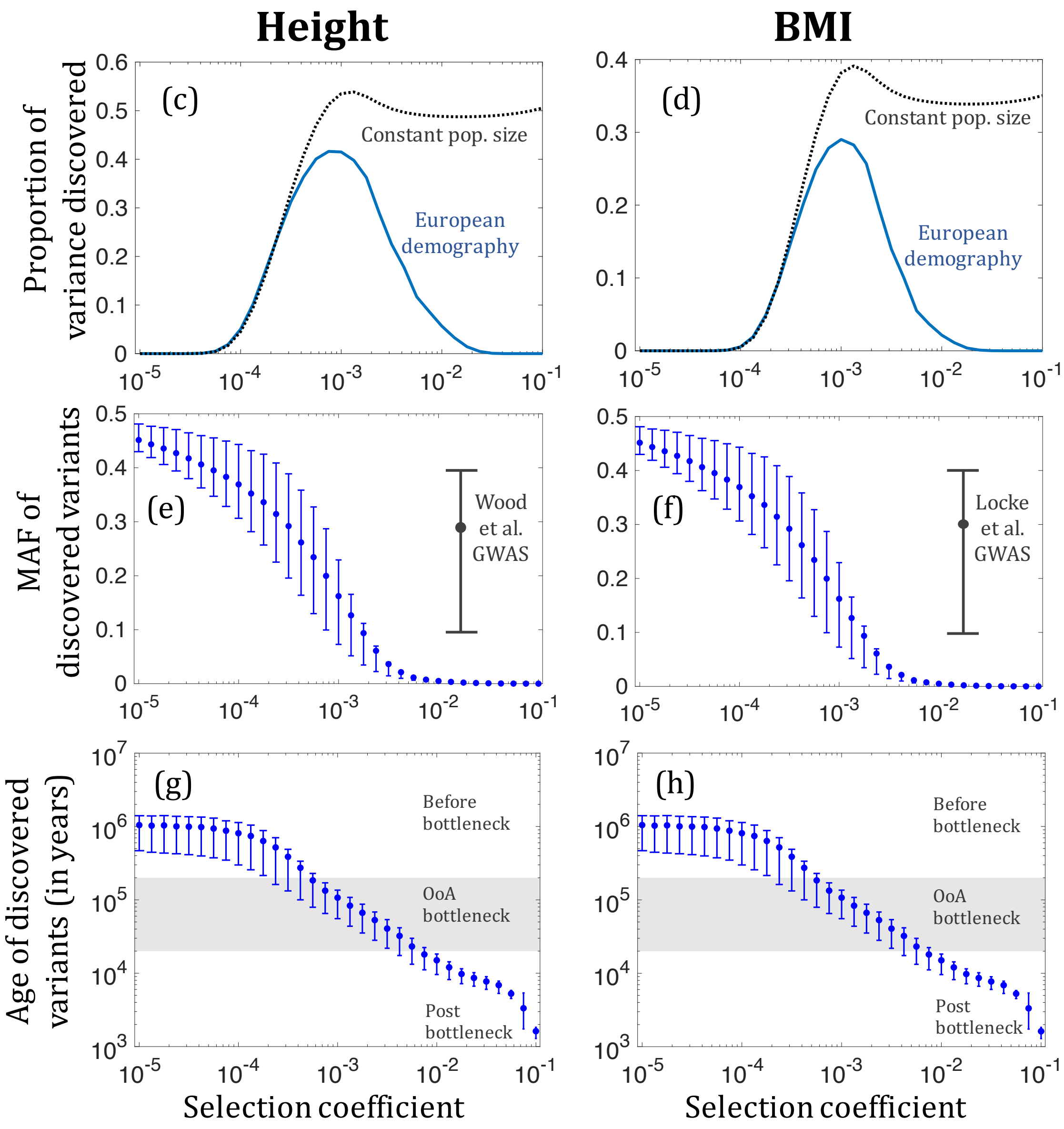

Figure A20. Comparison of the results of simulations with European demography with our inferences and the findings from GWAS for height and BMI (8, 10). (a & b) The distribution of variances among discovered loci. For each selection coefficient, the proportion of variance at the study threshold is set to 1. Simulation-based distributions are in color; the empirical distributions are in solid black; and our inferred fits are in dashed black. (c & d) The expected proportion of variance explained in GWAS as a function of the selection coefficient, based on simulations and on the equilibrium model with a constant population size of $N_e = 5{,}000$. (e & f) Comparison of MAF of discovered sites as a function of selection coefficient in simulations with the MAF observed for GWS associations in GWAS. (g & h) The age of mutations at discovered sites as a function of selection coefficient based on simulations. In (e–h), points correspond to the mean and whiskers span the 1st to 3rd quartiles of the distribution.

10. The effects of genotyping

Another implication of the demographic effects that we discussed in the last section (Section 9) pertains to the reliance on genotyping rather than resequencing in GWAS. As we reviewed in Section 6, current genotyping-based GWAS typically consider only loci with $\text{MAF} > 1\%$, for which imputation is currently quite accurate, at least in Europeans (24). Even if loci below that frequency were imputed with perfect accuracy, however, they would only be detected in a GWAS if they exceed the threshold contribution to variance for that study. Thus, loci at which the minor allele is rare would only be detected if they had very large effect sizes, which in our model implies very strong selection. For example, assuming a constant population size, if a re-sequencing study captured 25% of the heritable variance, a genotyping study with the same sample size would suffer a ≥ 50% decrease in explained heritability only if $S \geq 200$ (Fig. A21a & b)For an effective population size of $2 \cdot 10^4$ for humans (50), that implies an enormous fitness cost of $s \geq 0.5\%$ (in heterozygotes) for the minor allele.

Our results for European demographic history suggest that only a small proportion of genetic variance can arise from loci that fall below the current MAF imputation threshold but have sufficiently large effect sizes to exceed the variance discovery thresholds of current GWAS. To illustrate that, we relied on our simulation results and estimates of v_s for height and BMI, to calculate the proportion of variance arising from sites with $\text{MAF} < 1\%$ and contribution to variance $> v^*$. We find this proportion to be greater than 0 only for selection coefficients between $s = 0.3 \cdot 10^{-2}$ and $s = 2 \cdot 10^{-2}$, but even within this range, such loci account for less than ~6% of the expected variance for height and 2% of the variance for BMI (Fig. A21c & d). This suggests that the common reliance on genotyping in current GWAS for quantitative traits entails minimal loss in the discovery of associations relative to resequencing. Moreover, this is likely to remain the case even when GWAS sizes substantially increase, as long as such increases are accompanied by reasonable increases in the size of imputation panels.

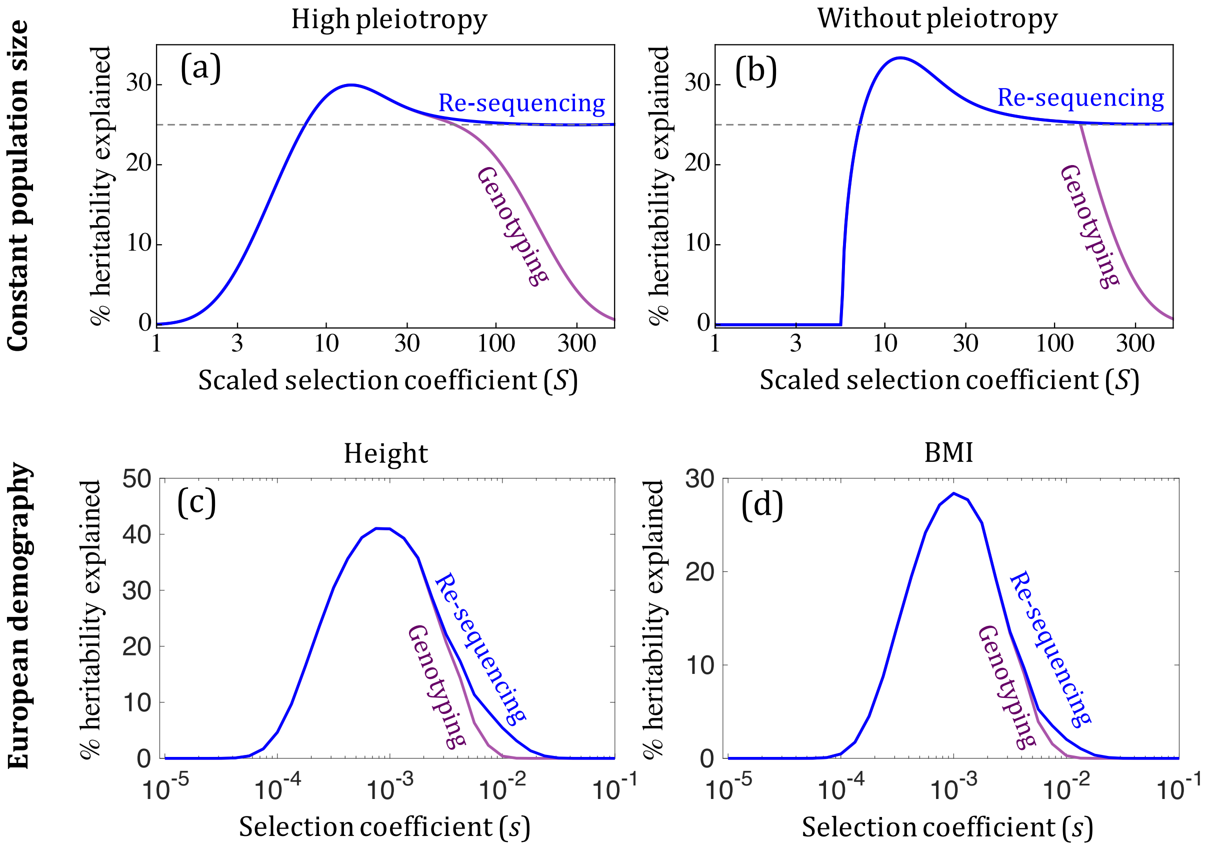

Figure A21. The heritability explained in resequencing and genotyping studies as a function of selection coefficient. (a-b) The case with a constant population size, in the high pleiotropy limit (a) and without pleiotropy (b). The study size was chosen such that a resequencing study would capture 25% of the strongly selected variance: implying a study size of $\sim 16V_P/v_s$ in the highly pleiotropic limit (a), and a study size of $\sim 43V_P/v_s$ without pleiotropy (b). (c-d) The case with European demographic history and high pleiotropy, using our estimates of v_s for height (c) and BMI (d) (see Section 9 for details).

11. Glossary of notation

$\vec{r}$	n-dimensional phenotype
$\mathrm{W}(\vec{r})$	Absolute fitness
w	Scale of selection
n	Number of traits (dimension)
$\vec{a}$	A mutation's n-dimensional effect size
a_1	A mutation's effect size on focal trait
U	Haploid mutation rate per generation
σ^2	Phenotypic variance in a trait
K	Number of segregating sites
$\varphi_n(a_1 \vert a)$	Distribution effect sizes on focal trait conditional on overall effect size
$S = \frac{N\mathrm{a}^2}{2\mathrm{w}^2}$	Scaled selection coefficient
$\eta(a_1 \vert S)$	Distribution of effect sizes on focal trait conditional on S
q	Derived allele frequency
p	Ancestral allele frequency, $p = 1 - q$
$\tau(q \vert S)$	The sojourn time for a mutation with scaled selection coefficient S
v	Contribution to variance from a site $\left(v = 2a_1^2 q(1-q)\right)$
v_s	Expected contribution of a strongly selected site to variance $(v_s = 2w^2/nN)$.
$\mathrm{E}(V \vert S)$	Expected contribution to genetic variance from sites with S
$\mathrm{E}(K \vert S)$	Expected number of segregating sites with S
$\rho(v)$	Density of segregating sites contributing variance v
$\mathrm{G}(v^*)$	Proportion of variance from sites with contribution to variance > v^*
m	GWAS study size
H	Power to identify a locus in GWAS

12. Additional figure

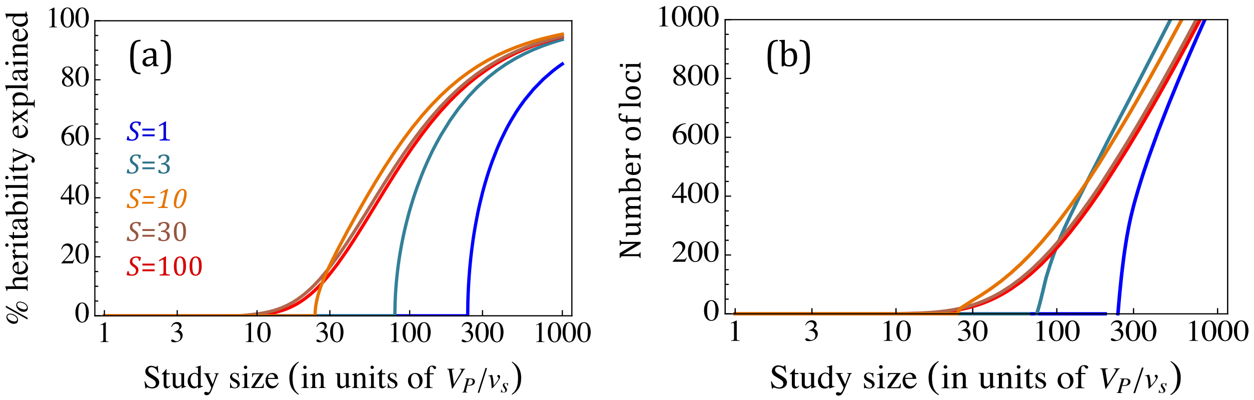

Figure A22. The proportion of heritability (a) and the number of variants per Mbp (b) identified in GWAS as a function of study size, in the case without pleiotropy ($n = 1$); see Section 3 for derivations. This figure is equivalent to Fig. 4 from the main text, which describes the case with pleiotropy ($n \gg 1$).

13. Bibliography

1. Lande R (1975) The maintenance of genetic variability by mutation in a polygenic character with linked loci. *Genet Res* 26(3):221-235.
2. Poon A & Otto SP (2000) Compensating for our load of mutations: Freezing the meltdown of small populations. *Evolution* 54(5):1467-1479.
3. Ewens WJ (2004) *Mathematical population genetics 1: I. Theoretical introduction* (Springer Science & Business Media).
4. Keightley PD & Hill WG (1988) Quantitative genetic variability maintained by mutation-stabilizing selection balance in finite populations. *Genet Res* 52(01):33-43.
5. Kong A*, et al.* (2012) Rate of de novo mutations and the importance of father's age to disease risk. *Nature* 488(7412):471-475.
6. Ward LD & Kellis M (2012) Evidence of abundant purifying selection in humans for recently acquired regulatory functions. *Science* 337(6102):1675-1678.
7. Perry JRB*, et al.* (2014) Parent-of-origin-specific allelic associations among 106 genomic loci for age at menarche. *Nature* 514(7520):92-97.
8. Locke AE*, et al.* (2015) Genetic studies of body mass index yield new insights for obesity biology. *Nature* 518:197-206.
9. Scott RA*, et al.* (2012) Large-scale association analyses identify new loci influencing glycemic traits and provide insight into the underlying biological pathways. *Nat Genet* 44(9):991-1005.
10. Wood AR*, et al.* (2014) Defining the role of common variation in the genomic and biological architecture of adult human height. *Nat Genet* 46(11):1173–1186.
11. Schork NJ, Murray SS, Frazer KA, & Topol EJ (2009) Common vs. Rare allele hypotheses for complex diseases. *Curr Opin Genet Dev* 19(3):212-219.
12. Hunt KA*, et al.* (2013) Negligible impact of rare autoimmune-locus coding-region variants on missing heritability. *Nature* 498(7453):232-235.
13. Do R*, et al.* (2015) Exome sequencing identifies rare ldlr and apoa5 alleles conferring risk for myocardial infarction. *Nature* 518(7537):102-106.
14. Purcell SM*, et al.* (2014) A polygenic burden of rare disruptive mutations in schizophrenia. *Nature* 506(7487):185-190.
15. Chen JA, Peñagarikano O, Belgard TG, Swarup V, & Geschwind DH (2015) The emerging picture of autism spectrum disorder: Genetics and pathology. *Annu Rev Pathol Mech Dis* 10:111-144.
16. Uhlenbeck GE & Ornstein LS (1930) On the theory of the brownian motion. *Phys Rev* 36(5):823.
17. De Vladar HP & Barton N (2014) Stability and response of polygenic traits to stabilizing selection and mutation. *Genetics* 197(2):749-767.
18. Jain K & Stephan W (2017) Rapid adaptation of a polygenic trait after a sudden environmental shift. *Genetics* 207(3).
19. Lande R (1976) Natural selection and random genetic drift in phenotypic evolution. *Evolution* 30(2):314-334.
20. Charlesworth B (2013) Stabilizing selection, purifying selection, and mutational bias in finite populations. *Genetics* 194(4):955-971.
21. Leffler EM*, et al.* (2013) Multiple instances of ancient balancing selection shared between humans and chimpanzees. *Science* 339(6127):1578-1582.
22. Denny JC*, et al.* (2013) Systematic comparison of phenome-wide association study of electronic medical record data and genome-wide association study data. *Nat Biotech* 31(12):1102-1111.

23. Martin G & Lenormand T (2006) A general multivariate extension of fisher's geometrical model and the distribution of mutation fitness effects across species. *Evolution* 60(5):893-907.
24. Sham PC & Purcell SM (2014) Statistical power and significance testing in large-scale genetic studies. *Nat Rev Genet* 15(5):335-346.
25. Dickson SP, Wang K, Krantz I, Hakonarson H, & Goldstein DB (2010) Rare variants create synthetic genome-wide associations. *PLoS Biol* 8(1):e1000294.
26. Evangelou E & Ioannidis JPA (2013) Meta-analysis methods for genome-wide association studies and beyond. *Nat Rev Genet* 14(6):379-389.
27. Visscher PM, Brown MA, Mccarthy MI, & Yang J (2012) Five years of GWAS discovery. *Am J Hum Genet* 90:7-24.
28. Pritchard JK & Przeworski M (2001) Linkage disequilibrium in humans: Models and data. *Am J Hum Genet* 69(1):1-14.
29. The 1000 Genomes Project Consortium (2015) A global reference for human genetic variation. *Nature* 526(7571):68-74.
30. Yang J, *et al.* (2012) Conditional and joint multiple-snp analysis of GWAS summary statistics identifies additional variants influencing complex traits. *Nat Genet* 44(4):369-375.
31. Wray NR, Purcell SM, & Visscher PM (2011) Synthetic associations created by rare variants do not explain most GWAS results. *PLoS Biol* 9(1):e1000579.
32. He M, *et al.* (2015) Meta-analysis of genome-wide association studies of adult height in east asians identifies 17 novel loci. *Hum Mol Genet* 24(6):1791-1800.
33. Anderson CA, Soranzo N, Zeggini E, & Barrett JC (2011) Synthetic associations are unlikely to account for many common disease genome-wide association signals. *PLoS Biol* 9(1):e1000580.
34. Liu JZ, *et al.* (2015) Association analyses identify 38 susceptibility loci for inflammatory bowel disease and highlight shared genetic risk across populations. *Nat Genet* 47(9):979-986.
35. Spain SL & Barrett JC (2015) Strategies for fine-mapping complex traits. *Hum Mol Genet* 24(R1):R111-R119.
36. Zheng J, *et al.* (2017) Haprap: A haplotype-based iterative method for statistical fine mapping using GWAS summary statistics. *Bioinformatics* 33(1):79-86.
37. Liu X, *et al.* (2015) Signatures of natural selection at the FTO (fat mass and obesity associated) locus in human populations. *PLoS One* 10(2):e0117093.
38. Genest C & Remillard B (2008) Validity of the parametric bootstrap for goodness-of-fit testing in semiparametric models. *Ann Inst H Poincare Probab Statist* 44(6):1096-1127.
39. Stute W, Manteiga WG, & Quindimil MP (1993) Bootstrap based goodness-of-fit-tests. *Metrika* 40(1):243-256.
40. Marouli E, *et al.* (2017) Rare and low-frequency coding variants alter human adult height. *Nature* 542(7640):186-190.
41. Grove ML, *et al.* (2013) Best practices and joint calling of the humanexome beadchip: The charge consortium. *PLoS One* 8(7):e68095.
42. Yang J, *et al.* (2015) Genetic variance estimation with imputed variants finds negligible missing heritability for human height and body mass index. *Nat Genet* 47(10):1114-1120.
43. Yang J, *et al.* (2010) Common SNPs explain a large proportion of the heritability for human height. *Nat Genet* 42(7):565-569.
44. Speed D, *et al.* (2017) Reevaluation of snp heritability in complex human traits. *Nat Genet* 49(7):986-992.
45. Schoech A, *et al.* (2017) Quantification of frequency-dependent genetic architectures and action of negative selection in 25 uk biobank traits. *bioRxiv*.

46. Zeng J, *et al.* (2017) Widespread signatures of negative selection in the genetic architecture of human complex traits. *bioRxiv*.
47. Wall JD & Przeworski M (2000) When did the human population size start increasing? *Genetics* 155(4):1865-1874.
48. Coventry A*, et al.* (2010) Deep resequencing reveals excess rare recent variants consistent with explosive population growth. *Nat Commun* 1:131.
49. Tennessen JA, *et al.* (2012) Evolution and functional impact of rare coding variation from deep sequencing of human exomes. *Science* 337(6090):64-69.
50. Schiffels S & Durbin R (2014) Inferring human population size and separation history from multiple genome sequences. *Nat Genet* 46(8):919-925.
51. Gazave E, Chang D, Clark AG, & Keinan A (2013) Population growth inflates the per-individual number of deleterious mutations and reduces their mean effect. *Genetics* 195(3):969-978.
52. Lohmueller KE (2014) The impact of population demography and selection on the genetic architecture of complex traits. *PLoS Genet* 10(5):e1004379.
53. Simons YB, Turchin MC, Pritchard JK, & Sella G (2014) The deleterious mutation load is insensitive to recent population history. *Nat Genet* 46(3):220-224.
54. The International Hapmap 3 Consortium (2010) Integrating common and rare genetic variation in diverse human populations. *Nature* 467(7311):52-58.
